# In Silico Investigation of Mineralocorticoid Receptor Antagonists: Insights into Binding Mechanisms and Structural Dynamics

**DOI:** 10.3390/molecules30061226

**Published:** 2025-03-09

**Authors:** Julia J. Liang, Sara Cao, Andrew Hung, Assam El-Osta, Tom C. Karagiannis, Morag J. Young

**Affiliations:** 1 Epigenomic Medicine Laboratory at prospED Polytechnic, Carlton, VIC 3053, Australia; 2 Epigenetics in Human Health and Disease Program, Baker Heart and Diabetes Institute, 75 Commercial Road, Prahran, VIC 3004, Australia; 3 Department of Microbiology and Immunology, The University of Melbourne, Parkville, VIC 3010, Australia; 4 School of Science, STEM College, RMIT University, Melbourne, VIC 3001, Australia; 5 Baker Department of Cardiometabolic Health, The University of Melbourne, Parkville, VIC 3010, Australia; 6 Department of Diabetes, Central Clinical School, Monash University, Melbourne, VIC 3004, Australia; 7 Department of Medicine and Therapeutics, The Chinese University of Hong Kong, Sha Tin, Hong Kong SAR, China; 8 Hong Kong Institute of Diabetes and Obesity, Prince of Wales Hospital, The Chinese University of Hong Kong, 3/F Lui Che Woo Clinical Sciences Building, 30–32 Ngan Shing Street, Sha Tin, Hong Kong SAR, China; 9 Li Ka Shing Institute of Health Sciences, The Chinese University of Hong Kong, Sha Tin, Hong Kong SAR, China; 10 Biomedical Laboratory Science, Department of Technology, Faculty of Health, University College Copenhagen, 2200 Copenhagen, Denmark; 11 Department of Clinical Pathology, The University of Melbourne, Parkville, VIC 3010, Australia; 12 Cardiovascular Endocrinology Laboratory, Discovery & Preclinical Domain, Baker Heart and Diabetes Institute, Melbourne, VIC 3004, Australia; 13 Department of Medicine (Alfred Health), Central Clinical School, Monash University, Clayton, VIC 3004, Australia; 14 Central Clinical School, Monash University, Melbourne, VIC 3004, Australia

**Keywords:** mineralocorticoid receptor, aldosterone, spironolactone, mineralocorticoid receptor antagonist, docking, molecular dynamics

## Abstract

The mineralocorticoid receptor (MR) is a steroid hormone receptor that plays a key role in regulating sodium and water homeostasis and blood pressure. MR antagonists are a guideline recommended for therapy for the treatment of hypertension and cardiovascular disease but can cause hyperkalaemia. Modelling was performed for binding of the endogenous ligands aldosterone and cortisol and MR antagonist spironolactone to the ligand binding domain (LBD) of the MR. A molecular docking screen of compounds that were structurally similar to known antagonists was performed, leading to the identification of two novel compounds, C79 and E67. Molecular dynamics (MD) assessed the dynamic interactions with C79, E76, endogenous ligands, and spironolactone with the MR ligand binding domain (LBD). Analysis of the protein backbone showed modest changes in the overall structure of the MR LBD in response to binding of antagonists, with movement in helix 12 consistent with previous observations. All ligands tested maintained stable binding within the MR LBD throughout the simulations. Hydrogen bond formation played a more prominent role in the binding of endogenous ligands compared to antagonists. MM-PBSA binding free energy calculations showed that all ligands had similar binding affinities, with binding facilitated by key residues within the binding site. The novel antagonists demonstrated similar binding properties to spironolactone, warranting further evaluation. This study provides insights into the molecular mechanisms of MR activation and inhibition, which can aid in the development of novel therapeutic strategies for cardiovascular diseases.

## 1. Introduction

**Figure 1:**
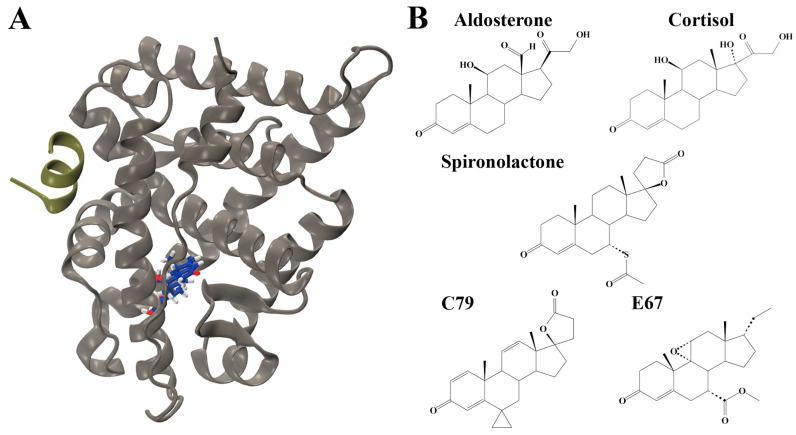
Structures of mineralocorticoid receptor (MR) and ligands. (**A**) Structure of the MR ligand binding domain (LBD) in complex with aldosterone (blue sticks) and nuclear receptor coactivator 1 (NCOA1) peptide (tan green). Oxygen atoms are displayed in red and hydrogen atoms in grey. (**B**) Structures of ligands investigated. Structures of endogenous ligands aldosterone (ALD) and cortisol (CORT) are shown. The antagonist spironolactone (SPL), as well as potential MR antagonists PubChem CID: 46241712 (C79) and Pubchem CID: 153930403 (E67).

The human mineralocorticoid receptor (MR) belongs to the nuclear receptor superfamily, sharing structural homology with other steroid hormone receptors such as the androgen receptor, progesterone receptor, and glucocorticoid receptor [1]. They are composed of three major domains: an N-terminal domain, a central DNA-binding domain, and a C-terminal ligand binding domain (LBD). The MR LBD is complex and multifunctional, having roles in nuclear localisation, interacting with heat shock proteins, dimerisation, and selectively binding hormones to induce transcriptional responses [1]. The structure is highly conserved across species, composed of 11 α-helices and 4 small antiparallel β-strands that are folded into a canonical three-layer helical sandwich with a hydrophobic ligand binding pocket (Figure 1A) [1].

Aldosterone is the physiological ligand of MR in epithelial tissues, such as the kidney and colon in mammals [1,2]. In these tissues, MR activation by aldosterone serves as a critical regulator of salt balance and water homeostasis by directly stimulating the expression of various ionic transporters at the cellular membrane, responsible for transepithelial sodium transport [1]. Cortisol is another physiological ligand of the MR in non-epithelial tissues, such as the heart, immune cells, and regions of the central nervous system. While both aldosterone and cortisol are endogenous ligands for the MR, differences are seen in the binding of the two ligands; despite having similar binding affinities to MR, glucocorticoids, such as cortisol, dissociate more rapidly than aldosterone [3]. Importantly, inappropriate MR activation by either ligand has numerous pathophysiological implications, mainly associated with hypertension and inflammation [4].

MR antagonists (MRAs) have been used widely as antihypertensive agents and cardiovascular protective agents [5,6]. They also bind with a high affinity to the MR but induce a transcriptionally silent receptor conformation [7]. Spironolactone was the first MRA to be developed and is highly potent [8]. However, spironolactone was derived from progesterone and has low selectivity for the MR, also binding to progesterone and androgen receptors [8]. This leads to adverse effects, such as menstrual irregularities in females and gynecomastia in males [8]. Given their widespread clinical benefits, significant efforts have been devoted to the development of novel MRAs that are better tolerated. Eplerenone was subsequently developed and was less prone to causing sex hormone-related adverse effects compared to spironolactone [8]. However, both spironolactone and eplerenone lead to hyperkalaemia in up to 10% of patients, and this is higher in at-risk patients [8]. More recently, non-steroidal MRAs have been developed, such as esaxerenone, balcinrenone, ocedurenone, and finerenone [9]. While non-steroidal MRAs can also competitively inhibit the MR LBD, their mechanisms of action differ from steroidal MRAs in that other aspects of MR signalling are influenced. Non-steroidal MRAs can induce a unique receptor conformation, post-translational modifications, nuclear shuttling, and co-regulator recruitment that results in altered gene expression and biological effects versus the steroidal MRA eplerenone [9].

Previous in silico studies on the MR have explored various aspects of its function [10,11,12,13]. Molecular modelling studies typically employ molecular docking to examine potential binding modes, while molecular dynamics (MD) simulations provide insight into dynamic ligand interactions and conformational changes under physiologically relevant conditions. Pérez-Gordillo et al. used structure-based drug design to identify a novel non-steroidal MRA with a 1,4-dihydropyridine scaffold [10]. Nashev et al. used molecular docking to study the binding orientation of an agonist within the LBD [11]. Furthermore, in silico approaches have been employed to understand the evolutionary basis of MR function. Chimeric structures have been used to investigate the divergent response of agonism and antagonism [12], while another study by Edman et al. examined evolutionary constraints in helix plasticity and used unbiased simulations to elucidate ligand binding pathways [13].

The aim of this study was to model the interactions of MR with steroidal ligands. This included its endogenous ligands (aldosterone and cortisol), the antagonist spironolactone, and potential novel MRAs. A library of compounds that were structurally similar to known steroidal antagonists was built for screening against the MR LBD using molecular docking, identifying two novel compounds for further analysis. The stability and dynamic properties of these ligands were assessed in classical MD simulations, followed by binding free energy calculations with molecular mechanics Poisson–Boltzmann surface area (MM-PBSA). It was shown that the novel compounds exhibit binding properties similar to spironolactone, suggesting their potential as novel antagonists against MR.

## 2. Results and Discussion

### 2.1. Molecular Docking to Identify Novel MR Antagonists

The ligand binding domain (LBD) of the MR was targeted for screening to identify novel MR antagonists (Figure 1A). Using known antagonists as a scaffold—spironolactone (SPL), eplerenone, and canrenone—a similar structure search was performed on PubChem to build a library of structurally similar steroidal compounds. A total of 544 structurally similar compounds were docked to the MR LBD. The endogenous ligands aldosterone (ALD) and cortisol (CORT) were also docked to the LBD. The complete list of docking results is shown in Table S1.

Based on binding affinity, two novel compounds were selected for further analysis. These were PubChem CID: 46241712 (C79) and PubChem CID: 153930403 (E67). These compounds had a stronger binding affinity compared to the controls, with a binding affinity of −10.0 kcal/mol for compound C79 and −9.7 kcal/mol for E67 compared to −7.0 for the known antagonist spironolactone (Table S1).

The docked structures of ALD, CORT, SPL, C79, and E67 served as starting structures for analysis with MD simulations. The chemical structures of these compounds are shown in Figure 1B.

### 2.2. Protein Dynamics of the MR LBD

Classical MD simulations were performed for the MR LBD in its apo form and bound with ALD, CORT, SPL, C79, and E67 to examine the stability of ligand binding and conformational changes that may occur within the protein. Simulations were performed for 200 ns in triplicate.

**Figure 2:**
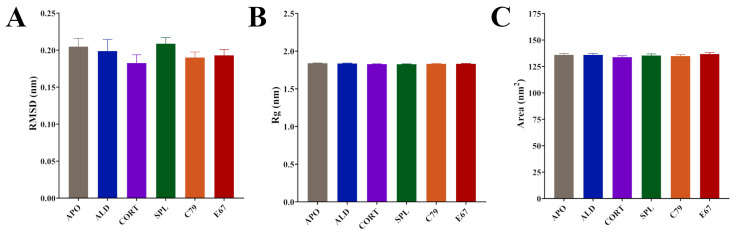
Averaged molecular dynamics (MD) simulation data following equilibration of MR LBD bound with ligands. (**A**) Root meant square deviation (RMSD) of protein backbone. (**B**) Radius of gyration (Rg) of protein backbone. (**C**) Solvent-accessible surface area (SASA) of protein surface. Simulations were performed in triplicate. Averages were calculated from the final 100 ns of each simulation, with coordinates recorded every 10 ps. Data are plotted as mean ± SD.

Time series analyses showed that systems equilibrated after 100 ns (Figure S1). Subsequent calculations were performed after this time point. All systems had similar average RMSD values: 0.20 nm for APO and ALD, 0.18 nm for CORT, 0.21 nm for SPL, and 0.19 for C79 and E67-bound MR (Figure 2A). Average values for the radius of gyration (Rg) were almost identical between systems: 1.84 nm for APO and ALD and 1.83 nm for CORT, SPL, C79, and E67 (Figure 2B). Similarly, solvent-accessible surface area (SASA) values displayed modest differences between systems: 135.9 nm^2^ for APO, 135.9 nm^2^ for ALD, 133.8 nm^2^ for CORT, 135.4 nm^2^ for SPL, 134.8 nm^2^ for C79, and 136.8 nm^2^ for E67 (Figure 2C). The modest changes in these values across systems indicate structural rearrangements that do not significantly alter the overall protein structure upon ligand binding. This aligns with existing observations that MR activation and inhibition primarily involve shifts in helix arrangements and interaction networks within the LBD rather than large-scale conformational changes [4,14].

**Figure 3:**
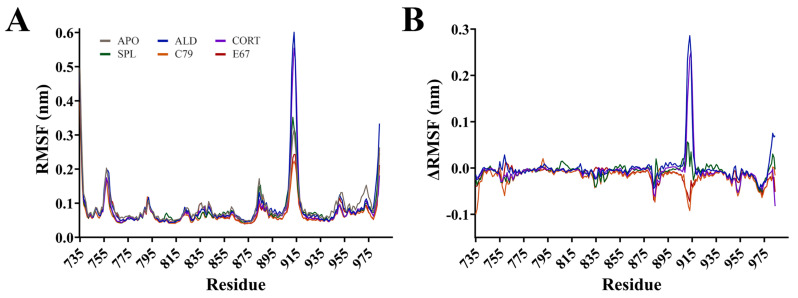
Root mean square fluctuation (RMSF) of the protein backbone for the MR LBD bound with ligands. (**A**) RMSF for protein backbone is shown as an average of three runs following equilibration of the trajectory. (**B**) Difference in RMSF of protein backbone with APO values subtracted from ligand-bound MR LBD.

Root mean square fluctuation (RMSF) calculations measure the flexibility of residues within the protein backbone. RMSF analysis showed that fluctuations mainly occurred in loops located between helices of the MR (Figure 3). The largest fluctuation occurred at residues 910 to 915 in the loop connecting helices 9 and 10, with an RMSF of up to 0.60 nm for ALD and CORT. The ΔRMSF was calculated, where RMSF values of the APO system were subtracted from ligand-bound MR and shown in Figure 3B. The ΔRMSF for residues 910 to 915 is shown in Figure S2A, indicating that for this region of the protein, the endogenous ALD and CORT-bound MR have a larger difference in RMSF compared to the other systems. An ΔRMSF of 0.29 and 0.24 nm at residue 913 was observed for ALD and CORT, respectively. For these residues, SPL had a similar RMSF to APO, while C79 and E67 had a modest suppression in ΔRMSF of −0.09 nm and −0.07 nm at residue 913, respectively. This suggests a potential difference in the dynamic behaviour of the MR in response to antagonists versus receptor activation. This region of the receptor is distant from the ligand binding pocket of MR, consistent with the binding of antagonists to MR influencing movement in regions of the protein beyond the immediate binding site [4].

Backbone fluctuations occurred at residues 755 to 760, located in a connecting loop between helices 1 and 2. RMSF was as high as 0.20 nm in this region, with APO and ALD displaying similar RMSF. There was a modest suppression in RMSF for CORT, SPL, C79, and E67-bound MR with an ΔRMSF of −0.06 nm at residue 759 for C79. Similarly, this region is also not directly involved in ligand binding, but suppression in fluctuations was observed with the binding of most ligands, suggesting stabilisation of the protein–ligand complex. Interestingly, residues 969 to 973 demonstrated a suppression in RMSF compared to APO, with an ΔRMSF of −0.05 nm for all ligands (Figure S2B). These residues are a part of helix 12 (H12), suggesting that this region is stabilised upon ligand binding. This supports previous observations that the apo and agonist conformations of MR differ by the position of the H12 helix, where, in the apo conformation, the H12 helix is folded away from the core of the domain [15]. This conformation allows for the binding of coactivator molecules to the activation function 2 (AF-2) region, a process that is ligand-dependent [4].

### 2.3. Principal Components Analysis of the MR LBD

**Figure 4:**
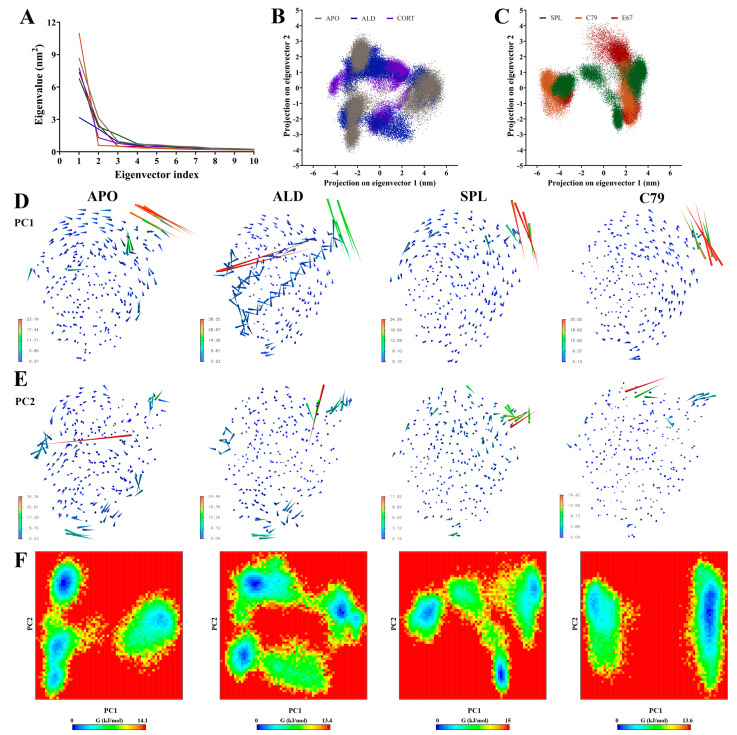
Principal components analysis of MR LBD protein backbone in response to ligand binding. Three independent 100 ns segments of stabilised trajectories were concatenated to analyse concerted motions of the protein backbone. (**A**) Eigenvalues of the covariance matrix. The ligand free protein (APO) is shown in grey, and the MR LBD bound with aldosterone (ALD) in blue, cortisol (CORT) in purple), spironolactone (SPL) in green, C79 in orange, and E67 in red (**B**,**C**) Two-dimensional projection of the combined trajectory on the first two eigenvectors. (**D**) Porcupine plots showing movement along the first principal component (PC1). (**E**) Porcupine plots showing movement along PC2. (**F**) Free energy landscape (FEL) plots calculated from the first two principal components (PC1 and PC2).

Principal components analysis (PCA) was employed to filter large-scale concerted motions from the MD simulation trajectories to study the main motions of the protein complex that occur in response to ligand binding. Analysis of the eigenvalues demonstrated that the first two principal components (PCs) captured the majority of protein motion in the combined trajectories for all systems (Figure 4A). Two-dimensional projection of the first two PCs showed that while all plots occupy a similar subspace, APO, ALD, and CORT (Figure 4B) had a larger spread in values compared to SPL, C79, and E67 (Figure 4C). Particularly for ALD and CORT, the 2D projection indicated more overlap between replicate simulations. This suggests that the binding of ligands can induce different structural rearrangements within the MR LBD.

Porcupine plots visualising the movement of the protein backbone to identify functionally important groups (Figure 4D,E, Figures S3 and S4) showed that for all systems, the majority of movement occurs in the loop between helix H9 and H10 at residues 910 to 915, in line with observations from the RMSF analysis (Figure 3). For ALD and CORT-bound MR, additional movements were observed in helix H10 and H12 in PC1 (Figure 4D and Figure S3). Movements are also observed in this region in PC2 for CORT and E67 systems (Figure S4), suggesting that some spread in the conformational subspace can be attributed to the movement of this region of the LBD.

Free energy landscape (FEL) plots (Figure 4E and Figure S5) align with the above observations, showing a similar spread in the conformational subspace. With the overlapping replicates in ALD and CORT, the FEL plots reveal multiple deep energy wells, suggesting favoured distinct conformations for each replicate simulation, which may be attributed to the flexibility in helix H10 and H12 as observed in the porcupine plots (Figure S3). For C79 and E67, both the 2D projection and FEL plots were divided into two distinct segments, with the FEL plot showing a single deep energy well. This suggests the presence of a single more energetically favourable conformation upon the binding of potential antagonists. While SPL demonstrates some slight overlap between replicates, there also appears to be a preference for fewer distinct energetically favourable conformations compared to ALD and CORT, with some similarity to the FEL plot for APO. This is consistent with the lower receptor stability for antagonist binding.

### 2.4. Dynamic Interactions of Ligands to the MR LBD

**Figure 5:**
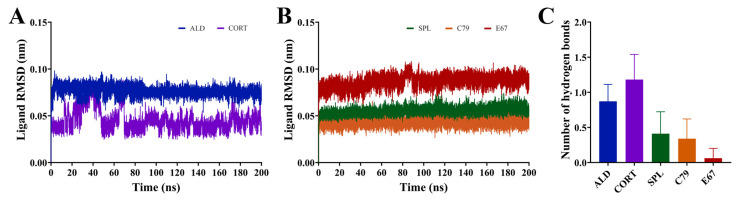
Dynamics of ligands bound to the MR LBD. (**A**,**B**) RMSD of ligands bound to the MR LBD with respect to its initial structure. (**C**) Number of hydrogen bonds formed between ligands and MR LBD throughout the simulation following trajectory. Data are shown as an average of three independent runs, with the number of hydrogen bonds shown as mean ± SD.

All compounds demonstrated stable binding to the MR LBD throughout the simulations, shown by the ligand RMSD with respect to its initial structure in Figure 5A,B. The ligands ALD, SPL, C79, and E67 demonstrated extremely stable binding, with consistent RMSD throughout the simulation indicating minimal movement of the ligands from their initial position throughout the simulations. While CORT remained bound to the LBD for the duration of the simulation, shifting of the ligand occurred within the binding pocket in the initial segment of the simulations, suggesting that its position within the binding pocket may be less stable compared to all other ligands, including ALD. This is consistent with the observation that ALD dissociates at a slower rate than CORT [3].

The average number of hydrogen bonds formed throughout the equilibrated simulation between the ligand and protein was calculated (Figure 5C). It was shown that ALD and CORT interactions appeared to be driven by hydrogen bond formation to a greater degree than for SPL, C79, and E67. ALD and CORT had an average of 0.87 ± 0.24 and 1.18 ± 0.36 hydrogen bonds formed, compared to SPL (0.41 ± 0.31), C79 (0.34 ± 0.28), and E67 (0.06 ± 0.14), which had much smaller numbers of hydrogen bonds formed. This is due to the differences in the chemical structures of the ligands, with ALD and CORT having a greater number of hydrogen donor and acceptor functional groups compared to SPL, C79, and E67 (Figure 1B). The number of unique hydrogen bonds that were formed throughout the simulations is presented in Table 1. Hydrogen bonds were primarily formed with residues N770 and T945. Notably, five distinct hydrogen bonds were observed between both ALD and CORT with residues N770. This is consistent with observations that hydrogen bond formation with N770 and T945 is a requirement for the activation of MR [14]. For SPL and C79, only one hydrogen bond was formed between the ligands and protein for these residues and was absent in E67. This is also consistent with observations that SPL has a weakened potential for hydrogen bonding to these residues, potentially suggesting a mechanism for its antagonistic behaviour [14]. ALD and CORT also formed a hydrogen bond with S767, which was absent in interactions with the antagonists. Hydrogen bond formation with S767 contributes to the stabilisation of the loop preceding the AF-2 helix involved in activation [14].

### 2.5. Binding Free Energy Calculations of Ligands to MR

Binding free energy calculations of ligands to the MR LBD were performed using MM-PBSA. Table 2 shows that all ligands bound to the MD LBD with a similar affinity. For all ligands, van der Waals interactions (Δ*E_vdW_*) were the main driving force for binding due to the hydrophobic residues that predominantly line the binding site. ALD had a Δ*G_binding_* of −31.9 ± 0.3 kcal/mol compared to −29.7 ± 1.4 kcal/mol for CORT. This is consistent with previous observations that while both endogenous ligands have a similar binding affinity, ALD has a slower dissociation rate [3]. C79 and E67 had slightly stronger Δ*G_binding_* (−31.9 ± 0.5 and −32.4 ± 2.4 kcal/mol) compared to SPL (−30.9 ± 2.4 kcal/mol), suggesting their utility as novel MR antagonists. The lower number of hydrogen bonds formed with antagonists (Figure 5C) is consistent with weaker electrostatic interactions (Δ*E_elec_*) compared to the endogenous ligands.

**Figure 6:**
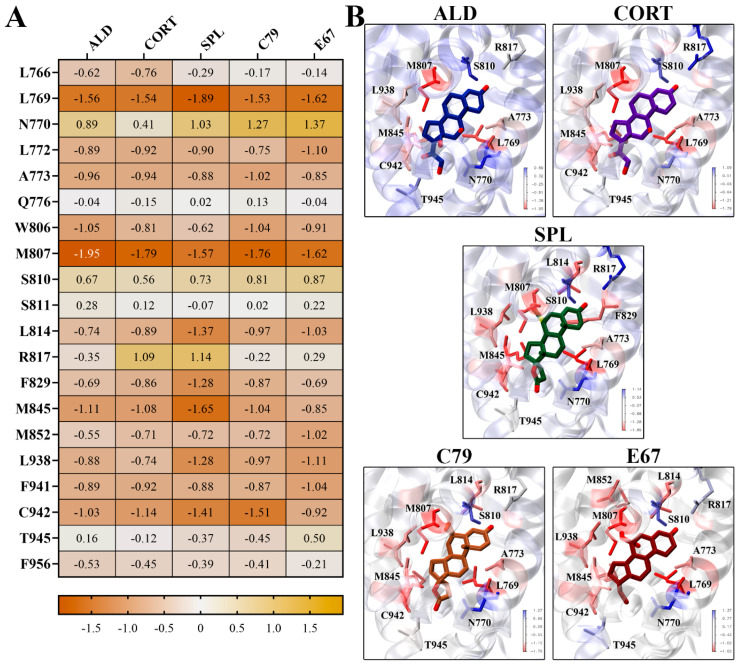
Per-residue contributions to binding energy of compounds to MR LBD. (**A**) Heatmap of key residues’ contribution to binding energy for ligand binding to MR LDB. Energy contributions are shown in kcal/mol as an average of three independent binding free energy calculations using MM-PBSA. (**B**) Ligands binding to MR LBD, with key residues highlighted in stick representation. Protein residues are coloured according to binding energy contributions.

Binding energy contributions were decomposed on a per-residue basis, with the energy contributions of key residues shown in Figure 6. It is shown that residues L769 and M807 had the strongest energy contributions for all ligands. L769 had a strongly favourable energy contribution of −1.89 kcal/mol for SPL, and M807 had an energy contribution of −1.95 kcal/mol to the binding of ALD (Figure 6A). These residues line the binding pocket of the MR LBD in close contact with the ligands (Figure 6B). For SPL, C79, and E67, residue L814 contributes favourably to binding, which is less prominent in binding with ALD and CORT. It should be noted that some key residues involved in water-mediated hydrogen bonds, such as N770, Q776, and S810, had unfavourable energy contributions to ligand binding [14]. This may be attributed to limitations inherent to MM-PBSA methods, where continuum solvent methods may not accurately capture the effects of individual water molecules [16]. Nonetheless, MM-PBSA provides valuable insight into the relative binding affinities and interactions within the MR LBD, showing that the novel antagonists exhibit binding properties comparable to spironolactone.

## 3. Methods

### 3.1. Protein and Ligand Structure Preparation

The structure of human MR LBD was obtained from the RCSB Protein Data Bank (PDB) (ID: 5MWP) [17]. This structure was selected for its resolution and the presence of the nuclear receptor coactivator 1 (NCOA1) peptide in the crystal structure. Missing non-terminal residues were filled in using MODELLER 10.3 [18,19], where five models were generated and the model with the lowest zDOPE scores was selected. The crystal structure contained C808S and C910S mutations, which were reverted to wild type using PyMOL v1.8 [20]. The stereochemical quality of the resulting model was validated using PROCHECK v6.1, showing that 95.4% of residues fell within the core region and 4.6% of residues fell within the allowed region of the Ramachandran plot, indicating good quality [21].

To prepare the library of compounds, a structural similarity search was performed on the National Center of Biotechnology Information PubChem database [22]. Known antagonists were used as scaffolds: spironolactone and its active metabolite canrenone, as well as eplerenone. Duplicate compounds were removed based on canonical SMILES. The Tanimoto scores indicating the structural similarity of the remaining compounds were calculated using MACCS fingerprint analysis using OpenBabel v2.2.3 [23]. Compounds were ranked in descending order according to their Tanimoto score, and compounds with a Tanimoto score of 1, indicating that they were identical structures, were removed. The 3D structures of the remaining compounds were downloaded from PubChem as sdf files. Compounds were energy-minimised with the universal force field and converted to PDBQT format for docking using OpenBabel [23]. The resulting compound library consisted of 90 similar compounds to spironolactone, 187 to eplerenone, and 267 to canrenone. The complete list of compounds screened and their Tanimoto scores are shown in Table S1.

### 3.2. Molecular Docking

Molecular docking was performed using Autodock Vina [24] at an exhaustiveness of 128. The receptor grid was 20 × 20 × 20 Å in size and centred around key residues of the binding site in the MR LBD: N770, Q776, M777, S810, S811, R817, M852, and T945 [17]. Receptor grid coordinates used were X: 6.08, Y: 16.98, Z: 13.89.

### 3.3. Molecular Dynamics Simulations

Molecular dynamics (MD) simulations were performed for ligands bound to the MR LBD using the docked ligands as starting structures. Simulations were performed with GROMACS 2018.3 [25,26] with the CHARMM36 force field [27], and ligand topologies were generated using CGenFF 4.0 [28]. Protein–ligand complexes were solvated with the TIP3P water model [29] in a dodecahedral box with a minimum distance of 1.0 nm between protein atoms and the box edge. Systems were neutralised and salted with 0.15 M NaCl and energy-minimised with the steepest-descent gradient energy method. Equilibration was performed for 100 ps under the canonical (NVT) ensemble at 310 K using a modified Berendsen thermostat [30]. This was followed by 100 ps of equilibration under the isothermal–isobaric (NPT) ensemble at 1.0 bar pressure using the Parrinello–Rahman barostat [31]. The LINCS algorithm [32] was utilised to constrain bond lengths, and the particle-mesh Ewald scheme (PME) [33] was used to calculate long-range electrostatics with a grid spacing of 0.16 nm. Cut-off ratios of 1.2 nm were used for both Coulomb and van der Waals potentials. Production runs were performed for 200 ns in triplicate with a time-step of 2 fs.

### 3.4. Analysis of Simulation Trajectories

VMD 1.9.3 was used to visualise protein complexes [34]. Analysis of trajectories was performed using tools within GROMACS [25,26], including gmx rms for the calculation of root mean square deviation (RMSD), gmx rmsf for the calculation of root mean square fluctuation (RMSF), gmx gyrate for the radius of gyration (Rg), gmx sasa for the solvent-accessible surface area (SASA), and gmx hbond for the number of hydrogen bonds formed between the ligand and protein.

Principal components analysis (PCA) was utilised to study the motions of the MR in response to ligand binding. Three independent 100 ns segments of equilibrated trajectories were concatenated to represent different regions of conformational space around the starting structure. Using gmx covar, a covariance matrix was generated from the atomic fluctuations in the combined trajectory, which was then diagonalised using gmx anaeig to obtain a set of eigenvectors and corresponding eigenvalues. Extreme projections along the first two principal components were visualised as porcupine plots using VMD 1.9.3 [34]. The stability of conformations within each system was estimated using gmx sham for the calculation of free energy landscape (FEL) plots.

The molecular mechanics Poisson–Boltzmann surface area (MM-PBSA) method was used to calculate the binding free energy of ligands to the MR using g_mmpbsa v5.1.2 [35]. Calculations were performed on the final 10 ns of the trajectories in triplicate. Energy contribution terms from electrostatic, van der Waals, and polar solvation terms were calculated using the adaptive Poisson–Boltzmann solver (APBS) v1.3 [36]. Grid spacing was set to 0.05 nm, and values of 80 and 2 were used for the solvent and solute dielectric constants, respectively. Nonpolar energy contributions were approximated by SASA with a probe radius of 0.14 nm. Entropic energy terms were excluded from calculations.

## 4. Conclusions

This study has investigated the interaction between the MR LBD and endogenous ligands aldosterone and cortisol and known and unknown antagonists. Using molecular docking, two potential novel antagonists were identified: C79 and E67. Assessment of the dynamic protein–ligand complexes with MD simulations and MM-PBSA analysis demonstrated that the novel antagonists exhibited similar binding properties comparable to spironolactone, with trends in hydrogen bond formation consistent with antagonist behaviour. Further in vitro and in vivo studies are required to assess the efficacy, selectivity, and safety profile of C79 and E67 as MR antagonists. This work describes a pipeline for the identification of novel MR antagonists and provides new insights into ligand binding mechanisms of the MR, highlighting the differential trends in protein movement and protein–ligand interactions in the binding of endogenous ligands compared to antagonists. This approach can be extended to investigate other steroid hormone receptors as part of the screening process, contributing to the development of novel therapeutics in hypertension and cardiovascular disease.

## Figures and Tables

**Table 1:** Number of unique hydrogen bonds formed between ligands and residues of the MR LBD.

Residue	ALD	CORT	SPL	C79	E67
S767	1	1			
N770	5	5	1	1	
Q776	1	1	1	1	1
S810	1	1	1	1	1
S811			1		
R817		1	1	2	1
A844	1				
M845	1				
T945	3	2	1	1	

**Table 2:** Binding free energy contribution terms from MM-PBSA analysis of ligands binding to the MR LBD variants. Values are reported in kcal/mol as mean ± SD from three replicate simulations.

Energy Terms	ALD	CORT	SPL	C79	E67
Δ*E_vdW_*	−48.1 ± 0.1	−48.9 ± 0.8	−56.3 ± 0.3	−50.6 ± 0.8	−48.3 ± 0.2
Δ*E_elec_*	−10.4 ± 0.2	−9.9 ± 0.5	−6.9 ± 0.2	−3.9 ± 0.2	−5.4 ± 0.4
Δ*G_polar_*	31.1 ± 0.2	33.7 ± 1.5	37.4 ± 0.9	27.1 ± 0.3	26.2 ± 2.7
Δ*G_nonpolar_*	−4.5 ± 0.0	−4.5 ± 0.0	−5.1 ± 0.0	−4.6 ± 0.0	−4.9 ± 0.0
Δ*G_binding_*	−31.9 ± 0.3	−29.7 ± 1.4	−30.9 ± 1.2	−31.9 ± 0.5	−32.4 ± 2.4

Abbreviations: Δ*E_vdW_* = van der Waals interaction, Δ*E_elec_* = electrostatic interaction, Δ*G_polar_* = polar contribution, Δ*G_nonpolar_* = nonpolar contribution to the solvation free energy estimated by solvent-accessible surface area (SASA), Δ*G_binding_* = binding free energy.

## Data Availability

The original contributions presented in the study are included in the article/Supplementary Materials, further inquiries can be directed to the corresponding author.

## Supplementary Materials

The following supporting information can be downloaded at: https://www.mdpi.com/article/10.3390/molecules30061226/s1, Figure S1: Molecular dynamics (MD) simulations of MR LBD bound with ligands; Figure S2: RMSF of protein backbone for regions of interest for MR bound to ligands; Figure S3: Porcupine plots showing movement along the first principal component (PC1) of MR LBD in response to ligand binding; Figure S4: Porcupine plots showing movement along the second principal component (PC2) of MR LBD in response to ligand binding; Figure S5: Free energy landscape (FEL) plots calculated from the first two principal components (PC1 and PC2) of the MR LBD backbone in response to ligand binding; Table S1: Molecular docking of structurally similar compounds of spironolactone, canrenone, and eplerenone to the ligand binding domain of the human mineralocorticoid receptor.
