# Supplementary material for: In Silico Investigation of Mineralocorticoid Receptor Antagonists: Insights into Binding Mechanisms and Structural Dynamics
Source: Molecules. 2025 Mar 9;30(6):1226. doi: 10.3390/molecules30061226 (PMC11944687; doi:10.3390/molecules30061226)
Supplement: Supplementary file 1 [file molecules-30-01226-s001.zip › molecules-3477553-supplementary.pdf]

Supplementary information to:

In Silico Investigation of Mineralocorticoid Receptor Antagonists: Insights into Binding Mechanisms and Structural Dynamics

Julia J. Liang 1,2, Sara Cao 1,3, Andrew Hung 4, Assam El-Osta 2,5,6,7,8,9,10, Tom C. Karagiannis 1,2,3,5,11,*,† and Morag J. Young 5,12,13,14,†

1 Epigenomic Medicine Laboratory at prospED Polytechnic, Carlton, VIC 3053, Australia;
2 Epigenetics in Human Health and Disease Program, Baker Heart and Diabetes Institute, 75 Commercial Road, Prahran, VIC 3004, Australia
3 Department of Microbiology and Immunology, The University of Melbourne, Parkville, VIC 3010, Australia
4 School of Science, STEM College, RMIT University, Melbourne, VIC 3001, Australia;
5 Baker Department of Cardiometabolic Health, The University of Melbourne, Parkville, VIC 3010, Australia
6 Department of Diabetes, Central Clinical School, Monash University, Melbourne, VIC 3004, Australia
7 Department of Medicine and Therapeutics, The Chinese University of Hong Kong, Sha Tin, Hong Kong SAR, China
8 Hong Kong Institute of Diabetes and Obesity, Prince of Wales Hospital, The Chinese University of Hong Kong, 3/F Lui Che Woo Clinical Sciences Building, 30–32 Ngan Shing Street, Sha Tin, Hong Kong SAR, China
9 Li Ka Shing Institute of Health Sciences, The Chinese University of Hong Kong, Sha Tin, Hong Kong SAR, China
10 Biomedical Laboratory Science, Department of Technology, Faculty of Health, University College Copenhagen, 2200 Copenhagen, Denmark
11 Department of Clinical Pathology, The University of Melbourne, Parkville, VIC 3010, Australia
12 Cardiovascular Endocrinology Laboratory, Discovery & Preclinical Domain, Baker Heart and Diabetes Institute, Melbourne, VIC 3004, Australia
13 Department of Medicine (Alfred Health), Central Clinical School, Monash University, Clayton, VIC 3004, Australia
14 Central Clinical School, Monash University, Melbourne, VIC 3004, Australia
* Correspondence: karat@unimelb.edu.au; Tel.: +61-3-8532-1290; Fax: +61-3-8532-1100
† These authors contributed equally to this work.

Contents

Table S1: Molecular docking of structurally similar compounds of spironolactone, canrenone, and eplerenone to the ligand binding domain of the human mineralocorticoid receptor. **3**

Figure S1: Molecular dynamics (MD) simulations of MR LBD bound with ligands. **50**

Figure S2: RMSF of protein backbone for regions of interest for MR bound to ligands **51**

Figure S3: Porcupine plots showing movement along the first principal component (PC1) of MR LBD in response to ligand binding. **52**

Figure S4: Porcupine plots showing movement along the second principal component (PC2) of MR LBD in response to ligand binding. **52**

Figure S5: Free energy landscape (FEL) plots calculated from the first two principal components (PC1 and PC2) of the MR LBD backbone in response to ligand binding. **53**

Table S1: Molecular docking of structural similar compounds of spironolactone, canrenone, and eplerenone to the ligand binding domain of the human mineralocorticoid receptor.

Pubchem CID	Similarity group	Ligand ID	Compound name	Tanimoto score	Binding affinity (kcal/mol)
**5839**	Control	-	Aldosterone	control	-5.7
**5754**	Control	-	Cortisol	control	-7.4
**5833**	Spironolactone	ligand_1	Spironolactone	1.000000	-7.0
**65660**	Spironolactone	ligand_2	Mespirenone	0.975610	-9.0
**44289941**	Spironolactone	ligand_3	S-[(7R,17R)-10,13-dimethyl-3,5'-dioxospiro[2,6,7,8,9,14,15,16-octahydro-1H-cyclopenta[a]phenanthrene-17,2'-oxolane]-7-yl] 2,2-dimethylpropanethioate	0.975610	-1.8
**44279704**	Spironolactone	ligand_4	S-[(2S,3'S,5'S,18'R)-7',11'-dimethyl-5,14'-dioxospiro[oxolane-2,6'-pentacyclo[8.8.0.02,7.03,5.011,16]octadec-15-ene]-18'-yl] ethanethioate	0.975610	-3.3
**101917204**	Spironolactone	ligand_5	S-[(2R,8R,9S,10R,13S,14S,17R)-10,13-dimethyl-3,5'-dioxospiro[2,8,9,11,12,14,15,16-octahydro-1H-cyclopenta[a]phenanthrene-17,2'-oxolane]-2-yl] ethanethioate	0.975610	-2.4
**89932559**	Spironolactone	ligand_6	S-[(7R,8R,9R,10R,13S,14R,17R)-8,10,13-trimethyl-3,5'-dioxospiro[1,2,6,7,9,11,12,14,15,16-decahydrocyclopenta[a]phenanthrene-17,2'-oxolane]-7-yl] ethanethioate	0.975610	-5.4
**10138430**	Spironolactone	ligand_7	S-[(7R,10R,13S,15S,16S,17S)-10,13,15,16-tetramethyl-3,5'-dioxospiro[2,6,7,8,9,11,12,14,15,16-decahydro-1H-cyclopenta[a]phenanthrene-17,2'-oxolane]-7-yl] ethanethioate	0.975610	-2.8
**44290289**	Spironolactone	ligand_8	S-[(7R,17R)-10,13-dimethyl-3,5'-dioxospiro[7,8,9,14,15,16-hexahydro-6H-cyclopenta[a]phenanthrene-17,2'-oxolane]-7-yl] ethanethioate	0.975000	-3.1
**452291**	Spironolactone	ligand_9	7-(Acetylthio)-17-hydroxy-3-oxopregn-4-ene-21-carboxylic acid gamma-lactone	0.975000	-9.3
**90764620**	Spironolactone	ligand_10	S-[(7S,17R)-10-methyl-3,5'-dioxospiro[1,2,4,7,8,9,11,12,13,14,15,16-dodecahydrocyclopenta[a]phenanthrene-17,2'-oxolane]-7-yl] ethanethioate	0.975000	-6.1

**122680942**	Spironolactone	ligand_11	S-[(8R,9S,10R,13S,14S,17R)-3,10,13-trimethyl-5'-oxospiro[1,2,3,6,7,8,9,11,12,14,15,16-dodecahydrocyclopenta[a]phenanthrene-17,2'-oxolane]-7-yl] ethanethioate	0.975000	-7.1
**44280017**	Spironolactone	ligand_12	S-[(3S,5R,10R,13S,15S,16S)-2,17-dimethyl-5',6-dioxospiro[hexacyclo[9.8.0.02,8.03,5.012,17.013,15]nonadec-7-ene-16,2'-oxolane]-10-yl] ethanethioate	0.952381	-4.1
**71045199**	Spironolactone	ligand_13	(7R,8R,9S,10R,13S,14S,17R)-7-(1-hydroxyethylsulfanyl)-10,13-dimethylspiro[2,6,7,8,9,11,12,14,15,16-decahydro-1H-cyclopenta[a]phenanthrene-17,5'-oxolane]-2',3-dione	0.952381	-7.5
**91446451**	Spironolactone	ligand_14	S-[(7R,8R,9S,10R,13S,14S,17S)-10,13-dimethyl-2',3-dioxospiro[2,6,7,8,9,11,12,14,15,16-decahydro-1H-cyclopenta[a]phenanthrene-17,3'-oxolane]-7-yl] ethanethioate	0.952381	-8.3
**142467810**	Spironolactone	ligand_15	S-[(9R)-8-[2-[(2R)-2-ethyl-5-oxooxolan-2-yl]ethyl]-4a-methyl-2-oxo-3,4,4b,5,6,7,8,8a,9,10-decahydrophenanthren-9-yl] ethanethioate	0.952381	-5.4
**154975089**	Spironolactone	ligand_16	S-[(7R,17R)-10,13-dimethyl-3,5'-dioxospiro[2,6,7,8,9,11,12,14,15,16-decahydro-1H-cyclopenta[a]phenanthrene-17,2'-oxolane]-7-yl] 2-(7-ethyl-3-methyl-3-bicyclo[3.3.1]nonanyl)ethanethioate	0.952381	-5.1
**54670339**	Spironolactone	ligand_17	S-[(7R,17S)-10,13-dimethyl-3,5'-dioxospiro[2,6,7,8,9,11,12,14,15,16-decahydro-1H-cyclopenta[a]phenanthrene-17,2'-oxolane]-7-yl] propanethioate	0.952381	-4.9
**162325**	Spironolactone	ligand_18	7alpha-Thiomethylspironolactone	0.951220	-6.4
**44290260**	Spironolactone	ligand_19	S-[(7R,17R)-10,13-dimethyl-3,5'-dioxospiro[7,8,9,14,15,16-hexahydro-6H-cyclopenta[a]phenanthrene-17,2'-oxolane]-7-yl] 2,2-dimethylpropanethioate	0.951220	-1.6
**148681169**	Spironolactone	ligand_20	S-[(7R,17R)-10,13-dimethyl-3,5'-dioxospiro[2,6,7,8,9,11,12,14,15,16-decahydro-1H-cyclopenta[a]phenanthrene-17,2'-oxolane]-7-yl] 2-(1-adamantyl)ethanethioate	0.951220	2.0
**16128858**	Spironolactone	ligand_21	(7R,10S,13S,17R)-7-cyclopentylsulfanyl-10,13-dimethylspiro[2,6,7,8,12,14,15,16-octahydro-1H-cyclopenta[a]phenanthrene-17,5'-oxolane]-2',3-dione	0.950000	-6.9

**154975090**	Spironolactone	ligand_22	S-[(7R,17R)-10,13-dimethyl-3,5'-dioxospiro[2,6,7,8,9,11,12,14,15,16-decahydro-1H-cyclopenta[a]phenanthrene-17,2'-oxolane]-7-yl] 5-ethyl-3-methylideneheptanethioate	0.930233	-4.9
**10049015**	Spironolactone	ligand_23	S-[(2S,7'S,11'R,18'S)-7',11'-dimethyl-5,14'-dioxospiro[oxolane-2,6'-pentacyclo[8.8.0.02,7.03,5.011,16]octadec-15-ene]-18'-yl] propanethioate	0.930233	-5.3
**10143836**	Spironolactone	ligand_24	S-[(7R,10R,13S,15R,16R,17S)-10,13,15,16-tetramethyl-3,5'-dioxo-4-propanoylsulfanylspiro[2,6,7,8,9,11,12,14,15,16-decahydro-1H-cyclopenta[a]phenanthrene-17,2'-oxolane]-7-yl] propanethioate	0.930233	1.2
**10276244**	Spironolactone	ligand_25	S-[(7S,10R,13S,15S,16S,17S)-10,13,15,16-tetramethyl-3,5'-dioxospiro[2,6,7,8,9,11,12,14,15,16-decahydro-1H-cyclopenta[a]phenanthrene-17,2'-oxolane]-7-yl] propanethioate	0.930233	-2.1
**101917202**	Spironolactone	ligand_26	S-[[(2R,7R,8R,9S,10R,13S,14S,17R)-7-acetylsulfanyl-10,13-dimethyl-3,5'-dioxospiro[2,6,7,8,9,11,12,14,15,16-decahydro-1H-cyclopenta[a]phenanthrene-17,2'-oxolane]-2-yl]methyl] ethanethioate	0.930233	-0.5
**129011910**	Spironolactone	ligand_27	S-[(7R,8R,9S,10R,13S,14S,17R)-10,13-dimethyl-3,5'-dioxospiro[2,6,7,8,9,11,12,14,15,16-decahydro-1H-cyclopenta[a]phenanthrene-17,2'-oxolane]-7-yl]sulfanyl ethanethioate	0.930233	-7.6
**122683347**	Spironolactone	ligand_28	(8R,9S,10R,13S,14S,15S,16S,17S)-10,13,15,16-tetramethyl-7-methylsulfanylspiro[7,8,9,11,12,14,15,16-octahydro-6H-cyclopenta[a]phenanthrene-17,5'-oxolane]-2',3-dione	0.928571	-5.8
**138625447**	Spironolactone	ligand_29	(1'R,2'S,3'R,5S,5'R,7'S,10'S,11'R)-5',7',11'-trimethyl-18'-methylsulfanylspiro[oxolane-5,6'-pentacyclo[8.8.0.02,7.03,5.011,16]octadeca-12,15-diene]-2,14'-dione	0.928571	-4.9
**60092427**	Spironolactone	ligand_30	S-[(7R,8R,9S,10R,13S,14S,17R)-10,13-dimethyl-5'-methylidene-3-oxospiro[2,6,7,8,9,11,12,14,15,16-decahydro-1H-cyclopenta[a]phenanthrene-17,2'-oxolane]-7-yl] ethanethioate	0.926829	-7.3
**16128860**	Spironolactone	ligand_31	methyl 2-[(7R,10S,13S,17R)-10,13-dimethyl-3,5'-dioxospiro[2,6,7,8,12,14,15,16-octahydro-1H-cyclopenta[a]phenanthrene-17,2'-oxolane]-7-yl]sulfanylacetate	0.909091	-6.0

**166788051**	Spironolactone	ligand_32	S-[(2R,3S,4S,4aR)-4-butyl-4a-methyl-7-oxo-3-[(5R,8R)-2-oxo-1-oxaspiro[4.4]nonan-8-yl]-1,2,3,4,5,6-hexahydronaphthalen-2-yl] ethanethioate	0.909091	-6.2
**10645717**	Spironolactone	ligand_33	S-[(1R,3aS,3bR,4R,9aR,9bS,11aS)-9a,11a-dimethyl-5',7-dioxospiro[2,3,3a,3b,4,5,9,9b,10,11-decahydroindeno[4,5-h]isochromene-1,2'-oxolane]-4-yl] ethanethioate	0.909091	-9.1
**78426519**	Spironolactone	ligand_34	S-[(2R,7R,8R,9S,10R,13S,14S,17R)-2-hydroxy-10,13-dimethyl-3,5'-dioxospiro[2,6,7,8,9,11,12,14,15,16-decahydro-1H-cyclopenta[a]phenanthrene-17,2'-oxolane]-7-yl] ethanethioate	0.909091	-6.9
**102195067**	Spironolactone	ligand_35	methyl 2-[(7R,8R,9S,10R,13S,14S,17R)-10,13-dimethyl-3,5'-dioxospiro[2,6,7,8,9,11,12,14,15,16-decahydro-1H-cyclopenta[a]phenanthrene-17,2'-oxolane]-7-yl]sulfanylacetate	0.909091	-7.2
**16128377**	Spironolactone	ligand_36	(7R,10S,13S,17R)-7-ethylsulfanyl-10,13-dimethylspiro[2,6,7,8,12,14,15,16-octahydro-1H-cyclopenta[a]phenanthrene-17,5'-oxolane]-2',3-dione	0.906977	-6.6
**101917205**	Spironolactone	ligand_37	S-[[(2R,8R,9S,10R,13S,14S,17R)-10,13-dimethyl-3,5'-dioxospiro[2,8,9,11,12,14,15,16-octahydro-1H-cyclopenta[a]phenanthrene-17,2'-oxolane]-2-yl]methyl] ethanethioate	0.906977	-0.4
**102195070**	Spironolactone	ligand_38	(7R,8R,9S,10R,13S,14S,17R)-7-butylsulfanyl-10,13-dimethylspiro[2,6,7,8,9,11,12,14,15,16-decahydro-1H-cyclopenta[a]phenanthrene-17,5'-oxolane]-2',3-dione	0.906977	-7.6
**119472**	Spironolactone	ligand_39	7alpha-Thiospironolactone	0.904762	-6.1
**23141**	Spironolactone	ligand_40	Spiroxasone	0.904762	-9.6
**88887233**	Spironolactone	ligand_41	S-[(7R,8R,9R,10R,13S,14R,17R)-8,10,13-trimethyl-5'-methylidene-3-oxospiro[1,2,6,7,9,11,12,14,15,16-decahydrocyclopenta[a]phenanthrene-17,2'-oxolane]-7-yl] ethanethioate	0.904762	-5.5
**139495113**	Spironolactone	ligand_42	(7R,8R,9S,10R,13S,14S,17R)-10,13-dimethyl-7-methylsulfanylspiro[1,2,3,6,7,8,9,11,12,14,15,16-dodecahydrocyclopenta[a]phenanthrene-17,5'-oxolane]-2'-one	0.902439	-8.4

**58834207**	Spironolactone	ligand_43	S-[(10S,13S,17R)-4'-fluoro-10,13-dimethyl-3,5'-dioxospiro[2,6,7,8,12,14,15,16-octahydro-1H-cyclopenta[a]phenanthrene-17,2'-oxolane]-7-yl] ethanethioate	0.888889	-7.0
**88269226**	Spironolactone	ligand_44	S-[(10S,13S,17R)-4'-(fluoromethyl)-10,13-dimethyl-3,5'-dioxospiro[2,6,7,8,12,14,15,16-octahydro-1H-cyclopenta[a]phenanthrene-17,2'-oxolane]-7-yl] ethanethioate	0.888889	-3.1
**169435114**	Spironolactone	ligand_45	4-Bromo-spironolactone	0.888889	-2.8
**142888379**	Spironolactone	ligand_46	(1R,11R,16R)-6,10-dimethylspiro[18-thiapentacyclo[14.2.1.01,6.07,15.010,14]nonadec-7-ene-11,5'-oxolane]-2',3,17-trione	0.883721	-7.3
**102195068**	Spironolactone	ligand_47	(7R,8R,9S,10R,13S,14S,17R)-10,13-dimethyl-7-prop-2-enylsulfanylspiro[2,6,7,8,9,11,12,14,15,16-decahydro-1H-cyclopenta[a]phenanthrene-17,5'-oxolane]-2',3-dione	0.883721	-8.0
**102195069**	Spironolactone	ligand_48	(7R,8R,9S,10R,13S,14S,17R)-10,13-dimethyl-7-prop-2-ynylsulfanylspiro[2,6,7,8,9,11,12,14,15,16-decahydro-1H-cyclopenta[a]phenanthrene-17,5'-oxolane]-2',3-dione	0.883721	-7.6
**12832212**	Spironolactone	ligand_49	[(7R,8R,9S,10R,13S,14S,17S)-7-acetylsulfanyl-10,13-dimethyl-3-oxo-1,2,6,7,8,9,11,12,14,15,16,17-dodecahydrocyclopenta[a]phenanthren-17-yl] acetate	0.880952	-9.0
**148476225**	Spironolactone	ligand_50	S-[(10S,13S,17S)-10-(hydroxymethyl)-13,15-dimethyl-3,5'-dioxospiro[7,8,9,11,12,14,15,16-octahydro-6H-cyclopenta[a]phenanthrene-17,2'-oxolane]-7-yl] ethanethioate	0.869565	-8.6
**101355476**	Spironolactone	ligand_51	methyl (2E)-2-[(3R,3aS,5aS,6R,8S,9S,9aR,9bS)-8,9-bis(acetylsulfanyl)-6-ethyl-3a,6-dimethyl-5'-oxospiro[2,4,5,5a,8,9,9a,9b-octahydro-1H-cyclopenta[a]naphthalene-3,2'-oxolane]-7-ylidene]acetate	0.866667	-1.1
**69974814**	Spironolactone	ligand_52	(7R,8R,9S,10R,13S,14S,17S)-10,13-dimethyl-7-pentylsulfanylspiro[2,6,7,8,9,11,12,14,15,16-decahydro-1H-cyclopenta[a]phenanthrene-17,3'-oxolane]-2',3-dione	0.866667	-7.4

**101355477**	Spironolactone	ligand_53	methyl (2E)-2-[(3R,3aS,5aS,6R,9R,9aR,9bS)-9-acetylsulfanyl-6-ethyl-3a,6-dimethyl-5'-oxospiro[2,4,5,5a,8,9,9a,9b-octahydro-1H-cyclopenta[a]naphthalene-3,2'-oxolane]-7-ylidene]acetate	0.866667	-2.3
**254194**	Spironolactone	ligand_54	Testoster-3,11-dione, 9-methylthio-	0.860465	-8.2
**91744186**	Spironolactone	ligand_55	17-Acetoxy-6alpha-acetylthio-19-nor-17alpha-pregn-4-en-20-yn-3-one	0.860465	-5.9
**88756206**	Spironolactone	ligand_56	[(8R,9R,10R,13S,14S)-7-acetylsulfanyl-10,13-dimethyl-1,2-dimethylidene-3-oxo-6,7,8,9,11,12,14,15,16,17-decahydrocyclopenta[a]phenanthren-17-yl] acetate	0.860465	-7.4
**10949493**	Spironolactone	ligand_57	methyl (8S,9S,10R,13S,14S,17S)-7-acetylsulfanyl-10,13-dimethyl-3-oxo-1,2,6,7,8,9,11,12,14,15,16,17-dodecahydrocyclopenta[a]phenanthrene-17-carboxylate	0.860465	-8.8
**104628**	Spironolactone	ligand_58	Ethyl hydrogen 7alpha-(acetylthio)-17alpha-hydroxy-3-oxopregn-4-ene-21,21-dicarboxylate gamma-lactone	0.851064	-1.0
**87990308**	Spironolactone	ligand_59	[(8R,9S,10S,13S,14S,17R)-2,10,13-trimethyl-3-oxo-17-[(3S)-2-oxooxolan-3-yl]sulfanylcarbonyl-7,8,9,11,12,14,15,16-octahydro-6H-cyclopenta[a]phenanthren-17-yl] propanoate	0.851064	-0.6
**88026024**	Spironolactone	ligand_60	[(8R,9S,10R,13S,14S,16R,17R)-10,13,16-trimethyl-3-oxo-17-(2-oxooxolan-3-yl)sulfanylcarbonyl-7,8,9,11,12,14,15,16-octahydro-6H-cyclopenta[a]phenanthren-17-yl] propanoate	0.851064	-2.4
**60098441**	Spironolactone	ligand_61	S-[(10S,13S,17S)-10-(hydroxymethyl)-13,15-dimethyl-3,5'-dioxospiro[2,6,7,8,9,11,12,14,15,16-decahydro-1H-cyclopenta[a]phenanthrene-17,2'-oxolane]-7-yl] ethanethioate	0.851064	-4.8
**89142593**	Spironolactone	ligand_62	[(7R,8R,9S,10R,13S,14S,17S)-7-acetylsulfanyl-10,13-dimethyl-3-oxo-1,2,6,7,8,9,11,12,14,15,16,17-dodecahydrocyclopenta[a]phenanthren-17-yl] but-3-enoate	0.840909	-7.1
**163456320**	Spironolactone	ligand_63	4-[(7R,8S,9S,10R,13R,14S)-7-acetylsulfanyl-10-methyl-3-oxo-1,2,6,7,8,9,11,12,14,15,16,17-dodecahydrocyclopenta[a]phenanthren-13-yl]butyl acetate	0.840909	-5.4
**22212734**	Spironolactone	ligand_64	7alpha-Methylthiotestosterone acetate	0.837209	-8.9

**16627194**	Spironolactone	ligand_65	(7R,10R,13S,17R)-10,13-dimethyl-7-(oxiran-2-ylmethylsulfanyl)spiro[2,6,7,8,9,11,12,14,15,16-decahydro-1H-cyclopenta[a]phenanthrene-17,5'-oxolane]-2',3-dione	0.829787	-7.7
**88809774**	Spironolactone	ligand_66	3-[(7R,8R,9S,10R,13S,14S,17R)-17-acetyloxy-7-acetylsulfanyl-10,13-dimethyl-3-oxo-2,6,7,8,9,11,12,14,15,16-decahydro-1H-cyclopenta[a]phenanthren-17-yl]propyl acetate	0.822222	-3.6
**88809889**	Spironolactone	ligand_67	3-[(7R,8R,9R,10R,13S,14S,17R)-17-acetyloxy-7-acetylsulfanyl-13-methyl-3-oxo-1,2,6,7,8,9,10,11,12,14,15,16-dodecahydrocyclopenta[a]phenanthren-17-yl]propyl acetate	0.822222	-3.6
**162324**	Spironolactone	ligand_68	Tmsl S-oxide	0.816327	-7.3
**169502192**	Spironolactone	ligand_69	7alpha-Sulfenic-spironolactone	0.812500	-1.0
**22805718**	Spironolactone	ligand_70	3-[(7R,8R,9S,10R,13S,14S,17R)-17-acetyloxy-10,13-dimethyl-3-oxo-7-propanoylsulfanyl-2,6,7,8,9,11,12,14,15,16-decahydro-1H-cyclopenta[a]phenanthren-17-yl]propyl propanoate	0.804348	-3.2
**88810078**	Spironolactone	ligand_71	3-[(7R,8R,9R,10R,13S,14S,17R)-17-acetyloxy-13-methyl-3-oxo-7-propanoylsulfanyl-1,2,6,7,8,9,10,11,12,14,15,16-dodecahydrocyclopenta[a]phenanthren-17-yl]propyl propanoate	0.804348	-3.2
**57515749**	Spironolactone	ligand_72	7-(Acetylthio)-17-hydroxy-3-oxo-pregn-4-ene-21-carboxylic acid	0.787234	-8.2
**57125321**	Spironolactone	ligand_73	3-[(7R,8R,9S,10R,13S,14S,17R)-7-acetylsulfanyl-17-hydroxy-10,13-dimethyl-3-oxo-7,8,9,11,12,14,15,16-octahydro-6H-cyclopenta[a]phenanthren-17-yl]propanoic acid	0.787234	-7.4
**88103223**	Spironolactone	ligand_74	3-[(6R,7S,8R,9S,10R,13S,14S,17R)-7-acetylsulfanyl-17-hydroxy-6,10,13-trimethyl-3-oxo-2,6,7,8,9,11,12,14,15,16-decahydro-1H-cyclopenta[a]phenanthren-17-yl]propanoic acid	0.787234	-7.6
**88378317**	Spironolactone	ligand_75	(8R,9S,10S,13S,14S,17S)-7-acetylsulfanyl-17-(2-carboxyethyl)-17-hydroxy-10,13-dimethyl-3-oxo-2,6,7,8,9,11,12,14,15,16-decahydro-1H-cyclopenta[a]phenanthrene-1-carboxylic acid	0.787234	-6.2

**16128532**	Spironolactone	ligand_76	(7R,10S,13S,17R)-7-ethylsulfinyl-10,13-dimethylspiro[2,6,7,8,12,14,15,16-octahydro-1H-cyclopenta[a]phenanthrene-17,5'-oxolane]-2',3-dione	0.784314	-6.4
**129093698**	Spironolactone	ligand_77	S-[(7R,8R,9S,10R,13S,14S,17R)-17-hydroxy-10,13-dimethyl-3-oxo-17-(3-oxobutyl)-2,6,7,8,9,11,12,14,15,16-decahydro-1H-cyclopenta[a]phenanthren-7-yl] ethanethioate	0.782609	-6.9
**154975104**	Spironolactone	ligand_78	[(7R,17S)-10,13,17-trimethyl-3-oxo-7-sulfanyl-2,6,7,8,9,11,12,14,15,16-decahydro-1H-cyclopenta[a]phenanthren-17-yl] propanoate	0.782609	-3.3
**169445662**	Spironolactone	ligand_79	3-[(7R,8R,9S,10R,13S,14S,17S)-17-hydroxy-10,13-dimethyl-7-methylsulfanyl-3-oxo-2,6,7,8,9,11,12,14,15,16-decahydro-1H-cyclopenta[a]phenanthren-17-yl]propanoic acid	0.770833	-7.9
**122196586**	Spironolactone	ligand_80	(8R,9S,10R,13S,14S,17R)-10,13-dimethyl-7-methylsulfonylspiro[2,6,7,8,9,11,12,14,15,16-decahydro-1H-cyclopenta[a]phenanthrene-17,5'-oxolane]-2',3-dione	0.769231	-6.9
**88060194**	Spironolactone	ligand_81	3-[(7R,8R,9R,10R,13S,14S,17R)-7-acetylsulfanyl-17-hydroxy-13-methyl-3-oxo-1,2,6,7,8,9,10,11,12,14,15,16-dodecahydrocyclopenta[a]phenanthren-17-yl]propanoic acid	0.765957	-8.1
**88810076**	Spironolactone	ligand_82	3-[(6R,7S,8R,9S,10R,13S,14S,17R)-7-acetylsulfanyl-17-hydroxy-6,10,13-trimethyl-3-oxo-2,6,7,8,9,11,12,14,15,16-decahydro-1H-cyclopenta[a]phenanthren-17-yl]propyl acetate	0.755102	-3.8
**88810137**	Spironolactone	ligand_83	3-[(7R,8R,9R,10R,13S,14S,17R)-7-acetylsulfanyl-17-hydroxy-13-methyl-3-oxo-1,2,6,7,8,9,10,11,12,14,15,16-dodecahydrocyclopenta[a]phenanthren-17-yl]propyl acetate	0.755102	-5.4
**88810217**	Spironolactone	ligand_84	[(7R,8R,9S,10R,13S,14S,17R)-7-acetylsulfanyl-17-(3-hydroxypropyl)-10,13-dimethyl-3-oxo-2,6,7,8,9,11,12,14,15,16-decahydro-1H-cyclopenta[a]phenanthren-17-yl] acetate	0.755102	-6.0
**88810400**	Spironolactone	ligand_85	3-[(7R,8R,9S,10R,13S,14S,17R)-7-acetylsulfanyl-17-hydroxy-10,13-dimethyl-3-oxo-2,6,7,8,9,11,12,14,15,16-decahydro-1H-cyclopenta[a]phenanthren-17-yl]propyl acetate	0.755102	-4.0

**88810546**	Spironolactone	ligand_86	[(7R,8R,9R,10R,13S,14S,17R)-7-acetylsulfanyl-17-(3-hydroxypropyl)-13-methyl-3-oxo-1,2,6,7,8,9,10,11,12,14,15,16-dodecahydrocyclopenta[a]phenanthren-17-yl] acetate	0.755102	-6.6
**88810071**	Spironolactone	ligand_87	3-[(7R,8R,9R,10R,13S,14S,17R)-17-hydroxy-13-methyl-3-oxo-7-propanoylsulfanyl-1,2,6,7,8,9,10,11,12,14,15,16-dodecahydrocyclopenta[a]phenanthren-17-yl]propyl propanoate	0.740000	-4.9
**88810629**	Spironolactone	ligand_88	3-[(7R,8R,9S,10R,13S,14S,17R)-17-hydroxy-10,13-dimethyl-3-oxo-7-propanoylsulfanyl-2,6,7,8,9,11,12,14,15,16-decahydro-1H-cyclopenta[a]phenanthren-17-yl]propyl propanoate	0.740000	-3.0
**88810717**	Spironolactone	ligand_89	3-[(6R,7S,8R,9S,10R,13S,14S,17R)-7-acetylsulfanyl-17-hydroxy-6,10,13-trimethyl-3-oxo-2,6,7,8,9,11,12,14,15,16-decahydro-1H-cyclopenta[a]phenanthren-17-yl]propyl butanoate	0.740000	-1.5
**88810276**	Spironolactone	ligand_90	3-[(7R,8R,9R,10R,13S,14S,17R)-7-acetylsulfanyl-13-ethyl-17-hydroxy-3-oxo-1,2,6,7,8,9,10,11,12,14,15,16-dodecahydrocyclopenta[a]phenanthren-17-yl]propyl acetate	0.720000	-5.3
**443872**	Eplerenone	ligand_1	Eplerenone	1.000000	-8.2
**44433505**	Eplerenone	ligand_2	Epierenone	0.977273	-5.8
**59595030**	Eplerenone	ligand_3	methyl (1R,2S,9R,14R,15S,17S)-2,4,15-trimethyl-5,5'-dioxospiro[18-oxapentacyclo[8.8.0.01,17.02,7.011,15]octadec-6-ene-14,2'-oxolane]-9-carboxylate	0.977273	-7.9
**59595032**	Eplerenone	ligand_4	methyl (1R,2S,9R,14R,15R,17R)-2,15,16-trimethyl-5,5'-dioxospiro[18-oxapentacyclo[8.8.0.01,17.02,7.011,15]octadec-6-ene-14,2'-oxolane]-9-carboxylate	0.977273	-5.7
**89300856**	Eplerenone	ligand_5	methyl 2-[(1R,9S,14R,17R)-2,15-dimethyl-5,5'-dioxospiro[18-oxapentacyclo[8.8.0.01,17.02,7.011,15]octadec-6-ene-14,2'-oxolane]-9-yl]acetate	0.977273	-5.6
**90692293**	Eplerenone	ligand_6	methyl (2S,4R,6R,11S)-5,18-dimethyl-5',15-dioxospiro[3-oxapentacyclo[8.8.0.02,4.05,9.013,18]octadec-12-ene-6,2'-oxolane]-11-carboxylate	0.977273	-4.9

**153272938**	Eplerenone	ligand_7	methyl (1R,2S,9R,10R,11S,14R,15S,17R)-2,15-dimethyl-5'-methylidene-5-oxospiro[18-oxapentacyclo[8.8.0.01,17.02,7.011,15]octadec-6-ene-14,2'-oxolane]-9-carboxylate	0.977273	-7.7
**90962424**	Eplerenone	ligand_8	methyl (1R,9R,14R,17R)-2-methyl-5,5'-dioxospiro[18-oxapentacyclo[8.8.0.01,17.02,7.011,15]octadec-7-ene-14,2'-oxolane]-9-carboxylate	0.976744	-3.5
**153100278**	Eplerenone	ligand_9	methyl (1R,9R,10R,11S,14R,15S,17R)-15-methyl-5,5'-dioxospiro[18-oxapentacyclo[8.8.0.01,17.02,7.011,15]octadec-6-ene-14,2'-oxolane]-9-carboxylate	0.976744	-8.7
**59773177**	Eplerenone	ligand_10	(1R,2S,9R,10R,11S,14R,15S,17R)-9-(2-methoxy-2-propan-2-yloxyacetyl)-2,15-dimethylspiro[18-oxapentacyclo[8.8.0.01,17.02,7.011,15]octadec-6-ene-14,5'-oxolane]-2',5-dione	0.955556	-6.4
**60000062**	Eplerenone	ligand_11	methyl (2S,4R,5R,6R,11R,18R)-5,18-dimethyl-5'-methylidene-15-oxospiro[3-oxapentacyclo[8.8.0.02,4.05,9.013,18]octadec-13-ene-6,2'-oxolane]-11-carboxylate	0.955556	-4.9
**89301459**	Eplerenone	ligand_12	methyl 2-[(2S,4R,5R,6R,11S,18R)-5,18-dimethyl-5',15-dioxospiro[3-oxapentacyclo[8.8.0.02,4.05,9.013,18]octadec-13-ene-6,2'-oxolane]-11-yl]acetate	0.955556	-6.5
**141908334**	Eplerenone	ligand_13	methyl (1R,2S,9S,14S,15S,17R)-2,15-dimethyl-5,6'-dioxospiro[18-oxapentacyclo[8.8.0.01,17.02,7.011,15]octadec-6-ene-14,2'-oxane]-9-carboxylate	0.954545	-3.9
**9867987**	Eplerenone	ligand_14	propan-2-yl (1R,2S,9R,10R,11S,14R,15S,17R)-2,15-dimethyl-5,5'-dioxospiro[18-oxapentacyclo[8.8.0.01,17.02,7.011,15]octadec-6-ene-14,2'-oxolane]-9-carboxylate	0.954545	-8.3
**58606557**	Eplerenone	ligand_15	(2S,10R,11S,14R,15S,17R)-9-acetyl-2,15-dimethylspiro[18-oxapentacyclo[8.8.0.01,17.02,7.011,15]octadec-6-ene-14,5'-oxolane]-2',5-dione	0.953488	-5.1

**59771784**	Eplerenone	ligand_16	(1R,2S,9R,10R,11S,14R,15S,17R)-2,15-dimethyl-9-[(E)-4-oxopent-2-enoyl]spiro[18-oxapentacyclo[8.8.0.01,17.02,7.011,15]octadec-6-ene-14,5'-oxolane]-2',5-dione	0.953488	-6.5
**88059704**	Eplerenone	ligand_17	methyl (1R,2S,9R,10R,11S,14S,15S,17R)-2,15-dimethyl-3',5-dioxospiro[18-oxapentacyclo[8.8.0.01,17.02,7.011,15]octadec-6-ene-14,1'-cyclopentane]-9-carboxylate	0.953488	-6.7
**89301453**	Eplerenone	ligand_18	(1R,9R,14R,17R)-2,9,15-trimethylspiro[18-oxapentacyclo[8.8.0.01,17.02,7.011,15]octadec-6-ene-14,5'-oxolane]-2',5-dione	0.953488	-5.1
**88892714**	Eplerenone	ligand_19	methyl (1R,2S,9R,10R,12R,14R)-2,12-dimethyl-5-oxo-12-[(2R)-5-oxo-2-propyloxolan-2-yl]-15-oxatetracyclo[8.5.0.01,14.02,7]pentadec-6-ene-9-carboxylate	0.934783	-6.6
**142813128**	Eplerenone	ligand_20	(1R,2S,9R,14R,15S,17R)-9-(3-methoxyoxiran-2-yl)-2,15-dimethylspiro[18-oxapentacyclo[8.8.0.01,17.02,7.011,15]octadec-6-ene-14,5'-oxolane]-2',5-dione	0.934783	-9.2
**139445116**	Eplerenone	ligand_21	methyl (1S,3R,5S,6R,9S,10R,11R,13Z)-5-methyl-5'-oxo-13-(2-oxohexylidene)spiro[2-oxatetracyclo[8.4.0.01,3.05,9]tetradecane-6,2'-oxolane]-11-carboxylate	0.933333	-7.4
**89374470**	Eplerenone	ligand_22	methyl 2-[(1R,9S,14S)-2,15-dimethyl-3',5-dioxospiro[18-oxapentacyclo[8.8.0.01,17.02,7.011,15]octadec-6-ene-14,1'-cyclopentane]-9-yl]acetate	0.931818	-6.9
**159479**	Eplerenone	ligand_23	6,7-Epoxycanrenone	0.930233	-8.0
**9864083**	Eplerenone	ligand_24	(1R,2S,8R,10S,11S,12S,15R,16S,18R)-2,16-dimethylspiro[19-oxahexacyclo[9.8.0.01,18.02,7.08,10.012,16]nonadec-6-ene-15,5'-oxolane]-2',5-dione	0.930233	-9.6
**9951061**	Eplerenone	ligand_25	(1R,2S,8R,10S,11S,12S,15R,16S,18R)-2,16-dimethylspiro[19-oxahexacyclo[9.8.0.01,18.02,7.08,10.012,16]nonadeca-3,6-diene-15,5'-oxolane]-2',5-dione	0.930233	-9.3

**23257014**	Eplerenone	ligand_26	(1R,2S,10S,11S,14R,15S,17R)-2,15-dimethylspiro[18-oxapentacyclo[8.8.0.01,17.02,7.011,15]octadeca-6,8-diene-14,5'-oxolane]-2',5-dione	0.930233	-6.8
**59068516**	Eplerenone	ligand_27	(1R,14R,17S)-2,15-dimethylspiro[18-oxapentacyclo[8.8.0.01,17.02,7.011,15]octadec-6-ene-14,5'-oxolane]-2',5-dione	0.930233	-4.8
**59116722**	Eplerenone	ligand_28	(2S,9R,10R,11R,14R,17R)-9-acetyl-2-methylspiro[18-oxapentacyclo[8.8.0.01,17.02,7.011,15]octadec-6-ene-14,5'-oxolane]-2',5-dione	0.930233	-6.2
**89300861**	Eplerenone	ligand_29	(1R,9R,14R,17R)-2,15-dimethyl-5,5'-dioxospiro[18-oxapentacyclo[8.8.0.01,17.02,7.011,15]octadec-6-ene-14,2'-oxolane]-9-carbaldehyde	0.930233	-4.3
**91147075**	Eplerenone	ligand_30	(1R,14R)-2,15-dimethylspiro[18-oxapentacyclo[8.8.0.01,17.02,7.011,15]octadec-7-ene-14,5'-oxolane]-2',5-dione	0.930233	-5.5
**10270275**	Eplerenone	ligand_31	(1R,2S,15R,16S,18R)-2,16-dimethylspiro[19-oxahexacyclo[9.8.0.01,18.02,7.08,10.012,16]nonadeca-6,8(10)-diene-15,5'-oxolane]-2',5-dione	0.930233	-4.6
**10665833**	Eplerenone	ligand_32	(1R,2S,4R,10R,11S,14S,15R,18S)-10,14-dimethylspiro[3-oxapentacyclo[9.7.0.02,4.05,10.014,18]octadeca-5,8-diene-15,5'-oxolane]-2',7-dione	0.930233	-7.6
**154699545**	Eplerenone	ligand_33	methyl (1R,2S,9S,10R,11S,14R,15S,17R)-8-hydroxy-2,15-dimethyl-5,5'-dioxospiro[18-oxapentacyclo[8.8.0.01,17.02,7.011,15]octadec-6-ene-14,2'-oxolane]-9-carboxylate	0.914894	-8.3
**59086368**	Eplerenone	ligand_34	methyl (1R,2S,9R,14R,15S,17S)-5-ethoxy-2,15-dimethyl-5'-oxospiro[18-oxapentacyclo[8.8.0.01,17.02,7.011,15]octadeca-5,7-diene-14,2'-oxolane]-9-carboxylate	0.914894	-2.5
**59595028**	Eplerenone	ligand_35	methyl (1R,2S,9R,14R,15S,17S)-4-hydroxy-2,15-dimethyl-5,5'-dioxospiro[18-oxapentacyclo[8.8.0.01,17.02,7.011,15]octadec-6-ene-14,2'-oxolane]-9-carboxylate	0.914894	-5.7

**89374475**	Eplerenone	ligand_36	methyl 2-[(1R,9S)-11-ethyl-2,12-dimethyl-12-[(2R)-2-methyl-5-oxooxolan-2-yl]-5-oxo-15-oxatetracyclo[8.5.0.01,14.02,7]pentadec-6-en-9-yl]acetate	0.914894	-7.4
**91558403**	Eplerenone	ligand_37	Methyl 2,15-dimethyl-5,5'-dioxospiro[18-oxapentacyclo[8.8.0.01,17.02,7.011,15]octadec-6-ene-14,2'-oxolane]-9-carboxylate;1,1,1-trichloroethane	0.914894	-10.1
**57345821**	Eplerenone	ligand_38	(2'R,4aS,4bR,5aR,6aS,9aS,9bR,10R)-methyl 4a,6a-dimethyl-2,4'-dioxo-3,4,4a,4',5a,5',6,6a,8,9,9a,9b,10,11-tetradecahydro-2H,3'H-spiro[cyclopenta[1,2]phenanthro[4,4a-b]oxirene-7,2'-furan]-10-carboxylate	0.914894	-9.0
**58834260**	Eplerenone	ligand_39	(1R,2S,9R,14R,15S,17S)-9-butanoyl-2,15-dimethylspiro[18-oxapentacyclo[8.8.0.01,17.02,7.011,15]octadec-6-ene-14,5'-oxolane]-2',5-dione	0.911111	-7.1
**58834277**	Eplerenone	ligand_40	(1R,3S,5S,6S,7S,9S,12R,19S)-5,19-dimethyl-12-propylspiro[2-oxahexacyclo[9.8.0.01,3.05,10.07,9.014,19]nonadec-14-ene-6,5'-oxolane]-2',16-dione	0.911111	-4.2
**58834279**	Eplerenone	ligand_41	(1R,2S,9R,14R,15S,17S)-2,15-dimethyl-9-propylspiro[18-oxapentacyclo[8.8.0.01,17.02,7.011,15]octadec-6-ene-14,5'-oxolane]-2',5-dione	0.911111	-8.6
**91216406**	Eplerenone	ligand_42	(1R,3R,5S,6S,7S,9S,12R,19S)-5,19-dimethyl-12-propylspiro[2-oxahexacyclo[9.8.0.01,3.05,10.07,9.014,19]nonadec-13-ene-6,5'-oxolane]-2',16-dione	0.911111	-6.1
**91479513**	Eplerenone	ligand_43	(1R,2S,9R,14R,15S,17R)-2,15-dimethyl-9-propylspiro[18-oxapentacyclo[8.8.0.01,17.02,7.011,15]octadec-7-ene-14,5'-oxolane]-2',5-dione	0.911111	-6.2
**10274316**	Eplerenone	ligand_44	methyl (2S,9R,14S,15R,17R)-14-(3-methoxy-3-oxopropyl)-2,15-dimethyl-5-oxo-18-oxapentacyclo[8.8.0.01,17.02,7.011,15]octadec-6-ene-9-carboxylate	0.911111	-7.0
**10291798**	Eplerenone	ligand_45	[(1R,2S,8R,10S,11S,12S,15S,16S,18R)-2,15,16-trimethyl-5-oxo-19-oxahexacyclo[9.8.0.01,18.02,7.08,10.012,16]nonadec-6-en-15-yl] formate	0.909091	-8.7

**59769804**	Eplerenone	ligand_46	2-[(1R,2S,9R,10R,11S,14R,15S,17R)-2,15-dimethyl-5,5'-dioxospiro[18-oxapentacyclo[8.8.0.01,17.02,7.011,15]octadec-6-ene-14,2'-oxolane]-9-yl]-2-oxoacetaldehyde	0.909091	-8.2
**91196808**	Eplerenone	ligand_47	(1R,2S,9R,14R,15S,17R)-2,15-dimethyl-5,5'-dioxospiro[18-oxapentacyclo[8.8.0.01,17.02,7.011,15]octadec-7-ene-14,2'-oxolane]-9-carboxylic acid	0.909091	-4.0
**134990130**	Eplerenone	ligand_48	methyl (3S,7R,8S,9S,10R,11R,13S,14S,17R)-3,11-diacetyloxy-10,13-dimethyl-5'-oxospiro[1,2,3,4,7,8,9,11,12,14,15,16-dodecahydrocyclopenta[a]phenanthrene-17,2'-oxolane]-7-carboxylate	0.906977	6.9
**134990996**	Eplerenone	ligand_49	methyl (7S,8S,9S,10R,11R,13S,14S,17R)-11-acetyloxy-10,13-dimethyl-3,5'-dioxospiro[2,6,7,8,9,11,12,14,15,16-decahydro-1H-cyclopenta[a]phenanthrene-17,2'-oxolane]-7-carboxylate	0.906977	1.3
**90823959**	Eplerenone	ligand_50	methyl (7S,8S,9S,10R,11R,13S)-11-acetyloxy-10,13-dimethyl-3,5'-dioxospiro[2,4,7,8,9,11,12,14,15,16-decahydro-1H-cyclopenta[a]phenanthrene-17,2'-oxolane]-7-carboxylate	0.906977	-1.8
**90895695**	Eplerenone	ligand_51	methyl (7S,10R,11R,13S)-11-methoxy-10,13-dimethyl-3,5'-dioxospiro[2,4,7,8,9,11,12,14,15,16-decahydro-1H-cyclopenta[a]phenanthrene-17,2'-oxolane]-7-carboxylate	0.906977	-4.9
**142894693**	Eplerenone	ligand_52	methyl (3S,7S,11R,17R)-11-acetyloxy-3,10,13-trimethyl-5'-oxospiro[1,2,3,4,7,8,9,11,12,14,15,16-dodecahydrocyclopenta[a]phenanthrene-17,2'-oxolane]-7-carboxylate	0.906977	1.3
**58834287**	Eplerenone	ligand_53	methyl (1R,2S,8S,9R,15S)-8-fluoro-2,15-dimethyl-5,5'-dioxospiro[18-oxapentacyclo[8.8.0.01,17.02,7.011,15]octadec-6-ene-14,2'-oxolane]-9-carboxylate	0.895833	-8.4
**90834119**	Eplerenone	ligand_54	methyl (1R,2S,9R,15S)-8-fluoro-2,15-dimethyl-5,5'-dioxospiro[18-oxapentacyclo[8.8.0.01,17.02,7.011,15]octadec-7-ene-14,2'-oxolane]-9-carboxylate	0.895833	-7.7

**58834262**	Eplerenone	ligand_55	methyl (1'R,3'S,4S,5'S,7'S,9'S,12'R,19'S)-5',19'-dimethyl-2,16'-dioxospiro[1,3-dioxolane-4,6'-2-oxahexacyclo[9.8.0.01,3.05,10.07,9.014,19]nonadec-14-ene]-12'-carboxylate	0.895833	-2.0
**59595031**	Eplerenone	ligand_56	methyl (1R,2S,9R,14R,15R,17R)-16-hydroxy-2,15-dimethyl-5,5'-dioxospiro[18-oxapentacyclo[8.8.0.01,17.02,7.011,15]octadec-6-ene-14,2'-oxolane]-9-carboxylate	0.895833	-1.9
**89006736**	Eplerenone	ligand_57	methyl 2-[(1R,9S,14R,17R)-4-hydroxy-2,15-dimethyl-5,5'-dioxospiro[18-oxapentacyclo[8.8.0.01,17.02,7.011,15]octadec-6-ene-14,2'-oxolane]-9-yl]acetate	0.895833	-5.1
**89301458**	Eplerenone	ligand_58	methyl 2-[(1R,2S,8R,9S,14R,15S,17R)-8-hydroxy-2,15-dimethyl-5,5'-dioxospiro[18-oxapentacyclo[8.8.0.01,17.02,7.011,15]octadec-6-ene-14,2'-oxolane]-9-yl]acetate	0.895833	-5.3
**164085531**	Eplerenone	ligand_59	methyl (1'R,3'R,5'S,7'S,12'R,19'S)-5',19'-dimethyl-2,16'-dioxospiro[1,3-dioxolane-4,6'-2-oxahexacyclo[9.8.0.01,3.05,10.07,9.014,19]nonadeca-8,14-diene]-12'-carboxylate	0.895833	-5.0
**9954307**	Eplerenone	ligand_60	ethyl (1R,2S,9R,10R,11S,14R,15S,17R)-2,15-dimethyl-5,5'-dioxospiro[18-oxapentacyclo[8.8.0.01,17.02,7.011,15]octadec-6-ene-14,2'-oxolane]-9-carboxylate	0.893617	-8.5
**90738416**	Eplerenone	ligand_61	ethyl (1R,2S,9R,10R,14R,17R)-2,15-dimethyl-5,5'-dioxospiro[18-oxapentacyclo[8.8.0.01,17.02,7.011,15]octadec-7-ene-14,2'-oxolane]-9-carboxylate	0.893617	-4.2
**24764751**	Eplerenone	ligand_62	3-methylbutyl (1R,2S,9R,10R,11R,14R,15S)-2-methyl-5,5'-dioxospiro[18-oxapentacyclo[8.8.0.01,17.02,7.011,15]octadec-6-ene-14,2'-oxolane]-9-carboxylate	0.893617	-7.2
**58870627**	Eplerenone	ligand_63	methyl (1R,2S,9R,14S,15S,17R)-2,14,15-trimethyl-5-oxo-14-propanoyloxy-18-oxapentacyclo[8.8.0.01,17.02,7.011,15]octadec-6-ene-9-carboxylate	0.891304	-7.3

**59067653**	Eplerenone	ligand_64	(2S,5R,11R,18R)-11-ethyl-5,18-dimethylspiro[3-oxapentacyclo[8.8.0.02,4.05,9.013,18]octadec-13-ene-6,5'-oxolane]-2',15-dione	0.891304	-6.2
**91341383**	Eplerenone	ligand_65	methyl (1R,2S,9R,14S,15S,17R)-2,14,15-trimethyl-5-oxo-14-propanoyloxy-18-oxapentacyclo[8.8.0.01,17.02,7.011,15]octadec-7-ene-9-carboxylate	0.891304	-7.7
**142980353**	Eplerenone	ligand_66	methyl (1R,9R,11S,17R)-2,15-dimethyl-5-oxo-14-propanoyloxy-18-oxapentacyclo[8.8.0.01,17.02,7.011,15]octadec-6-ene-9-carboxylate	0.891304	-8.0
**153930403**	Eplerenone	ligand_67	methyl (1R,2S,9R,10R,11S,14R,15R,17R)-14-ethyl-2,15-dimethyl-5-oxo-18-oxapentacyclo[8.8.0.01,17.02,7.011,15]octadec-6-ene-9-carboxylate	0.891304	-9.7
**44149679**	Eplerenone	ligand_68	9,11alpha-Epoxy-17-hydroxypregn-4-ene-3,20-dione 17-acetate	0.888889	-5.9
**58700874**	Eplerenone	ligand_69	9,11-Epoxy-17-hydroxy-3-oxo-pregn-4-ene-7,21-dicarboxylic acid, gamma-lactone, (7alpha,11alpha,17alpha)-	0.888889	-8.0
**71316376**	Eplerenone	ligand_70	(9beta,11beta)-Epoxy Fluorometholone Acetate	0.888889	-4.5
**129853378**	Eplerenone	ligand_71	9,11-Epoxy-17-acetyloxypregna-1,4-diene-3,20-dione	0.888889	-5.7
**58755489**	Eplerenone	ligand_72	(1R,2S,9R,14R,15S,17S)-2,15-dimethyl-5,5'-dioxospiro[18-oxapentacyclo[8.8.0.01,17.02,7.011,15]octadec-6-ene-14,2'-oxolane]-9-carbonitrile	0.888889	-5.9
**91172757**	Eplerenone	ligand_73	(1R,2S,9R,14R,15S,17R)-2,15-dimethyl-5,5'-dioxospiro[18-oxapentacyclo[8.8.0.01,17.02,7.011,15]octadec-7-ene-14,2'-oxolane]-9-carbonitrile	0.888889	-3.3
**91479571**	Eplerenone	ligand_74	(1R,2S,14R,15S,17R)-9-butanoyl-2,15-dimethylspiro[18-oxapentacyclo[8.8.0.01,17.02,7.011,15]octadec-8-ene-14,5'-oxolane]-2',5-dione	0.888889	-4.4
**156028069**	Eplerenone	ligand_75	HB8V386D5U	0.883721	-8.6
**91810647**	Eplerenone	ligand_76	21-Hydroxyeplerenone	0.877551	-7.9
**58834237**	Eplerenone	ligand_77	methyl (1R,2S,8R,9R,14R,15S,17S)-8-(hydroxymethyl)-2,15-dimethyl-5,5'-dioxospiro[18-oxapentacyclo[8.8.0.01,17.02,7.011,15]octadec-6-ene-14,2'-oxolane]-9-carboxylate	0.877551	-5.2

**89006738**	Eplerenone	ligand_78	methyl 2-[(1R,9S,14R,17R)-16-hydroxy-2,15-dimethyl-5,5'-dioxospiro[18-oxapentacyclo[8.8.0.01,17.02,7.011,15]octadec-6-ene-14,2'-oxolane]-9-yl]acetate	0.877551	-4.3
**144074798**	Eplerenone	ligand_79	(1R,2S,9R,14R,15S,17R)-9-(methoxymethoxymethyl)-2,15-dimethylspiro[18-oxapentacyclo[8.8.0.01,17.02,7.011,15]octadec-6-ene-14,5'-oxolane]-2',5-dione	0.877551	-5.0
**10498613**	Eplerenone	ligand_80	(1R,2S,4R,10R,11S,14S,15R,18S)-10,14-dimethylspiro[3,8-dioxapentacyclo[9.7.0.02,4.05,10.014,18]octadec-5-ene-15,5'-oxolane]-2',7-dione	0.869565	-8.3
**143696635**	Eplerenone	ligand_81	[(2S,14S,15S,17R)-2,15-dimethyl-5-oxo-18-oxapentacyclo[8.8.0.01,17.02,7.011,15]octadec-6-en-14-yl] propanoate	0.869565	-8.4
**60000064**	Eplerenone	ligand_82	(1S,4S,5S,10S,17S)-4,17-dimethyl-5'-methylidenespiro[18-oxapentacyclo[8.7.2.01,9.04,8.012,17]nonadec-12-ene-5,2'-oxolane]-14,19-dione	0.863636	-3.2
**10713281**	Eplerenone	ligand_83	(1R,2R,7S,10S,11S,14R,15S,17R)-2,15-dimethylspiro[18-oxapentacyclo[8.8.0.01,17.02,7.011,15]octadec-3-ene-14,5'-oxolane]-2',5-dione	0.860465	-7.7
**90980837**	Eplerenone	ligand_84	(1R,9R,14R,17R)-2,15-dimethyl-5,5'-dioxospiro[18-oxapentacyclo[8.8.0.01,17.02,7.011,15]octadec-7-ene-14,2'-oxolane]-9-carboperoxoic acid	0.854167	-4.1
**142945986**	Eplerenone	ligand_85	3-[(1R,2S,15S,17R)-9-methoxycarbonyl-2,14,15-trimethyl-5-oxo-18-oxapentacyclo[8.8.0.01,17.02,7.011,15]octadec-6-en-14-yl]propanoic acid	0.854167	-5.9
**11744281**	Eplerenone	ligand_86	[(1S,2S,4R,10R,11S,14S,15R,18S)-15-acetyl-15-acetyloxy-10,14-dimethyl-7-oxo-3,8-dioxapentacyclo[9.7.0.02,4.05,10.014,18]octadec-5-en-12-yl] acetate	0.854167	-3.9
**57085919**	Eplerenone	ligand_87	[(1R,2S,10S,11S,14R,15S)-14-acetyl-2,8,13,15-tetramethyl-5-oxo-18-oxapentacyclo[8.8.0.01,17.02,7.011,15]octadeca-3,6-dien-14-yl] butanoate	0.851064	-3.1
**88268785**	Eplerenone	ligand_88	(1R,2S,9R,14R,15S,17R)-9-iodo-2,15-dimethylspiro[18-oxapentacyclo[8.8.0.01,17.02,7.011,15]octadec-6-ene-14,5'-oxolane]-2',5-dione	0.851064	-4.0

**88609385**	Eplerenone	ligand_89	[(1R,2S,8S,10S,11S,13S,14R,15S,17S)-14-acetyl-2,8,13,15-tetramethyl-5-oxo-18-oxapentacyclo[8.8.0.01,17.02,7.011,15]octadec-6-en-14-yl] propanoate	0.851064	-3.6
**637221**	Eplerenone	ligand_90	11-A-Hydroxy canrenone methyl ester	0.847826	-5.9
**59086349**	Eplerenone	ligand_91	methyl (11S,15S,17S)-11-hydroxy-10,13-dimethyl-3,5'-dioxospiro[2,6,7,8,9,11,12,14,15,16-decahydro-1H-cyclopenta[a]phenanthrene-17,2'-oxolane]-15-carboxylate	0.847826	-2.6
**57154744**	Eplerenone	ligand_92	[(1R,2S,10S,11S,14R,15S)-14-acetyl-2,8,13,15-tetramethyl-5-oxo-18-oxapentacyclo[8.8.0.01,17.02,7.011,15]octadeca-3,6-dien-14-yl] acetate	0.844444	-5.2
**101731960**	Eplerenone	ligand_93	[(7R,8R,9S,10R,12S,13S,14S,17S)-7-acetyloxy-10,13-dimethyl-17-[(2S)-2-methyl-5-oxooxolan-2-yl]-3-oxo-1,2,6,7,8,9,11,12,14,15,16,17-dodecahydrocyclopenta[a]phenanthren-12-yl] acetate	0.844444	-2.6
**9824823**	Eplerenone	ligand_94	methyl (1R,2S,9R,10R,11S,14R,15S,17R)-14-hydroxy-14-(3-methoxy-3-oxopropyl)-2,15-dimethyl-5-oxo-18-oxapentacyclo[8.8.0.01,17.02,7.011,15]octadec-6-ene-9-carboxylate	0.836735	-5.8
**9887243**	Eplerenone	ligand_95	methyl 3-[(1R,2S,8R,10S,11S,12S,15R,16S,18R)-15-hydroxy-2,16-dimethyl-5-oxo-19-oxahexacyclo[9.8.0.01,18.02,7.08,10.012,16]nonadec-6-en-15-yl]propanoate	0.836735	-6.2
**59059886**	Eplerenone	ligand_96	(1R,9R,14R,17S)-2,15-dimethyl-5,5'-dioxospiro[18-oxapentacyclo[8.8.0.01,17.02,7.011,15]octadec-6-ene-14,2'-oxolane]-9-carboperoxoic acid	0.836735	-0.6
**87503340**	Eplerenone	ligand_97	methyl (1R,3R,5S,6S,7S,9S,10S,11R,12R,19S)-6-hydroxy-6-(3-methoxy-3-oxopropyl)-5,19-dimethyl-16-oxo-2-oxahexacyclo[9.8.0.01,3.05,10.07,9.014,19]nonadec-14-ene-12-carboxylate	0.836735	-6.3
**90724497**	Eplerenone	ligand_98	methyl (1R,2S,9R,10R,11S,14R,15S)-14-hydroxy-14-(3-methoxy-3-oxopropyl)-2,15-dimethyl-5-oxo-18-oxapentacyclo[8.8.0.01,17.02,7.011,15]octadec-7-ene-9-carboxylate	0.836735	-6.7
**58834224**	Eplerenone	ligand_99	(1'R,2'S,4S,15'S,17'S)-2',15'-dimethylspiro[1,3-dioxolane-4,14'-18-oxapentacyclo[8.8.0.01,17.02,7.011,15]octadec-6-ene]-2,5'-dione	0.833333	-4.5

**91502898**	Eplerenone	ligand_100	(1'R,2'S,4S,15'S,17'R)-2',15'-dimethylspiro[1,3-dioxolane-4,14'-18-oxapentacyclo[8.8.0.01,17.02,7.011,15]octadec-7-ene]-2,5'-dione	0.833333	-5.3
**59047543**	Eplerenone	ligand_101	methyl (7R,10R,11R,13S,17R)-11-carbonofluoridoyloxy-10,13-dimethyl-3,5'-dioxospiro[2,6,7,8,9,11,12,14,15,16-decahydro-1H-cyclopenta[a]phenanthrene-17,2'-oxolane]-7-carboxylate	0.829787	-4.3
**91211132**	Eplerenone	ligand_102	methyl (7S,10R,11R,13S,17R)-11-carbonofluoridoyloxy-10,13-dimethyl-3,5'-dioxospiro[2,4,7,8,9,11,12,14,15,16-decahydro-1H-cyclopenta[a]phenanthrene-17,2'-oxolane]-7-carboxylate	0.829787	-3.7
**140492009**	Eplerenone	ligand_103	CID 140492009	0.829787	-2.0
**163614197**	Eplerenone	ligand_104	methyl (7R,9S,10S,11R,13S,17R)-11-hydroxy-9,10,13-trimethyl-3,5'-dioxospiro[1,2,6,7,8,11,12,14,15,16-decahydrocyclopenta[a]phenanthrene-17,2'-oxolane]-7-carboxylate	0.829787	-6.3
**9869347**	Eplerenone	ligand_105	SC-70303 free acid	0.820000	-8.7
**10096854**	Eplerenone	ligand_106	3-[(1R,2S,9R,10R,11R,14R,15S,17R)-14-hydroxy-9-methoxycarbonyl-2,10,15-trimethyl-5-oxo-18-oxapentacyclo[8.8.0.01,17.02,7.011,15]octadec-6-en-14-yl]propanoic acid	0.820000	-5.7
**60120680**	Eplerenone	ligand_107	methyl (1R,2S,9R,10R,11R,14S,15S,17R)-14-hydroxy-2,10,15-trimethyl-5-oxo-14-propyl-18-oxapentacyclo[8.8.0.01,17.02,7.011,15]octadec-6-ene-9-carboxylate	0.820000	-5.8
**87835999**	Eplerenone	ligand_108	methyl 3-[(1R,3R,5S,6S,7S,10S,11S,12S,20S)-6-hydroxy-5,20-dimethyl-17-oxo-2-oxahexacyclo[10.8.0.01,3.05,11.07,10.015,20]icos-15-en-6-yl]propanoate	0.820000	-6.8
**87986620**	Eplerenone	ligand_109	methyl 3-[(1R,2S,10S,11S,14R,15S,17R)-14-hydroxy-2,15-dimethyl-4-methylidene-5-oxo-18-oxapentacyclo[8.8.0.01,17.02,7.011,15]octadec-6-en-14-yl]propanoate	0.820000	-5.6
**90748755**	Eplerenone	ligand_110	3-[(2S,9R,10R,11S,14R,15S,17R)-14-hydroxy-9-methoxycarbonyl-2,15-dimethyl-5-oxo-18-oxapentacyclo[8.8.0.01,17.02,7.011,15]octadec-7-en-14-yl]propanoic acid	0.820000	-8.1

**90815680**	Eplerenone	ligand_111	3-[(2S,9R,10R,11R,14R,15S,17R)-14-hydroxy-9-methoxycarbonyl-2,10,15-trimethyl-5-oxo-18-oxapentacyclo[8.8.0.01,17.02,7.011,15]octadec-7-en-14-yl]propanoic acid	0.820000	-6.7
**91060637**	Eplerenone	ligand_112	methyl (1R,2S,9R,10R,11R,14S,15S,17R)-14-hydroxy-2,10,15-trimethyl-5-oxo-14-propyl-18-oxapentacyclo[8.8.0.01,17.02,7.011,15]octadec-7-ene-9-carboxylate	0.820000	-6.3
**141078426**	Eplerenone	ligand_113	(1R,2S,9R,10R,11S,14S,15S,17R)-14-hydroxy-14-(3-methoxy-3-oxopropyl)-2,15-dimethyl-5-oxo-18-oxapentacyclo[8.8.0.01,17.02,7.011,15]octadec-6-ene-9-carboxylic acid	0.820000	-6.8
**71300503**	Eplerenone	ligand_114	9beta,11beta-Epoxy-17,21-dihydroxy-16beta-methylpregna-1,4-diene-3,20-dione 17,21-di(acetate)	0.816327	-2.2
**88769314**	Eplerenone	ligand_115	[2-[(1S,2S,10S,11S,14R,15S,17S)-14-acetyloxy-2,15-dimethyl-5-oxo-18-oxapentacyclo[8.8.0.01,17.02,7.011,15]octadeca-3,6-dien-14-yl]-2-oxoethyl] acetate	0.816327	-4.1
**57354711**	Eplerenone	ligand_116	3,20-Dioxo-9,11-epoxypregn-4-ene-17,21-diyl diacetate	0.816327	-6.1
**88641996**	Eplerenone	ligand_117	[(1R,2S,8S,10S,11S,13S,14R,15S,17S)-14-acetyl-2,8,13,15-tetramethyl-5-oxo-18-oxapentacyclo[8.8.0.01,17.02,7.011,15]octadeca-3,6-dien-14-yl] propanoate	0.808511	-4.0
**60000063**	Eplerenone	ligand_118	methyl (1R,2S,9R,14R,15S,17S)-14-hydroxy-14-(3-hydroxybut-3-enyl)-2,15-dimethyl-5-oxo-18-oxapentacyclo[8.8.0.01,17.02,7.011,15]octadec-6-ene-9-carboxylate	0.803922	-6.5
**87642137**	Eplerenone	ligand_119	3-[(1S,2S,4R,5R,6R,9S,10S,11R,18R)-6-hydroxy-11-methoxycarbonyl-5,18-dimethyl-15-oxo-3-oxapentacyclo[8.8.0.02,4.05,9.013,18]octadec-13-en-6-yl]propanoic acid	0.803922	-8.1
**160740819**	Eplerenone	ligand_120	methyl (1R,2S,9R,10R,11S,14R,15S)-14-hydroxy-14-(3-methoxy-3-oxopropyl)-2,15-dimethyl-5-oxo-18-oxapentacyclo[8.8.0.01,17.02,7.011,15]octadec-6-ene-9-carboxylate;sulfane	0.803922	-3.2

**10141564**	Eplerenone	ligand_121	3-[(2S,9R,10R,14R,15S,17R)-10-ethenyl-14-hydroxy-9-methoxycarbonyl-2,15-dimethyl-5-oxo-18-oxapentacyclo[8.8.0.01,17.02,7.011,15]octadec-6-en-14-yl]propanoic acid	0.803922	-1.2
**56606679**	Eplerenone	ligand_122	[(2S,8S,10S,11S,13S,14S,15S,17S)-14-(2-acetyloxyacetyl)-2,8,15-trimethyl-5-oxo-18-oxapentacyclo[8.8.0.01,17.02,7.011,15]octadec-6-en-13-yl]methyl propanoate	0.800000	-1.0
**56629320**	Eplerenone	ligand_123	[(2S,8S,10S,11S,13S,14S,15S,17S)-2,8,15-trimethyl-5-oxo-14-(2-propanoyloxyacetyl)-18-oxapentacyclo[8.8.0.01,17.02,7.011,15]octadec-6-en-13-yl]methyl propanoate	0.800000	-1.0
**58653070**	Eplerenone	ligand_124	(1S,14R,17S)-2,15-dimethyl-5-oxo-14-propanoyloxy-18-oxapentacyclo[8.8.0.01,17.02,7.011,15]octadeca-3,6-diene-14-carbothioate	0.800000	-4.4
**88609376**	Eplerenone	ligand_125	[(2S,8S,10S,11S,13S,14R,15S,17S)-14-(2-acetyloxyacetyl)-2,8,13,15-tetramethyl-5-oxo-18-oxapentacyclo[8.8.0.01,17.02,7.011,15]octadec-6-en-14-yl] propanoate	0.800000	-0.3
**88609379**	Eplerenone	ligand_126	[2-oxo-2-[(1R,2S,8S,10S,11S,13S,14R,15S,17S)-2,8,13,15-tetramethyl-5-oxo-14-propanoyloxy-18-oxapentacyclo[8.8.0.01,17.02,7.011,15]octadec-6-en-14-yl]ethyl] propanoate	0.800000	0.0
**88680965**	Eplerenone	ligand_127	[(1S,2S,10S,11S,13R,14S,15S,17S)-2,13,15-trimethyl-5-oxo-14-(2-propanoyloxyacetyl)-18-oxapentacyclo[8.8.0.01,17.02,7.011,15]octadeca-3,6-dien-14-yl] pentanoate	0.800000	-0.8
**139024642**	Eplerenone	ligand_128	[2-oxo-2-[(1S,2S,10S,11R,13S,15S,17S)-2,13,15-trimethyl-5-oxo-14-propanoyloxy-18-oxapentacyclo[8.8.0.01,17.02,7.011,15]octadec-6-en-14-yl]ethyl] propanoate	0.800000	-1.2
**68133595**	Eplerenone	ligand_129	[(8R,9S,10R,12R,13S,14S,15S,17S)-17-acetyl-12-acetyloxy-10,13-dimethyl-3-oxo-1,2,6,7,8,9,11,12,14,15,16,17-dodecahydrocyclopenta[a]phenanthren-15-yl] acetate	0.795455	-4.8
**60038044**	Eplerenone	ligand_130	(9R,17R)-9-ethoxy-10,13-dimethylspiro[1,2,6,7,8,11,12,14,15,16-decahydrocyclopenta[a]phenanthrene-17,5'-oxolane]-2',3-dione	0.791667	-4.5

**90936829**	Eplerenone	ligand_131	(9R,17R)-9-ethoxy-10,13-dimethylspiro[1,2,4,7,8,11,12,14,15,16-decahydrocyclopenta[a]phenanthrene-17,5'-oxolane]-2',3-dione	0.791667	-5.8
**88695588**	Eplerenone	ligand_132	ethyl (1R,2S,9R,10R,11S,14S,15S,17R)-14-hydroxy-14-(3-methoxy-3-oxopropyl)-2,15-dimethyl-5-oxo-18-oxapentacyclo[8.8.0.01,17.02,7.011,15]octadec-6-ene-9-carboxylate	0.788462	-7.3
**57301873**	Eplerenone	ligand_133	[(1R,2S,10S,11S,14R,15S)-14-acetyl-8-fluoro-2,15-dimethyl-5-oxo-18-oxapentacyclo[8.8.0.01,17.02,7.011,15]octadeca-3,6-dien-14-yl] butanoate	0.784314	-5.7
**88762280**	Eplerenone	ligand_134	[(1S,2S,8S,10S,11S,14R,15S,17S)-14-acetyl-8-fluoro-2,15-dimethyl-5-oxo-18-oxapentacyclo[8.8.0.01,17.02,7.011,15]octadeca-3,6-dien-14-yl] propanoate	0.784314	-4.0
**9913840**	Eplerenone	ligand_135	3-[(1R,2S,9R,10R,11S,14R,15S,17R)-14-hydroxy-2,15-dimethyl-5-oxo-9-propan-2-yloxycarbonyl-18-oxapentacyclo[8.8.0.01,17.02,7.011,15]octadec-6-en-14-yl]propanoic acid	0.784314	-7.8
**58606553**	Eplerenone	ligand_136	3-[(2S,10R,11R,14R,15S,17R)-9-acetyl-14-hydroxy-2,10,15-trimethyl-5-oxo-18-oxapentacyclo[8.8.0.01,17.02,7.011,15]octadec-6-en-14-yl]propanoic acid	0.780000	-5.9
**59112026**	Eplerenone	ligand_137	3-[(2S,9R,10R,11S,14R,15S,17R)-9-acetyl-14-hydroxy-2,15-dimethyl-5-oxo-18-oxapentacyclo[8.8.0.01,17.02,7.011,15]octadec-6-en-14-yl]propanoic acid	0.780000	-9.1
**91449297**	Eplerenone	ligand_138	(1R,2S,10R,11S,14S,15S)-14-(2-carboxyethyl)-14-hydroxy-2,9,15-trimethyl-5-oxo-18-oxapentacyclo[8.8.0.01,17.02,7.011,15]octadec-6-ene-9-carboxylic acid	0.780000	-5.6
**131699376**	Eplerenone	ligand_139	(2S,14S,15S,17S)-14-(3-carboxypropyl)-14-hydroxy-2,9,15-trimethyl-5-oxo-18-oxapentacyclo[8.8.0.01,17.02,7.011,15]octadec-6-ene-9-carboxylic acid	0.780000	-4.9
**87871063**	Eplerenone	ligand_140	[(1S,2S,8S,10S,11S,13S,14R,15S,17S)-14-acetyl-8-fluoro-2,13,15-trimethyl-5-oxo-18-oxapentacyclo[8.8.0.01,17.02,7.011,15]octadeca-3,6-dien-14-yl] acetate	0.775510	-2.0
**60077468**	Eplerenone	ligand_141	(2S,9R,10R,11S,14R,15S,17R)-9-acetyl-14-hydroxy-2,15-dimethyl-14-(3-oxobutyl)-18-oxapentacyclo[8.8.0.01,17.02,7.011,15]octadec-6-en-5-one	0.775510	-8.3

**87759522**	Eplerenone	ligand_142	methyl (1R,2S,9R,10R,11S,14R,15S,17R)-14-hydroxy-14-(3-hydroxypropyl)-2,15-dimethyl-5-oxo-18-oxapentacyclo[8.8.0.01,17.02,7.011,15]octadec-6-ene-9-carboxylate	0.773585	-8.6
**171119316**	Eplerenone	ligand_143	Arthriniumsteroid C	0.769231	-1.9
**57045943**	Eplerenone	ligand_144	[2-[(1S,2S,10S,11S,14R,15S,17S)-14-acetyloxy-2,15-dimethyl-5-oxo-18-oxapentacyclo[8.8.0.01,17.02,7.011,15]octadec-6-en-14-yl]-2-oxoethyl] 2-fluoropropanoate	0.769231	-5.5
**168376990**	Eplerenone	ligand_145	9,11-Epoxy-17-hydroxypregn-4-ene-3,20-dione actate	0.769231	-4.7
**141869406**	Eplerenone	ligand_146	3-[(1S,2S,5R,6R,9S,10S,11R,18R)-6-hydroxy-5,18-dimethyl-15-oxo-11-propan-2-yloxycarbonyl-3-oxapentacyclo[8.8.0.02,4.05,9.013,18]octadec-13-en-6-yl]propanoic acid	0.769231	-7.6
**10075515**	Eplerenone	ligand_147	3-[(1R,2S,9R,10R,11R,14R,15S,17R)-10-ethenyl-14-hydroxy-2,15-dimethyl-5-oxo-9-propan-2-yloxycarbonyl-18-oxapentacyclo[8.8.0.01,17.02,7.011,15]octadec-6-en-14-yl]propanoic acid	0.769231	-5.5
**60132590**	Eplerenone	ligand_148	(7S,8S,10R,11R,13S,14S)-7,11-dihydroxy-10,13-dimethylspiro[2,6,7,8,9,11,12,14,15,16-decahydro-1H-cyclopenta[a]phenanthrene-17,5'-oxolane]-2',3-dione	0.765957	-4.0
**21044284**	Eplerenone	ligand_149	4-[(1S,2S,10S,11S,13S,14R,15S,17S)-14-hydroxy-2,13,15-trimethyl-5-oxo-18-oxapentacyclo[8.8.0.01,17.02,7.011,15]octadeca-3,6-dien-14-yl]-4-oxobutanoic acid	0.764706	-4.4
**59116724**	Eplerenone	ligand_150	3-[(1R,2S,9R,10R,11R,14R,15S,17R)-9-acetyl-10-ethenyl-14-hydroxy-2,15-dimethyl-5-oxo-18-oxapentacyclo[8.8.0.01,17.02,7.011,15]octadec-6-en-14-yl]propanoic acid	0.764706	-5.2
**87852688**	Eplerenone	ligand_151	(2S,10R,11S,14S,15S)-14-(2-carboxyethyl)-4-ethyl-14-hydroxy-2,9,15-trimethyl-5-oxo-18-oxapentacyclo[8.8.0.01,17.02,7.011,15]octadec-6-ene-9-carboxylic acid	0.764706	-1.2
**87986238**	Eplerenone	ligand_152	3-[(1R,2S,10S,11S,14R,15S)-8,9,12,13-tetraethyl-14-hydroxy-2,15-dimethyl-5-oxo-18-oxapentacyclo[8.8.0.01,17.02,7.011,15]octadec-6-en-14-yl]propanoic acid	0.764706	-1.4

**89598941**	Eplerenone	ligand_153	(2S,10R,11S,14S,15S)-14-(2-carboxyethyl)-14-hydroxy-2,15-dimethyl-5-oxo-9-propan-2-yl-18-oxapentacyclo[8.8.0.01,17.02,7.011,15]octadec-6-ene-9-carboxylic acid	0.764706	-5.3
**90939096**	Eplerenone	ligand_154	3-[(1R,2S,9R,10R,11R,14R,15S,17R)-9-acetyl-14-hydroxy-2,10,15-trimethyl-5-oxo-18-oxapentacyclo[8.8.0.01,17.02,7.011,15]octadec-6-en-14-yl]propanoic acid;ethene	0.764706	-5.7
**9800350**	Eplerenone	ligand_155	3-[(1R,2S,8R,10S,11S,12S,15R,16S,18R)-15-hydroxy-2,16-dimethyl-5-oxo-19-oxahexacyclo[9.8.0.01,18.02,7.08,10.012,16]nonadec-6-en-15-yl]propanoic acid	0.760000	-8.4
**21018225**	Eplerenone	ligand_156	(2S,10R,11S,14S,15S)-14-(2-carboxyethyl)-14-hydroxy-2,15-dimethyl-5-oxo-18-oxapentacyclo[8.8.0.01,17.02,7.011,15]octadec-6-ene-9-carboxylic acid	0.760000	-8.6
**53733026**	Eplerenone	ligand_157	3-[(2S,10S,11S,14S,15S,17R)-14-hydroxy-2,15-dimethyl-5-oxo-18-oxapentacyclo[8.8.0.01,17.02,7.011,15]octadeca-6,8-dien-14-yl]propanoic acid	0.760000	-7.8
**88670023**	Eplerenone	ligand_158	[2-oxo-2-[(1R,2S,8S,10S,11S,13S,14R,15S,17S)-2,8,13,15-tetramethyl-5-oxo-14-propanoyloxy-18-oxapentacyclo[8.8.0.01,17.02,7.011,15]octadeca-3,6-dien-14-yl]ethyl] propanoate	0.760000	0.5
**91126437**	Eplerenone	ligand_159	(1R,2S,10R,11S,14S,15S)-14-(2-carboxyethyl)-14-hydroxy-2,15-dimethyl-18-oxapentacyclo[8.8.0.01,17.02,7.011,15]octadec-6-ene-9-carboxylic acid	0.760000	-8.7
**154305911**	Eplerenone	ligand_160	(1R,2S,10R,11S,14S,15S)-14-(2-carboxylatoethyl)-14-hydroxy-2,15-dimethyl-5-oxo-18-oxapentacyclo[8.8.0.01,17.02,7.011,15]octadec-6-ene-9-carboxylate	0.760000	-6.0
**88694605**	Eplerenone	ligand_161	Pregna-1,4-diene-3,20-dione, 17,21-bis(acetyloxy)-9,11-epoxy-6-fluoro-, (6alpha,9beta,11beta)-	0.754717	-3.9
**88654681**	Eplerenone	ligand_162	ZH29Mqq4EB	0.754717	-5.7
**88762947**	Eplerenone	ligand_163	[2-[(1S,2S,8S,10S,11S,13S,14R,15S,17S)-14-acetyloxy-8-fluoro-2,13,15-trimethyl-5-oxo-18-oxapentacyclo[8.8.0.01,17.02,7.011,15]octadec-6-en-14-yl]-2-oxoethyl] acetate	0.754717	-1.2
**86737548**	Eplerenone	ligand_164	9beta,11beta-Epoxy-6alpha-fluoro-17alpha,21-diacetoxy-4-pregnen-3,20-dione	0.754717	-4.7

**88761141**	Eplerenone	ligand_165	[2-[(1S,2S,10S,11S,13R,14R,15S,17S)-14-acetyloxy-13-fluoro-2,13,15-trimethyl-5-oxo-18-oxapentacyclo[8.8.0.01,17.02,7.011,15]octadec-6-en-14-yl]-2-oxoethyl] acetate	0.754717	-1.8
**46892694**	Eplerenone	ligand_166	[3-[(2S,8S,14S,15S,17R)-14-acetyloxy-8-fluoro-2,15-dimethyl-5-oxo-18-oxapentacyclo[8.8.0.01,17.02,7.011,15]octadec-6-en-14-yl]-2-oxopropyl] acetate	0.754717	-5.6
**57220910**	Eplerenone	ligand_167	3-[(1R,2S,10R,11S,14S,15S)-9-ethoxycarbonyl-14-hydroxy-2,15-dimethyl-5-oxo-18-oxapentacyclo[8.8.0.01,17.02,7.011,15]octadec-6-en-14-yl]propanoic acid	0.754717	-8.9
**24764663**	Eplerenone	ligand_168	3-[(1R,2S,9R,10R,11S,14R,15S,17R)-9-butoxycarbonyl-14-hydroxy-2,15-dimethyl-5-oxo-18-oxapentacyclo[8.8.0.01,17.02,7.011,15]octadec-6-en-14-yl]propanoic acid	0.754717	-7.9
**220509**	Eplerenone	ligand_169	9,11-Epoxy-17-hydroxy-17-methylandrost-4-en-3-one, (9beta,11beta,17beta)-	0.750000	-7.0
**235985**	Eplerenone	ligand_170	17-Hydroxy-2,17-dimethyl-9,11-epoxyandrost-4-en-3-one	0.750000	-5.9
**59086365**	Eplerenone	ligand_171	(7R,10R,11R,13S,17R)-11-hydroxy-10,13-dimethyl-3,5'-dioxospiro[2,6,7,8,9,11,12,14,15,16-decahydro-1H-cyclopenta[a]phenanthrene-17,2'-oxolane]-7-carboxylic acid	0.750000	-6.4
**91519757**	Eplerenone	ligand_172	(2S,10S,11S,14R,15S)-14-ethyl-14-hydroxy-2,15-dimethyl-5-oxo-18-oxapentacyclo[8.8.0.01,17.02,7.011,15]octadec-6-ene-3,4-dicarboxylic acid	0.750000	-4.4
**88123551**	Eplerenone	ligand_173	methyl (1R,2S,9R,10R,11S,14R,15S,17R)-14-(2-carboxyoxyethyl)-14-hydroxy-2,15-dimethyl-5-oxo-18-oxapentacyclo[8.8.0.01,17.02,7.011,15]octadec-6-ene-9-carboxylate	0.745455	-7.0
**18600756**	Eplerenone	ligand_174	3-[(1R,3R,5S,6S,7S,10S,11S,12S,20S)-6-hydroxy-5,20-dimethyl-17-oxo-2-oxahexacyclo[10.8.0.01,3.05,11.07,10.015,20]icos-15-en-6-yl]propanoic acid	0.745098	-6.9
**57237604**	Eplerenone	ligand_175	3-[(1R,2S,10R,11S,14S,15S)-14-hydroxy-2,15-dimethyl-8,9-dimethylidene-5-oxo-18-oxapentacyclo[8.8.0.01,17.02,7.011,15]octadec-6-en-14-yl]propanoic acid	0.745098	-8.5

**57297275**	Eplerenone	ligand_176	(1S,2S,4R,5R,6R,9S,10S,11R,18R)-6-(2-carboxyethyl)-6-hydroxy-5,18-dimethyl-15-oxo-3-oxapentacyclo[8.8.0.02,4.05,9.013,18]octadec-13-ene-11-carboxylic acid	0.745098	-7.9
**60120676**	Eplerenone	ligand_177	(1R,2S,9R,10R,11R,14S,15S,17R)-9-acetyl-10-ethenyl-14-hydroxy-2,15-dimethyl-14-propyl-18-oxapentacyclo[8.8.0.01,17.02,7.011,15]octadec-6-en-5-one	0.745098	-5.4
**57187274**	Eplerenone	ligand_178	[2-[(1S,2S,8S,10S,11S,14R,15S,17S)-14-acetyloxy-8-fluoro-2,15-dimethyl-5-oxo-18-oxapentacyclo[8.8.0.01,17.02,7.011,15]octadeca-3,6-dien-14-yl]-2-oxoethyl] propanoate	0.740741	-4.0
**57319394**	Eplerenone	ligand_179	[(1R,2S,10S,11S,14R,15S)-14-(2-acetyloxyacetyl)-8-fluoro-2,15-dimethyl-5-oxo-18-oxapentacyclo[8.8.0.01,17.02,7.011,15]octadeca-3,6-dien-14-yl] propanoate	0.740741	-6.5
**156597030**	Eplerenone	ligand_180	(4aS,6aS,7R,9aS,9bS,11S)-7-(2-acetoxyacetyl)-11-fluoro-4a,6a-dimethyl-2-oxo-2,3,4,4a,5a,6,6a,7,8,9,9a,9b,10,11-tetradecahydrocyclopenta[1,2]phenanthro[4,4a-b]oxiren-7-yl butyrate	0.740741	-6.1
**57234171**	Eplerenone	ligand_181	(2S,10S,11S,14S,15S,17R)-14-ethyl-14-hydroxy-2,15-dimethyl-18-oxapentacyclo[8.8.0.01,17.02,7.011,15]octadec-6-en-5-one	0.720000	-8.7
**13051552**	Eplerenone	ligand_182	[2-[(1S,2S,8S,10S,11S,13R,14R,15S,17S)-14-acetyloxy-8-fluoro-2,13,15-trimethyl-5-oxo-18-oxapentacyclo[8.8.0.01,17.02,7.011,15]octadeca-3,6-dien-14-yl]-2-oxoethyl] acetate	0.716981	-2.5
**23520050**	Eplerenone	ligand_183	2-[(1S,2S,8S,10S,11S,14R,15S,17S)-14-acetyl-3-(carboxymethyl)-8-fluoro-14-hydroxy-2,15-dimethyl-5-oxo-18-oxapentacyclo[8.8.0.01,17.02,7.011,15]octadeca-3,6-dien-4-yl]acetic acid	0.709091	-2.1
**88124090**	Eplerenone	ligand_184	ethyl (1R,2S,9R,10R,11S,14R,15S,17R)-14-(2-carboxyoxyethyl)-14-hydroxy-2,15-dimethyl-5-oxo-18-oxapentacyclo[8.8.0.01,17.02,7.011,15]octadec-6-ene-9-carboxylate	0.701754	-7.3
**10523458**	Eplerenone	ligand_185	3-[(1R,2R,7S,10S,11S,14R,15S,17R)-14-hydroxy-2,15-dimethyl-5-oxo-18-oxapentacyclo[8.8.0.01,17.02,7.011,15]octadec-3-en-14-yl]propanoic acid	0.700000	-8.1

**23257016**	Eplerenone	ligand_186	3-[(1R,2R,7S,10S,11S,14R,15S,17R)-14-hydroxy-2,15-dimethyl-5-oxo-18-oxapentacyclo[8.8.0.01,17.02,7.011,15]octadec-3-en-14-yl]propanoate	0.700000	-7.9
**88694778**	Eplerenone	ligand_187	(1R,2S,10S,11S,14R,15S)-13-hydroxy-14-(2-hydroxyethyl)-2,15-dimethyl-18-oxapentacyclo[8.8.0.01,17.02,7.011,15]octadec-6-ene-4,5-dione	0.685185	-9.3
**13789**	Canrenone	ligand_1	Canrenone	1.000000	-8.1
**13061887**	Canrenone	ligand_2	Drospirenone 6-ene	0.972222	-6.5
**16627216**	Canrenone	ligand_3	(10R,13S,17R)-10,13-Dimethyl-1,2,7,8,9,10,11,12,13,14,15,16-dodecahydro-5'H-spiro[cyclopenta[a]phenanthrene-17,2'-oxolane]-3,5',6-trione	0.972222	-6.4
**58882769**	Canrenone	ligand_4	(7R,8R,10S,13S,14S,17R)-10,13-dimethyl-3,5'-dioxospiro[2,6,7,8,12,14,15,16-octahydro-1H-cyclopenta[a]phenanthrene-17,2'-oxolane]-7-carbaldehyde	0.972222	-7.2
**16128534**	Canrenone	ligand_5	(7R,10S,13S,17R)-7,10,13-trimethylspiro[2,6,7,8,12,14,15,16-octahydro-1H-cyclopenta[a]phenanthrene-17,5'-oxolane]-2',3-dione	0.972222	-6.3
**46241505**	Canrenone	ligand_6	CID 46241505	0.972222	-6.0
**46241603**	Canrenone	ligand_7	CID 46241603	0.972222	-6.8
**46241607**	Canrenone	ligand_8	CID 46241607	0.972222	-7.2
**58284139**	Canrenone	ligand_9	(3'S,5S,5'S,7'S,11'R)-7',11'-dimethylspiro[oxolane-5,6'-pentacyclo[8.8.0.02,7.03,5.011,16]octadeca-9,15,17-triene]-2,14'-dione	0.972222	-3.4
**58755488**	Canrenone	ligand_10	(7R,10R,13S,17R)-7,10,13-trimethylspiro[2,6,7,8,9,11,12,14,15,16-decahydro-1H-cyclopenta[a]phenanthrene-17,5'-oxolane]-2',3-dione	0.972222	-6.4
**59075897**	Canrenone	ligand_11	(7R,10R,13S)-7,10,11,13-tetramethylspiro[2,6,7,8,9,11,12,14,15,16-decahydro-1H-cyclopenta[a]phenanthrene-17,5'-oxolane]-2',3-dione	0.972222	-4.3
**59936884**	Canrenone	ligand_12	CID 59936884	0.972222	-6.3
**59936887**	Canrenone	ligand_13	CID 59936887	0.972222	-5.5
**68391161**	Canrenone	ligand_14	(8R,9S,10R,13S,14S,17R)-10,13-dimethyl-7-methylidenespiro[1,2,6,8,9,11,12,14,15,16-decahydrocyclopenta[a]phenanthrene-17,5'-oxolane]-2',3-dione	0.972222	-7.8

**70838861**	Canrenone	ligand_15	(10R,13S,17S)-6,10,13,16-tetramethylspiro[2,6,7,8,9,11,12,14,15,16-decahydro-1H-cyclopenta[a]phenanthrene-17,5'-oxolane]-2',3-dione	0.972222	-3.5
**91373883**	Canrenone	ligand_16	(8S,10R,13S,14S,17R)-10,13-dimethylspiro[1,2,8,9,12,14,15,16-octahydrocyclopenta[a]phenanthrene-17,5'-oxolane]-2',3,11-trione	0.972222	-5.6
**122680936**	Canrenone	ligand_17	(3S,8R,9S,10R,13S,14S,17R)-3,10,13-trimethylspiro[2,3,4,8,9,11,12,14,15,16-decahydro-1H-cyclopenta[a]phenanthrene-17,5'-oxolane]-2',7-dione	0.972222	-6.5
**138625441**	Canrenone	ligand_18	(8R,9S,10R,13S,14S,17S)-10,13,15-trimethylspiro[2,6,7,8,9,11,12,14,15,16-decahydro-1H-cyclopenta[a]phenanthrene-17,5'-oxolane]-2',3-dione	0.972222	-5.9
**143827924**	Canrenone	ligand_19	CID 143827924	0.972222	-4.6
**143827935**	Canrenone	ligand_20	CID 143827935	0.972222	-4.9
**144011953**	Canrenone	ligand_21	CID 144011953	0.972222	-6.8
**163853644**	Canrenone	ligand_22	(3'S,5R,7'S,11'R)-7',11'-dimethylspiro[oxolane-5,6'-pentacyclo[8.8.0.02,7.03,5.011,16]octadeca-4,15,17-triene]-2,14'-dione	0.972222	-4.2
**13315007**	Canrenone	ligand_23	(1'R,2'S,3'S,5S,5'S,7'S,10'S,11'R)-7',11'-dimethylspiro[oxolane-5,6'-pentacyclo[8.8.0.02,7.03,5.011,16]octadeca-12,15,17-triene]-2,14'-dione	0.972222	-6.6
**95560774**	Canrenone	ligand_24	(2'R,8S,9S,10R,13S,14S)-10,13-dimethyl-1,7,8,9,10,12,13,14,15,16-decahydro-3'H-spiro[cyclopenta[a]phenanthrene-17,2'-furan]-3,5',11(2H,4'H,6H)-trione	0.972222	-6.0
**169447566**	Canrenone	ligand_25	(2'S,8R,9S,10R,13S,14S,15R)-10,13,15-Trimethyl-1,8,9,10,11,12,13,14,15,16-decahydro-3'H-spiro[cyclopenta[a]phenanthrene-17,2'-furan]-3,5'(2H,4'H)-dione	0.972222	-5.7
**58115334**	Canrenone	ligand_26	(8R,9S,10R,13S,14S,17R)-13-methylspiro[1,2,8,9,10,11,12,14,15,16-decahydrocyclopenta[a]phenanthrene-17,5'-furan]-2',3-dione	0.971429	-8.6
**44290097**	Canrenone	ligand_27	(17R)-10,13-dimethylspiro[9,14,15,16-tetrahydro-8H-cyclopenta[a]phenanthrene-17,5'-oxolane]-2',3-dione	0.971429	-6.1
**44290180**	Canrenone	ligand_28	(17R)-10,13-dimethylspiro[7,8,9,14,15,16-hexahydro-6H-cyclopenta[a]phenanthrene-17,5'-oxolane]-2',3-dione	0.971429	-6.8
**44304798**	Canrenone	ligand_29	(13S,17R)-13-methylspiro[2,6,7,8,9,11,12,14,15,16-decahydrocyclopenta[a]phenanthrene-17,5'-oxolane]-2',3-dione	0.971429	-7.3

**168943**	Canrenone	ligand_30	(8R,9S,10R,13S,14S,17R)-13-methylspiro[1,2,6,7,8,9,10,11,12,14,15,16-dodecahydrocyclopenta[a]phenanthrene-17,5'-oxolane]-2',3-dione	0.971429	-7.8
**44317101**	Canrenone	ligand_31	(13S,17R)-13-methylspiro[1,2,6,7,8,11,12,14,15,16-decahydrocyclopenta[a]phenanthrene-17,5'-oxolane]-2',3-dione	0.971429	-7.1
**70805057**	Canrenone	ligand_32	(2'R,8R,9S,10R,13S,14S)-13-methyl-1,8,9,10,11,12,13,14,15,16-decahydro-3'H-spiro[cyclopenta[a]phenanthrene-17,2'-furan]-3,5'(2H,4'H)-dione	0.971429	-7.6
**123404869**	Canrenone	ligand_33	(8R,9S,13S,14S,17R)-13-methylspiro[1,2,4,8,9,11,12,14,15,16-decahydrocyclopenta[a]phenanthrene-17,5'-furan]-2',3-dione	0.971429	-9.9
**250780**	Canrenone	ligand_34	3a-Methylspiro[1,2,4,5,8,9,9a,9b-octahydrocyclopenta[a]naphthalene-3,5'-oxolane]-2',7-dione	0.971429	-8.9
**101687283**	Canrenone	ligand_35	(8R,9S,10R,13S,14S,17R)-13-methylspiro[6,7,8,9,10,11,12,14,15,16-decahydrocyclopenta[a]phenanthrene-17,5'-oxolane]-2',3-dione	0.971429	-8.1
**122293**	Canrenone	ligand_36	7-Propyl spirolactone	0.945946	-8.3
**170819**	Canrenone	ligand_37	Prorenone	0.945946	-8.3
**11568294**	Canrenone	ligand_38	5Plg485A5R	0.945946	-8.3
**58700862**	Canrenone	ligand_39	8PJ9GK7FY9	0.945946	-7.6
**90674702**	Canrenone	ligand_40	(8R,9S,10R,13S,14S,17S)-10,13-dimethylspiro[1,2,8,9,11,12,14,16-octahydrocyclopenta[a]phenanthrene-17,5'-oxolane]-2',3,15-trione	0.945946	-7.3
**10157726**	Canrenone	ligand_41	(6R,7R,10R,13S,15S,16S,17S)-6,7,10,13,15,16-hexamethylspiro[2,6,7,8,9,11,12,14,15,16-decahydro-1H-cyclopenta[a]phenanthrene-17,5'-oxolane]-2',3-dione	0.945946	-1.3
**16128453**	Canrenone	ligand_42	(10S,13S,17R)-3',10,13-trimethylspiro[2,6,7,8,12,14,15,16-octahydro-1H-cyclopenta[a]phenanthrene-17,5'-oxolane]-2',3-dione	0.945946	-3.4
**58554599**	Canrenone	ligand_43	(3'S,8R,9S,10R,13S,14S,17R)-3',10,13-trimethylspiro[2,4,7,8,9,11,12,14,15,16-decahydro-1H-cyclopenta[a]phenanthrene-17,5'-oxolane]-2',3-dione	0.945946	-6.9
**58619929**	Canrenone	ligand_44	(8R,9S,10R,13S,14S,17R)-7-acetyl-10,13-dimethylspiro[2,6,7,8,9,11,12,14,15,16-decahydro-1H-cyclopenta[a]phenanthrene-17,5'-oxolane]-2',3-dione	0.945946	-7.9

**58700866**	Canrenone	ligand_45	(7R,8S,10S,13S,14S,17R)-10,13-dimethyl-7-prop-2-enylspiro[2,6,7,8,12,14,15,16-octahydro-1H-cyclopenta[a]phenanthrene-17,5'-oxolane]-2',3-dione	0.945946	-7.9
**58834276**	Canrenone	ligand_46	(7R,10S,13S,17R)-7-but-3-enyl-10,13-dimethylspiro[2,6,7,8,12,14,15,16-octahydro-1H-cyclopenta[a]phenanthrene-17,5'-oxolane]-2',3-dione	0.945946	-7.0
**59075900**	Canrenone	ligand_47	(9S,17R)-9,10,13-trimethylspiro[1,2,6,7,8,11,12,14,15,16-decahydrocyclopenta[a]phenanthrene-17,5'-oxolane]-2',3-dione	0.945946	-5.6
**59075901**	Canrenone	ligand_48	(9S,17R)-9,10,13-trimethylspiro[1,2,8,11,12,14,15,16-octahydrocyclopenta[a]phenanthrene-17,5'-oxolane]-2',3-dione	0.945946	-6.6
**59106598**	Canrenone	ligand_49	CID 59106598	0.945946	91.6
**70351085**	Canrenone	ligand_50	CID 70351085	0.945946	-3.9
**70351277**	Canrenone	ligand_51	CID 70351277	0.945946	-4.3
**70351587**	Canrenone	ligand_52	CID 70351587	0.945946	-5.2
**70496983**	Canrenone	ligand_53	(3'S,5R,5'S,7'S,11'R)-7',11',17'-trimethylspiro[oxolane-5,6'-pentacyclo[8.8.0.02,7.03,5.011,16]octadeca-15,17-diene]-2,14'-dione	0.945946	-3.9
**70805104**	Canrenone	ligand_54	(10R,13S,17R)-13-ethyl-7-methylspiro[1,2,6,7,8,9,10,11,12,14,15,16-dodecahydrocyclopenta[a]phenanthrene-17,5'-oxolane]-2',3-dione	0.945946	-8.2
**70805203**	Canrenone	ligand_55	(10R,13S,17R)-7-ethyl-13-methylspiro[1,2,6,7,8,9,10,11,12,14,15,16-dodecahydrocyclopenta[a]phenanthrene-17,5'-oxolane]-2',3-dione	0.945946	-7.2
**70843444**	Canrenone	ligand_56	(10R,13S,15S,17S)-6,10,13,15,16-pentamethylspiro[2,6,7,8,9,11,12,14,15,16-decahydro-1H-cyclopenta[a]phenanthrene-17,5'-oxolane]-2',3-dione	0.945946	-5.9
**70882513**	Canrenone	ligand_57	CID 70882513	0.945946	-3.5
**70882516**	Canrenone	ligand_58	CID 70882516	0.945946	-3.4
**70882560**	Canrenone	ligand_59	CID 70882560	0.945946	-2.8
**71072622**	Canrenone	ligand_60	(7R,8R,13S,14S,17R)-10,13-dimethyl-7-[(Z)-4-oxopent-2-enoyl]spiro[2,6,7,8,9,11,12,14,15,16-decahydro-1H-cyclopenta[a]phenanthrene-17,5'-oxolane]-2',3-dione	0.945946	-9.6

**71163203**	Canrenone	ligand_61	(7R,17R)-7-acetyl-10,13-dimethylspiro[2,6,7,8,12,14,15,16-octahydro-1H-cyclopenta[a]phenanthrene-17,5'-oxolane]-2',3-dione	0.945946	-3.4
**89555383**	Canrenone	ligand_62	(8R,10R,13S,17R)-13-ethyl-10-methylspiro[2,8,9,11,12,14,15,16-octahydro-1H-cyclopenta[a]phenanthrene-17,5'-oxolane]-2',3-dione	0.945946	-5.5
**89589628**	Canrenone	ligand_63	(8R,9S,10R,13S,14S,15S,16S,17S)-10,13,15,16-tetramethylspiro[2,8,9,11,12,14,15,16-octahydro-1H-cyclopenta[a]phenanthrene-17,5'-oxolane]-2',3-dione	0.945946	-4.2
**89589822**	Canrenone	ligand_64	(8S,9S,10R,13S,14S,15S,16S,17S)-9,10,13,15,16-pentamethylspiro[1,2,8,11,12,14,15,16-octahydrocyclopenta[a]phenanthrene-17,5'-oxolane]-2',3-dione	0.945946	-4.2
**89605713**	Canrenone	ligand_65	1,10,13-trimethylspiro[2,6,7,8,9,11,12,14,15,16-decahydro-1H-cyclopenta[a]phenanthrene-17,5'-oxolane]-2',3-dione	0.945946	-3.2
**91220343**	Canrenone	ligand_66	CID 91220343	0.945946	-4.0
**142894708**	Canrenone	ligand_67	(1'S,17'R)-2',15'-dimethylspiro[oxolane-5,14'-pentacyclo[8.8.0.01,17.02,7.011,15]octadec-6-ene]-2,5'-dione	0.945946	-6.1
**144011949**	Canrenone	ligand_68	CID 144011949	0.945946	-2.4
**160233617**	Canrenone	ligand_69	(7R,8R,10S,13S,14S,17R)-10,13-dimethyl-7-[(Z)-4-oxopent-2-enoyl]spiro[2,6,7,8,12,14,15,16-octahydro-1H-cyclopenta[a]phenanthrene-17,5'-oxolane]-2',3-dione;methane	0.945946	-7.1
**160706199**	Canrenone	ligand_70	methane;(3'S,8R,9S,10R,13S,14S,17R)-3',10,13-trimethylspiro[2,4,7,8,9,11,12,14,15,16-decahydro-1H-cyclopenta[a]phenanthrene-17,5'-oxolane]-2',3-dione	0.945946	-5.7
**163963516**	Canrenone	ligand_71	(5S,5'S,7'S,11'R,18'R)-7',11',18'-trimethylspiro[oxolane-5,6'-pentacyclo[8.8.0.02,7.03,5.011,16]octadeca-3,15-diene]-2,14'-dione	0.945946	-8.5
**9998146**	Canrenone	ligand_72	(8R,9S,10R,13S,14S,17R)-10,13-dimethyl-2-methylidenespiro[1,8,9,11,12,14,15,16-octahydrocyclopenta[a]phenanthrene-17,5'-oxolane]-2',3-dione	0.945946	-4.7

**137234718**	Canrenone	ligand_73	(6R,7S,8R,10R,13S,16S,17S)-6,7,8,10,13,15,16-heptamethylspiro[1,2,6,7,9,11,12,14,15,16-decahydrocyclopenta[a]phenanthrene-17,5'-oxolane]-2',3-dione	0.945946	1.3
**143801886**	Canrenone	ligand_74	(8R,10R,17R)-10-ethyl-13-methylspiro[2,6,7,8,9,11,12,14,15,16-decahydro-1H-cyclopenta[a]phenanthrene-17,5'-oxolane]-2',3-dione	0.945946	-5.0
**156853**	Canrenone	ligand_75	(3R,3aS,9aS,9bS)-3a,6-dimethylspiro[1,2,8,9,9a,9b-hexahydrocyclopenta[a]naphthalene-3,5'-oxolane]-2',7-dione	0.944444	-9.9
**44317091**	Canrenone	ligand_76	(11E,13S,17R)-13-methyl-11-prop-2-enylidenespiro[2,6,7,8,12,14,15,16-octahydro-1H-cyclopenta[a]phenanthrene-17,5'-oxolane]-2',3-dione	0.944444	-4.2
**44317102**	Canrenone	ligand_77	CID 44317102	0.944444	-3.8
**44317106**	Canrenone	ligand_78	(11R,13S,17R)-11-ethenyl-13-methylspiro[1,2,6,7,8,11,12,14,15,16-decahydrocyclopenta[a]phenanthrene-17,5'-oxolane]-2',3-dione	0.944444	-3.3
**46241712**	Canrenone	ligand_79	CID 46241712	0.944444	-10.0
**58115347**	Canrenone	ligand_80	(10R,13S,17R)-13-methyl-6-methylidenespiro[2,7,8,9,10,11,12,14,15,16-decahydro-1H-cyclopenta[a]phenanthrene-17,5'-furan]-2',3-dione	0.944444	-6.9
**70805103**	Canrenone	ligand_81	CID 70805103	0.944444	-7.3
**88984902**	Canrenone	ligand_82	(7S,8R,9S,10R,13S,14S,17R)-7-ethenyl-13-methylspiro[1,2,6,7,8,9,10,11,12,14,15,16-dodecahydrocyclopenta[a]phenanthrene-17,5'-oxolane]-2',3-dione	0.944444	-8.6
**88984992**	Canrenone	ligand_83	(10R,13S,17R)-13-methyl-6-methylidenespiro[2,7,8,9,10,11,12,14,15,16-decahydro-1H-cyclopenta[a]phenanthrene-17,5'-oxolane]-2',3-dione	0.944444	-7.0
**91080913**	Canrenone	ligand_84	(5S,7'S)-7'-methylspiro[oxolane-5,6'-pentacyclo[8.8.0.02,7.03,5.011,16]octadeca-11(16),17-diene]-2,14'-dione	0.944444	-6.8
**123556799**	Canrenone	ligand_85	(13S,17R)-13-methyl-6-methylidenespiro[2,4,7,8,9,11,12,14,15,16-decahydro-1H-cyclopenta[a]phenanthrene-17,5'-furan]-2',3-dione	0.944444	-7.8
**101687285**	Canrenone	ligand_86	(8R,9S,10R,13S,14S,17R)-13-methylspiro[2,7,8,9,10,11,12,14,15,16-decahydro-1H-cyclopenta[a]phenanthrene-17,5'-oxolane]-2',3,6-trione	0.944444	-8.4
**44370163**	Canrenone	ligand_87	(17R)-13-methylspiro[1,2,6,7,8,14,15,16-octahydrocyclopenta[a]phenanthrene-17,5'-oxolane]-2',3-dione	0.942857	-8.2

**140340959**	Canrenone	ligand_88	(8R,9S,10R,13S,14S,17S)-10,13-dimethylspiro[3,6,7,8,9,11,12,14,15,16-decahydrocyclopenta[a]phenanthrene-17,5'-oxolane]-2'-one	0.942857	-6.2
**141156062**	Canrenone	ligand_89	(8R,9S,10R,13S,14S,17S)-10,13-dimethylspiro[1,2,3,4,7,8,9,11,12,14,15,16-dodecahydrocyclopenta[a]phenanthrene-17,5'-oxolane]-2'-one	0.942857	-7.7
**142813145**	Canrenone	ligand_90	(17R)-10,13-dimethylspiro[1,2,3,8,9,11,12,14,15,16-decahydrocyclopenta[a]phenanthrene-17,5'-oxolane]-2'-one	0.942857	-6.9
**153754823**	Canrenone	ligand_91	(8R,9S,10R,13S,14S,17S)-10,13-dimethylspiro[1,2,7,8,9,11,12,14,15,16-decahydrocyclopenta[a]phenanthrene-17,5'-oxolane]-2'-one	0.942857	-8.3
**154372571**	Canrenone	ligand_92	(9R,10S,13S,14S,17S)-10,13-dimethylspiro[1,2,3,4,5,6,9,11,12,14,15,16-dodecahydrocyclopenta[a]phenanthrene-17,5'-oxolane]-2'-one	0.942857	-8.2
**9886385**	Canrenone	ligand_93	(7R,8R,9S,10R,13S,14S,17R)-10,13-Dimethyl-7-propylspiro[2,6,7,8,9,11,12,14,15,16-decahydro-1H-cyclopenta[a]phenanthrene-17,5'-oxolane]-2',3-dione	0.921053	-9.4
**11710721**	Canrenone	ligand_94	(7R,8R,10S,13S,14S,17R)-10,13-dimethyl-3,5'-dioxospiro[2,6,7,8,12,14,15,16-octahydro-1H-cyclopenta[a]phenanthrene-17,2'-oxolane]-7-carboxylic acid	0.921053	-9.1
**10045447**	Canrenone	ligand_95	[(9S,10R,13S,14S,17R)-10,13-dimethyl-5'-oxospiro[1,2,9,11,12,14,15,16-octahydrocyclopenta[a]phenanthrene-17,2'-oxolane]-3-yl] acetate	0.921053	-1.8
**16128294**	Canrenone	ligand_96	(7R,10S,13S,17R)-10,13-dimethyl-7-propylspiro[2,6,7,8,12,14,15,16-octahydro-1H-cyclopenta[a]phenanthrene-17,5'-oxolane]-2',3-dione	0.921053	-6.8
**58755480**	Canrenone	ligand_97	(7R,10R,13S,17R)-10,13-dimethyl-3,5'-dioxospiro[2,6,7,8,9,11,12,14,15,16-decahydro-1H-cyclopenta[a]phenanthrene-17,2'-oxolane]-7-carboxylic acid	0.921053	-6.2
**58893040**	Canrenone	ligand_98	(7R,8R,10S,13S,14S,17R)-10,13-dimethyl-7-(2-oxopropanoyl)spiro[2,6,7,8,12,14,15,16-octahydro-1H-cyclopenta[a]phenanthrene-17,5'-oxolane]-2',3-dione	0.921053	-8.2
**59067655**	Canrenone	ligand_99	(7R,10S,13S)-7-ethyl-10,13-dimethylspiro[2,6,7,8,12,14,15,16-octahydro-1H-cyclopenta[a]phenanthrene-17,5'-oxolane]-2',3-dione	0.921053	-6.6

**59409999**	Canrenone	ligand_100	(7R,17R)-7-ethyl-10,13-dimethylspiro[2,6,7,8,9,11,12,14,15,16-decahydro-1H-cyclopenta[a]phenanthrene-17,5'-oxolane]-2',3-dione	0.921053	-5.0
**59410029**	Canrenone	ligand_101	(7R,17R)-7-butyl-10,13-dimethylspiro[2,6,7,8,9,11,12,14,15,16-decahydro-1H-cyclopenta[a]phenanthrene-17,5'-oxolane]-2',3-dione	0.921053	-3.8
**59779298**	Canrenone	ligand_102	(1'R,2'S,3'S,5S,5'S,7'S,10'S,11'R)-3,7',11'-trimethylspiro[oxolane-5,6'-pentacyclo[8.8.0.02,7.03,5.011,16]octadeca-15,17-diene]-2,14'-dione	0.921053	-5.7
**59936883**	Canrenone	ligand_103	(5R)-9-[(2E)-2-(8a-methyl-6-oxo-7,8-dihydro-2H-naphthalen-1-ylidene)ethyl]-8,9-dimethyl-1-oxaspiro[4.4]nonan-2-one	0.921053	-5.3
**70910013**	Canrenone	ligand_104	(1'R,2'S,3'S,5'S,7'S,10'S,11'R)-11'-ethyl-7'-methylspiro[oxolane-5,6'-pentacyclo[8.8.0.02,7.03,5.011,16]octadeca-15,17-diene]-2,14'-dione	0.921053	-5.0
**89510101**	Canrenone	ligand_105	(7S,8R,9S,10R,13S,14S,17R)-7-hexyl-10,13-dimethylspiro[2,6,7,8,9,11,12,14,15,16-decahydro-1H-cyclopenta[a]phenanthrene-17,5'-oxolane]-2',3-dione	0.921053	-6.7
**91130612**	Canrenone	ligand_106	(10S,13S,14S,17R)-10,13-dimethyl-7-(2-oxopropanoyl)spiro[2,4,5,6,12,14,15,16-octahydro-1H-cyclopenta[a]phenanthrene-17,5'-oxolane]-2',3-dione	0.921053	-4.9
**132170633**	Canrenone	ligand_107	(7S,8R,9S,10R,13S,14S,17R)-10,13-dimethyl-7-(2-oxopropyl)spiro[2,6,7,8,9,11,12,14,15,16-decahydro-1H-cyclopenta[a]phenanthrene-17,5'-oxolane]-2',3-dione	0.921053	-8.1
**148892385**	Canrenone	ligand_108	(6S,9S,10R,13S,14S,17R)-13-ethyl-6,10-dimethylspiro[2,6,7,8,9,11,12,14,15,16-decahydro-1H-cyclopenta[a]phenanthrene-17,5'-oxolane]-2',3-dione	0.921053	-6.9
**101917420**	Canrenone	ligand_109	2-[(2S,8R,9S,10R,13S,14S,17R)-10,13-dimethyl-3,5'-dioxospiro[2,8,9,11,12,14,15,16-octahydro-1H-cyclopenta[a]phenanthrene-17,2'-oxolane]-2-yl]acetaldehyde	0.921053	-4.0
**44289937**	Canrenone	ligand_110	(5R)-10',14'-dimethylspiro[oxolane-5,15'-pentacyclo[9.7.0.02,4.05,10.014,18]octadeca-5,8,12-triene]-2,7'-dione	0.918919	-8.0
**59351085**	Canrenone	ligand_111	CID 59351085	0.918919	-5.0
**59612705**	Canrenone	ligand_112	(5S,7'S,11'R)-7'-methyl-17'-methylidenespiro[oxolane-5,6'-pentacyclo[8.8.0.02,7.03,5.011,16]octadec-15-ene]-2,14'-dione	0.918919	5.0

**70805054**	Canrenone	ligand_113	(10R,13S,17R)-7-cyclopropyl-13-methylspiro[1,2,6,7,8,9,10,11,12,14,15,16-dodecahydrocyclopenta[a]phenanthrene-17,5'-oxolane]-2',3-dione	0.918919	-6.4
**91147800**	Canrenone	ligand_114	(10S,13S,17R)-7-acetyl-10,13-dimethylspiro[2,4,5,8,9,11,12,14,15,16-decahydro-1H-cyclopenta[a]phenanthrene-17,5'-oxolane]-2',3-dione	0.918919	-4.3
**141772148**	Canrenone	ligand_115	(1S,4R,5S,8S,20S,21S)-4,21-dimethyl-11-oxapentacyclo[11.7.1.04,8.05,20.017,21]henicosa-16,18-diene-12,15-dione	0.918919	-5.1
**142888388**	Canrenone	ligand_116	(8R,9S,10R,13S,14S,17S)-10,13-dimethylspiro[2,8,9,11,12,14,15,16-octahydro-1H-cyclopenta[a]phenanthrene-17,6'-4,5-didehydro-3H-pyran]-2',3-dione	0.918919	-6.5
**58595789**	Canrenone	ligand_117	(3S,7R,8S,9S,10R,11R,13S)-3,7,10,11,13-pentamethylspiro[1,2,3,4,7,8,9,11,12,14,15,16-dodecahydrocyclopenta[a]phenanthrene-17,5'-oxolane]-2'-one	0.916667	-2.3
**89354381**	Canrenone	ligand_118	(17R)-10,13-dimethyl-3-methylidenespiro[2,6,7,8,12,14,15,16-octahydro-1H-cyclopenta[a]phenanthrene-17,5'-oxolane]-2'-one	0.916667	-5.1
**141927903**	Canrenone	ligand_119	(10R,13S,17R)-10,13-dimethyl-3-methylidenespiro[2,8,9,11,12,14,15,16-octahydro-1H-cyclopenta[a]phenanthrene-17,5'-oxolane]-2'-one	0.916667	-5.8
**141869415**	Canrenone	ligand_120	(10R,13S)-10,13-dimethyl-3-methylidenespiro[2,6,7,8,9,11,12,14,15,16-decahydro-1H-cyclopenta[a]phenanthrene-17,5'-oxolane]-2'-one	0.916667	-7.1
**142119136**	Canrenone	ligand_121	(7R,10S)-7,10-dimethyl-3-methylidenespiro[1,2,6,7,8,12,13,14,15,16-decahydrocyclopenta[a]phenanthrene-17,5'-oxolane]-2'-one	0.916667	-4.8
**142894633**	Canrenone	ligand_122	(8R,10R,13S,17R)-7,10,13-trimethylspiro[1,2,3,4,7,8,9,11,12,14,15,16-dodecahydrocyclopenta[a]phenanthrene-17,5'-oxolane]-2'-one	0.916667	-7.5
**142894651**	Canrenone	ligand_123	(8R,10R,13S,17R)-3,10,13-trimethylspiro[1,2,3,4,7,8,9,11,12,14,15,16-dodecahydrocyclopenta[a]phenanthrene-17,5'-oxolane]-2'-one	0.916667	-5.3
**44359528**	Canrenone	ligand_124	(10R,13S,17R)-10,13-dimethylspiro[5,6,7,8,9,11,12,14,15,16-decahydro-4H-cyclopenta[a]phenanthrene-17,5'-oxolane]-2',3-dione	0.914286	-6.4
**250787**	Canrenone	ligand_125	(8R,9S,10R,13S,14S,17R)-13-methylspiro[2,3,6,7,8,9,10,11,12,14,15,16-dodecahydro-1H-cyclopenta[a]phenanthrene-17,5'-oxolane]-2'-one	0.914286	-9.1

**10272193**	Canrenone	ligand_126	(2'R,7R,8R,10S,13S,14S)-Methyl 10,13-dimethyl-3,5'-dioxo-1,2,3,4',5',6,7,8,10,12,13,14,15,16-tetradecahydro-3'H-spiro[cyclopenta[a]phenanthrene-17,2'-furan]-7-carboxylate	0.897436	-9.2
**15942744**	Canrenone	ligand_127	Mexrenone	0.897436	-7.7
**90674701**	Canrenone	ligand_128	[(8R,9S,10R,13S,14S,15S,17S)-10,13-dimethyl-3,5'-dioxospiro[2,8,9,11,12,14,15,16-octahydro-1H-cyclopenta[a]phenanthrene-17,2'-oxolane]-15-yl] acetate	0.897436	-4.3
**60003085**	Canrenone	ligand_129	methyl (7R,11R,17R)-10,11-dimethyl-3,5'-dioxospiro[1,2,6,7,8,9,11,12,13,14,15,16-dodecahydrocyclopenta[a]phenanthrene-17,2'-oxolane]-7-carboxylate	0.897436	-1.7
**60030886**	Canrenone	ligand_130	4-[(2R)-2,3-dimethyl-3-[(2S,3R,5R,10R,13R,14R,17R)-2,3,10,13,14-pentamethyl-6-oxo-2,3,4,5,9,11,12,15,16,17-decahydro-1H-cyclopenta[a]phenanthren-17-yl]butyl]-4,5,5-trimethyloxolan-2-one	0.897436	5.0
**117615152**	Canrenone	ligand_131	(6R,7R,8R,10R,13S,17R)-13-ethyl-6,7,10-trimethylspiro[2,6,7,8,9,11,12,14,15,16-decahydro-1H-cyclopenta[a]phenanthrene-17,5'-oxolane]-2',3-dione	0.897436	-6.4
**144011952**	Canrenone	ligand_132	(6R,16R,17S)-10-ethenyl-6-ethyl-6,13,16-trimethylspiro[1,2,7,8,12,14,15,16-octahydrocyclopenta[a]phenanthrene-17,5'-oxolane]-2',3-dione	0.897436	-1.6
**145510335**	Canrenone	ligand_133	(7R,17R)-7-iodo-10,13-dimethylspiro[2,6,7,8,9,11,12,14,15,16-decahydro-1H-cyclopenta[a]phenanthrene-17,5'-oxolane]-2',3-dione	0.897436	-1.3
**10386066**	Canrenone	ligand_134	(2S,8R,9S,10R,13S,14S,17R)-10,13-dimethyl-2-(2-oxopropyl)spiro[2,8,9,11,12,14,15,16-octahydro-1H-cyclopenta[a]phenanthrene-17,5'-oxolane]-2',3-dione	0.897436	-2.6
**10451489**	Canrenone	ligand_135	(2S,8R,9S,10R,13S,14S,17R)-10,13-dimethyl-3,5'-dioxospiro[2,8,9,11,12,14,15,16-octahydro-1H-cyclopenta[a]phenanthrene-17,2'-oxolane]-2-carbonitrile	0.897436	-4.0

**21629575**	Canrenone	ligand_136	(2'R,7R,8R,9S,10R,13S,14S)-methyl 10,13-dimethyl-3,5'-dioxo-3,4',5',6,7,8,9,10,11,12,13,14,15,16-tetradecahydro-3'H-spiro[cyclopenta[a]phenanthrene-17,2'-furan]-7-carboxylate	0.897436	-7.3
**23257020**	Canrenone	ligand_137	propan-2-yl (7R,8R,10S,13S,14S,17R)-10,13-dimethyl-3,5'-dioxospiro[2,6,7,8,12,14,15,16-octahydro-1H-cyclopenta[a]phenanthrene-17,2'-oxolane]-7-carboxylate	0.897436	-8.0
**101623288**	Canrenone	ligand_138	(8R,9S,10R,13S,14S,17R)-10,13-dimethyl-7-sulfanylidenespiro[1,2,6,8,9,11,12,14,15,16-decahydrocyclopenta[a]phenanthrene-17,5'-oxolane]-2',3-dione	0.897436	-7.6
**58115331**	Canrenone	ligand_139	(8R,9S,10R,13S,14S,17R)-13-ethylspiro[1,2,8,9,10,11,12,14,15,16-decahydrocyclopenta[a]phenanthrene-17,5'-furan]-2',3-dione	0.891892	-8.1
**70805058**	Canrenone	ligand_140	(10R,13S,17R)-13-ethylspiro[1,2,8,9,10,11,12,14,15,16-decahydrocyclopenta[a]phenanthrene-17,5'-oxolane]-2',3-dione	0.891892	-5.0
**70805106**	Canrenone	ligand_141	(10R,13S,17R)-13-ethylspiro[1,2,6,7,8,9,10,11,12,14,15,16-dodecahydrocyclopenta[a]phenanthrene-17,5'-oxolane]-2',3-dione	0.891892	-6.2
**123454553**	Canrenone	ligand_142	(8R,9S,13S,14S,17R)-13-ethylspiro[1,2,4,8,9,11,12,14,15,16-decahydrocyclopenta[a]phenanthrene-17,5'-furan]-2',3-dione	0.891892	-9.6
**163524875**	Canrenone	ligand_143	(7R,17R)-7,10,13-trimethyl-3-methylidenespiro[2,6,7,8,9,11,12,14,15,16-decahydro-1H-cyclopenta[a]phenanthrene-17,5'-oxolane]-2'-one	0.891892	-5.4
**141908366**	Canrenone	ligand_144	(10R,13S,17R)-10,13-dimethyl-5'-methylidenespiro[2,8,9,11,12,14,15,16-octahydro-1H-cyclopenta[a]phenanthrene-17,2'-oxolane]-3-one	0.888889	-4.4
**101687282**	Canrenone	ligand_145	(8R,9S,10R,13S,14S,17R)-13-methylspiro[4,5,6,7,8,9,10,11,12,14,15,16-dodecahydrocyclopenta[a]phenanthrene-17,5'-oxolane]-2',3-dione	0.885714	-5.9
**16128454**	Canrenone	ligand_146	(10S,13S,17R)-3'-fluoro-10,13-dimethylspiro[2,6,7,8,12,14,15,16-octahydro-1H-cyclopenta[a]phenanthrene-17,5'-oxolane]-2',3-dione	0.875000	-4.6
**58284128**	Canrenone	ligand_147	(3'S,5S,5'S,7'S,9'S,11'R)-9'-fluoro-7',11'-dimethylspiro[oxolane-5,6'-pentacyclo[8.8.0.02,7.03,5.011,16]octadeca-15,17-diene]-2,14'-dione	0.875000	-4.4
**58834230**	Canrenone	ligand_148	(10R,13S,17R)-3'-fluoro-10,13-dimethylspiro[2,8,12,14,15,16-hexahydro-1H-cyclopenta[a]phenanthrene-17,5'-oxolane]-2',3-dione	0.875000	-4.4

**70315830**	Canrenone	ligand_149	(10S,13S,17R)-3'-(fluoromethyl)-10,13-dimethylspiro[2,6,7,8,12,14,15,16-octahydro-1H-cyclopenta[a]phenanthrene-17,5'-oxolane]-2',3-dione	0.875000	-4.6
**451159**	Canrenone	ligand_150	(7R,10R,13S,16R,17S)-7-(3-(18F)fluoranylpropyl)-10,13,16-trimethylspiro[2,6,7,8,9,11,12,14,15,16-decahydro-1H-cyclopenta[a]phenanthrene-17,5'-oxolane]-2',3-dione	0.875000	-9.0
**90679820**	Canrenone	ligand_151	methyl (6R,7S,8R,9S,10R,13S,14S,17R)-6,10,13-trimethyl-3,5'-dioxospiro[2,6,7,8,9,11,12,14,15,16-decahydro-1H-cyclopenta[a]phenanthrene-17,2'-oxolane]-7-carboxylate	0.875000	-7.9
**101917203**	Canrenone	ligand_152	(2R,8R,9S,10R,13S,14S,17R)-2-bromo-10,13-dimethylspiro[2,8,9,11,12,14,15,16-octahydro-1H-cyclopenta[a]phenanthrene-17,5'-oxolane]-2',3-dione	0.875000	-5.0
**58755486**	Canrenone	ligand_153	(5R,9S)-8-[(3R,8aR)-3,8a-dimethyl-6-oxo-1,2,3,4,7,8-hexahydronaphthalen-2-yl]-9-methyl-9-propyl-1-oxaspiro[4.4]nonan-2-one	0.875000	-4.8
**71132575**	Canrenone	ligand_154	methyl 2-[(7S,17R)-10,13-dimethyl-3,5'-dioxospiro[2,6,7,8,12,14,15,16-octahydro-1H-cyclopenta[a]phenanthrene-17,2'-oxolane]-7-yl]acetate	0.875000	-6.3
**71132582**	Canrenone	ligand_155	methyl 2-[(7S,10R,13S,17R)-10,13-dimethyl-3,5',11-trioxospiro[1,2,6,7,8,9,12,14,15,16-decahydrocyclopenta[a]phenanthrene-17,2'-oxolane]-7-yl]acetate	0.875000	-8.5
**71210981**	Canrenone	ligand_156	methyl 2-[(7S,17R)-10,13-dimethyl-3,5'-dioxospiro[2,6,7,8,9,11,12,14,15,16-decahydro-1H-cyclopenta[a]phenanthrene-17,2'-oxolane]-7-yl]acetate	0.875000	-3.9
**71211017**	Canrenone	ligand_157	methyl (7R,8R,10S,13S,14S)-9,10,11,13-tetramethyl-3,5'-dioxospiro[1,2,6,7,8,11,12,14,15,16-decahydrocyclopenta[a]phenanthrene-17,2'-oxolane]-7-carboxylate	0.875000	-4.7
**117876994**	Canrenone	ligand_158	3-[(17R)-10,13-dimethyl-3,5'-dioxospiro[2,8,9,11,12,14,15,16-octahydro-1H-cyclopenta[a]phenanthrene-17,2'-oxolane]-7-yl]propanoic acid	0.875000	-4.8
**141927802**	Canrenone	ligand_159	(5R)-8-(1,8a-dimethyl-6-oxo-1,2,3,4,7,8-hexahydronaphthalen-2-yl)-9-ethyl-9-methyl-1-oxaspiro[4.4]nonan-2-one;ethane	0.875000	-5.5

**10316957**	Canrenone	ligand_160	(2E,8R,9S,10R,13S,14S,17R)-2-(hydroxymethylidene)-10,13-dimethylspiro[1,8,9,11,12,14,15,16-octahydrocyclopenta[a]phenanthrene-17,5'-oxolane]-2',3-dione	0.875000	-3.1
**10807247**	Canrenone	ligand_161	(1R,3aS,3bR,9aR,9bS,11aS)-9a,11a-dimethylspiro[2,3,3a,3b,9,9b,10,11-octahydroindeno[4,5-h]isochromene-1,5'-oxolane]-2',7-dione	0.875000	-6.9
**101916898**	Canrenone	ligand_162	[(Z)-[(8R,9S,10R,13S,14S,17R)-10,13-dimethyl-3,5'-dioxospiro[1,8,9,11,12,14,15,16-octahydrocyclopenta[a]phenanthrene-17,2'-oxolane]-2-ylidene]methyl] acetate	0.875000	2.8
**141927803**	Canrenone	ligand_163	(5R)-8-(1,8a-dimethyl-6-oxo-1,2,3,4,7,8-hexahydronaphthalen-2-yl)-9-ethyl-9-methyl-1-oxaspiro[4.4]nonan-2-one	0.875000	-6.4
**11523200**	Canrenone	ligand_164	(1'R,2'S,3'S,5S,5'S,7'S,10'S,11'R)-7'-ethylspiro[oxolane-5,6'-pentacyclo[8.8.0.02,7.03,5.011,16]octadeca-15,17-diene]-2,14'-dione	0.868421	-7.2
**44131734**	Canrenone	ligand_165	(10R,13S,17R)-13-ethyl-6-methylidenespiro[2,7,8,9,10,11,12,14,15,16-decahydro-1H-cyclopenta[a]phenanthrene-17,5'-furan]-2',3-dione	0.868421	-6.6
**70805242**	Canrenone	ligand_166	CID 70805242	0.868421	-7.1
**88984904**	Canrenone	ligand_167	(7S,8R,9S,10R,13S,14S,17R)-7-ethenyl-13-ethylspiro[1,2,6,7,8,9,10,11,12,14,15,16-dodecahydrocyclopenta[a]phenanthrene-17,5'-oxolane]-2',3-dione	0.868421	-8.3
**88984928**	Canrenone	ligand_168	(10R,13S,17R)-13-ethyl-6-methylidenespiro[2,7,8,9,10,11,12,14,15,16-decahydro-1H-cyclopenta[a]phenanthrene-17,5'-oxolane]-2',3-dione	0.868421	-6.7
**90729068**	Canrenone	ligand_169	(3'S,5S,5'S,7'S)-7'-ethylspiro[oxolane-5,6'-pentacyclo[8.8.0.02,7.03,5.011,16]octadeca-11(16),17-diene]-2,14'-dione	0.868421	-6.2
**117613146**	Canrenone	ligand_170	(8R,10R,13S,17R)-13-ethyl-3,10-dimethylspiro[1,2,7,8,9,11,12,14,15,16-decahydrocyclopenta[a]phenanthrene-17,5'-oxolane]-2'-one	0.868421	-5.1
**123257138**	Canrenone	ligand_171	(13S,17R)-13-ethyl-6-methylidenespiro[2,4,7,8,9,11,12,14,15,16-decahydro-1H-cyclopenta[a]phenanthrene-17,5'-furan]-2',3-dione	0.868421	-7.8
**11088332**	Canrenone	ligand_172	6-Dehydronandrolone acetate	0.864865	-8.9
**154466145**	Canrenone	ligand_173	(8S,13S,14S)-6,13-dimethylspiro[1,2,6,7,8,11,12,14,15,16-decahydrocyclopenta[a]phenanthrene-17,2'-oxolane]-3-one	0.864865	-8.0

**9818762**	Canrenone	ligand_174	(17R)-10,13-dimethylspiro[2,8,9,11,12,14,15,16-octahydro-1H-cyclopenta[a]phenanthrene-17,2'-oxolane]-3-one	0.864865	-8.0
**90679821**	Canrenone	ligand_175	methyl (7S,8R,9S,10R,13S,14S,17R)-10,13-dimethyl-6-methylidene-3,5'-dioxospiro[1,2,7,8,9,11,12,14,15,16-decahydrocyclopenta[a]phenanthrene-17,2'-oxolane]-7-carboxylate	0.853659	-7.9
**58700855**	Canrenone	ligand_176	methyl (7R,17R)-10,13-dimethyl-3,5',12-trioxospiro[1,2,6,7,8,14,15,16-octahydrocyclopenta[a]phenanthrene-17,2'-oxolane]-7-carboxylate	0.853659	-1.2
**163988682**	Canrenone	ligand_177	(7R,9R,17R)-9-iodo-7,10,13-trimethylspiro[1,2,6,7,8,11,12,14,15,16-decahydrocyclopenta[a]phenanthrene-17,5'-oxolane]-2',3-dione	0.853659	-3.0
**89555397**	Canrenone	ligand_178	(8R,10R,13S,17R)-13-ethyl-3-methoxy-10-methylspiro[1,2,7,8,9,11,12,14,15,16-decahydrocyclopenta[a]phenanthrene-17,5'-oxolane]-2'-one	0.850000	-5.5
**59612706**	Canrenone	ligand_179	(5S,7'S,11'R)-7'-ethyl-17'-methylidenespiro[oxolane-5,6'-pentacyclo[8.8.0.02,7.03,5.011,16]octadec-15-ene]-2,14'-dione	0.846154	-4.6
**70805121**	Canrenone	ligand_180	(5R,10'R,14'S)-14'-ethylspiro[oxolane-5,15'-pentacyclo[9.7.0.02,4.05,10.014,18]octadec-5-ene]-2,7'-dione	0.846154	-6.0
**3065098**	Canrenone	ligand_181	6,7-Dehydro Norethindrone Acetate	0.842105	-5.3
**59902248**	Canrenone	ligand_182	[(10R,13S,17S)-10,13-dimethyl-3-oxo-1,2,8,9,11,12,14,15,16,17-decahydrocyclopenta[a]phenanthren-17-yl] acetate	0.842105	-6.8
**12832214**	Canrenone	ligand_183	17beta-Acetoxy-17alpha-methylandrosta-4,6-dien-3-one	0.842105	-7.1
**69734858**	Canrenone	ligand_184	[(8R,9S,10R,11S,13S,14S,17S)-11,13-dimethyl-3-oxo-2,8,9,10,11,12,14,15,16,17-decahydro-1H-cyclopenta[a]phenanthren-17-yl] acetate	0.842105	-8.4
**23618543**	Canrenone	ligand_185	17-O-Acetyl Normethandrone	0.842105	-8.0
**131667591**	Canrenone	ligand_186	CID 131667591	0.842105	-6.9
**145714468**	Canrenone	ligand_187	[(8R,9S,10R,13R,14S,17S)-10,13-dimethyl-3-oxo-1,2,8,9,11,12,14,15,16,17-decahydrocyclopenta[a]phenanthren-17-yl] 3-cyclopentylpropanoate	0.842105	-7.3
**22849366**	Canrenone	ligand_188	(8S,13S,14S)-13-methylspiro[1,2,6,7,8,11,12,14,15,16-decahydrocyclopenta[a]phenanthrene-17,2'-oxolane]-3-one	0.837838	-7.6

**70827795**	Canrenone	ligand_189	(10R,13S,17R)-13-methylspiro[1,2,8,9,10,11,12,14,15,16-decahydrocyclopenta[a]phenanthrene-17,2'-oxolane]-3-one	0.837838	-5.8
**44317152**	Canrenone	ligand_190	(11S,13S,17R)-11-(3-methoxypropyl)-13-methylspiro[1,2,6,7,8,11,12,14,15,16-decahydrocyclopenta[a]phenanthrene-17,5'-oxolane]-2',3-dione	0.833333	-3.7
**44317307**	Canrenone	ligand_191	(11S,13S,17R)-13-methyl-11-[3-[(2-methylpropan-2-yl)oxy]propyl]spiro[1,2,6,7,8,11,12,14,15,16-decahydrocyclopenta[a]phenanthrene-17,5'-oxolane]-2',3-dione	0.833333	-2.5
**70863463**	Canrenone	ligand_192	(7R,17R)-10,13-dimethyl-7-[3-[(2-methylpropan-2-yl)oxy]propyl]spiro[2,6,7,8,9,11,12,14,15,16-decahydro-1H-cyclopenta[a]phenanthrene-17,5'-oxolane]-2',3-dione	0.833333	-6.1
**71137495**	Canrenone	ligand_193	methyl 2-[(7S,10S,13S,17R)-10,13-dimethyl-3,5',12-trioxospiro[1,2,6,7,8,14,15,16-octahydrocyclopenta[a]phenanthrene-17,2'-oxolane]-7-yl]acetate	0.833333	-7.1
**117876992**	Canrenone	ligand_194	(17R)-10,13-dimethyl-7-[3-[(2-methylpropan-2-yl)oxy]propyl]spiro[2,8,9,11,12,14,15,16-octahydro-1H-cyclopenta[a]phenanthrene-17,5'-oxolane]-2',3-dione	0.833333	-5.3
**117876993**	Canrenone	ligand_195	(7S,17R)-10,13-dimethyl-7-[2-[(2-methylpropan-2-yl)oxy]ethyl]spiro[2,6,7,8,9,11,12,14,15,16-decahydro-1H-cyclopenta[a]phenanthrene-17,5'-oxolane]-2',3-dione	0.833333	-2.5
**142945936**	Canrenone	ligand_196	methyl (7R,17R)-10,13-dimethyl-1-(2-methylcyclopropyl)-3,5'-dioxospiro[2,6,7,8,12,14,15,16-octahydro-1H-cyclopenta[a]phenanthrene-17,2'-oxolane]-7-carboxylate	0.833333	-3.6
**23257019**	Canrenone	ligand_197	ethyl (7R,8R,10S,13S,14S,17R)-10,13-dimethyl-3,5'-dioxospiro[2,6,7,8,12,14,15,16-octahydro-1H-cyclopenta[a]phenanthrene-17,2'-oxolane]-7-carboxylate	0.833333	-7.9
**102303**	Canrenone	ligand_198	Aldona ethyl enol ether	0.829268	-3.3

**122683356**	Canrenone	ligand_199	(3R,3aS,6S,7R,7aS)-6-[(1R)-2-ethyl-1-methyl-4-oxocyclohex-2-en-1-yl]-3a-methyl-7-propan-2-ylspiro[2,4,5,6,7,7a-hexahydro-1H-indene-3,5'-oxolane]-2'-one	0.829268	-4.2
**10947441**	Canrenone	ligand_200	Methyl androst-4,6-diene-3one-17beta-carboxylate	0.820513	-8.6
**12951781**	Canrenone	ligand_201	[(8R,9S,10R,13S,14S,17S)-10,13,17-trimethyl-6-methylidene-3-oxo-1,2,7,8,9,11,12,14,15,16-decahydrocyclopenta[a]phenanthren-17-yl] acetate	0.820513	-7.6
**138478913**	Canrenone	ligand_202	[(8R,9S,10R,14S,17R)-17-acetyl-6,10-dimethyl-3-oxo-1,2,8,9,11,12,13,14,15,16-decahydrocyclopenta[a]phenanthren-17-yl] acetate	0.820513	-2.8
**142260476**	Canrenone	ligand_203	[(7R,10S,13S,17S)-7-ethyl-10,17-dimethyl-3-oxo-1,2,6,7,8,12,13,14,15,16-decahydrocyclopenta[a]phenanthren-17-yl] propanoate	0.820513	-6.5
**9905888**	Canrenone	ligand_204	(1'S,2R,2'S,3'S,5'R,11'R,12'S,16'S)-2',16'-dimethylspiro[oxolane-2,15'-pentacyclo[9.7.0.02,8.03,5.012,16]octadeca-7,9-diene]-6'-one	0.820513	-9.1
**11921133**	Canrenone	ligand_205	[(8S,9R,10R,13R,14R,16R)-16-acetyl-10,13-dimethyl-3-oxo-9,11,12,14,15,17-hexahydro-8H-cyclopenta[a]phenanthren-16-yl] acetate	0.820513	-4.7
**16128930**	Canrenone	ligand_206	(7R,10S,13S,17R)-7-(methoxymethyl)-10,13-dimethylspiro[2,6,7,8,12,14,15,16-octahydro-1H-cyclopenta[a]phenanthrene-17,5'-oxolane]-2',3-dione	0.813953	-6.3
**11683**	Canrenone	ligand_207	Megestrol Acetate	0.800000	-3.9
**91668**	Canrenone	ligand_208	Nomegestrol acetate	0.800000	-6.2
**11876390**	Canrenone	ligand_209	[(8R,9S,10R,13S,14S,17R)-17-acetyl-10,13-dimethyl-3-oxo-9,11,12,14,15,16-hexahydro-8H-cyclopenta[a]phenanthren-17-yl] acetate	0.800000	-5.0
**200136**	Canrenone	ligand_210	Melengestro acetate	0.800000	-4.6
**14333869**	Canrenone	ligand_211	17beta-Propanoyloxyandrosta-4,6-dien-3-one	0.800000	-7.5
**29983908**	Canrenone	ligand_212	2alpha,6-Dimethyl-3,20-dioxopregna-4,6-dien-17-yl acetate	0.800000	-5.2
**14999811**	Canrenone	ligand_213	1-Dehydromegesterol acetate	0.800000	-4.1
**91488794**	Canrenone	ligand_214	[(8R,9S,10R,13S,14S,17R)-17-acetyl-2,10,13-trimethyl-3-oxo-2,8,9,11,12,14,15,16-octahydro-1H-cyclopenta[a]phenanthren-17-yl] acetate	0.800000	-2.4

**11070200**	Canrenone	ligand_215	[(8R,9S,10R,13S,14S,16S,17R)-17-acetyl-10,13,16-trimethyl-3-oxo-2,8,9,11,12,14,15,16-octahydro-1H-cyclopenta[a]phenanthren-17-yl] cyclopentanecarboxylate	0.800000	-6.7
**57161907**	Canrenone	ligand_216	[(8S,9S,10R,13S,14S,15R,17R)-17-acetyl-6,13,15-trimethyl-3-oxo-1,2,8,9,10,11,12,14,15,16-decahydrocyclopenta[a]phenanthren-17-yl] acetate	0.800000	-4.0
**59048314**	Canrenone	ligand_217	[(17R)-17-acetyl-1,6,10,13-tetramethyl-3-oxo-9,11,12,14,15,16-hexahydro-8H-cyclopenta[a]phenanthren-17-yl] acetate	0.800000	-3.4
**70849723**	Canrenone	ligand_218	[(10R,13S,17S)-10-ethyl-13-methyl-3-oxo-1,2,8,9,11,12,14,15,16,17-decahydrocyclopenta[a]phenanthren-17-yl] acetate	0.800000	-7.4
**70859686**	Canrenone	ligand_219	2-[(17R)-13-methyl-3-oxo-2,8,9,10,11,12,14,15,16,17-decahydro-1H-cyclopenta[a]phenanthren-17-yl]ethyl acetate	0.800000	-6.5
**141869383**	Canrenone	ligand_220	[(17S)-10,13,17-trimethyl-3-oxo-2,8,9,11,12,14,15,16-octahydro-1H-cyclopenta[a]phenanthren-17-yl] propanoate	0.800000	-5.8
**142945934**	Canrenone	ligand_221	[(10R,13S,17S)-10,13,17-trimethyl-3-oxo-2,8,12,14,15,16-hexahydro-1H-cyclopenta[a]phenanthren-17-yl] propanoate	0.800000	-5.7
**10596200**	Canrenone	ligand_222	[(8R,9S,10R,13S,14S,16S,17R)-17-acetyl-10,13,16-trimethyl-3-oxo-2,8,9,11,12,14,15,16-octahydro-1H-cyclopenta[a]phenanthren-17-yl] acetate	0.800000	-6.5
**22798397**	Canrenone	ligand_223	[(8R,9S,10R,13S,14S,17R)-17-acetyl-13-methyl-3-oxo-1,2,8,9,10,11,12,14,15,16-decahydrocyclopenta[a]phenanthren-17-yl] acetate	0.800000	-3.9
**141963620**	Canrenone	ligand_224	[(10R,13S,17R)-17-ethyl-6,10,13-trimethyl-3-oxo-2,8,9,11,12,14,15,16-octahydro-1H-cyclopenta[a]phenanthren-17-yl] acetate	0.800000	-5.9
**104627**	Canrenone	ligand_225	Ethyl 3-oxopregna-4,6-diene-21,17alpha-carbolactone-21-carboxylate	0.795455	-1.4
**158384295**	Canrenone	ligand_226	[(7R,10S,13S,17R)-7-methoxycarbonyl-10,13-dimethyl-3-oxospiro[2,6,7,8,12,14,15,16-octahydro-1H-cyclopenta[a]phenanthrene-17,5'-oxolane]-2'-ylidene]oxidanium	0.795455	-7.0
**10073141**	Canrenone	ligand_227	ethyl (1'R,2R,2'S,5'S,9'S,10'R,20'R)-1',5'-dimethyl-5,17'-dioxospiro[oxolane-2,6'-pentacyclo[11.8.0.02,10.05,9.015,20]henicosa-11,13,15-triene]-18'-carboxylate	0.795455	15.6

**101916899**	Canrenone	ligand_228	(2Z,8R,9S,10R,13S,14S,17R)-10,13-dimethyl-2-(2-methylpropoxymethylidene)spiro[1,8,9,11,12,14,15,16-octahydrocyclopenta[a]phenanthrene-17,5'-oxolane]-2',3-dione	0.795455	4.3
**68144641**	Canrenone	ligand_229	[(8R,9S,10R,14S,17S)-13-ethyl-3-oxo-2,8,9,10,11,12,14,15,16,17-decahydro-1H-cyclopenta[a]phenanthren-17-yl] acetate	0.794872	-8.0
**44317116**	Canrenone	ligand_230	(11S,13S,17R)-11-(3-hydroxypropyl)-13-methylspiro[1,2,6,7,8,11,12,14,15,16-decahydrocyclopenta[a]phenanthrene-17,5'-oxolane]-2',3-dione	0.790698	-3.7
**10994065**	Canrenone	ligand_231	[(8R,9S,10R,13S,14S,16S,17R)-17-acetyl-10,13,16-trimethyl-3-oxo-2,8,9,11,12,14,15,16-octahydro-1H-cyclopenta[a]phenanthren-17-yl] 2-cyclopentylacetate	0.780488	-5.3
**12951783**	Canrenone	ligand_232	[(8R,9S,10R,13S,14S,17R)-17-ethenyl-10,13-dimethyl-6-methylidene-3-oxo-1,2,7,8,9,11,12,14,15,16-decahydrocyclopenta[a]phenanthren-17-yl] propanoate	0.780488	-5.2
**57106481**	Canrenone	ligand_233	[(8R,9S,10R,13S,14S,17R)-17-acetyl-10,13-dimethyl-1,2-dimethylidene-3-oxo-9,11,12,14,15,16-hexahydro-8H-cyclopenta[a]phenanthren-17-yl] acetate	0.780488	-4.2
**154152594**	Canrenone	ligand_234	[(8R,9S,10R,13S,14S,17R)-10,13-dimethyl-3-oxo-17-prop-2-enoyl-2,8,9,11,12,14,15,16-octahydro-1H-cyclopenta[a]phenanthren-17-yl] acetate	0.780488	-4.6
**10972410**	Canrenone	ligand_235	[(8R,9S,10R,13S,14S,16S,17R)-17-acetyl-10,13,16-trimethyl-3-oxo-9,11,12,14,15,16-hexahydro-8H-cyclopenta[a]phenanthren-17-yl] 2-cyclopentylacetate	0.780488	-4.8
**141963634**	Canrenone	ligand_236	[(10R,13S,17R)-17-ethyl-6,10,13-trimethyl-16-methylidene-3-oxo-1,2,8,9,11,12,14,15-octahydrocyclopenta[a]phenanthren-17-yl] acetate	0.780488	-3.0
**10691324**	Canrenone	ligand_237	[(8R,9S,10R,13S,14S,16S,17R)-17-acetyl-10,13,16-trimethyl-3-oxo-9,11,12,14,15,16-hexahydro-8H-cyclopenta[a]phenanthren-17-yl] acetate	0.775000	-6.1
**121231019**	Canrenone	ligand_238	[(13S,17R)-13-Ethyl-17-ethynyl-3-oxo-1,2,8,9,10,11,12,14,15,16-decahydrocyclopenta[a]phenanthren-17-yl] acetate	0.775000	-6.6
**70827797**	Canrenone	ligand_239	(10R,13S,17R)-13-ethylspiro[1,2,8,9,10,11,12,14,15,16-decahydrocyclopenta[a]phenanthrene-17,2'-oxolane]-3-one	0.769231	-6.0

**23265655**	Canrenone	ligand_240	Megestrol caproate	0.761905	-4.5
**91349484**	Canrenone	ligand_241	[(8R,9S,10R,13S,14S,17R)-17-acetyl-2,10,13-trimethyl-3-oxo-2,8,9,11,12,14,15,16-octahydro-1H-cyclopenta[a]phenanthren-17-yl] hexanoate	0.761905	-4.2
**91569852**	Canrenone	ligand_242	[(8R,9S,10R,13S,14S,17R)-17-acetyl-10,13,16-trimethyl-3-oxo-2,8,9,11,12,14,15,16-octahydro-1H-cyclopenta[a]phenanthren-17-yl] hexanoate	0.761905	-4.4
**101440938**	Canrenone	ligand_243	[(8R,9S,10R,13S,14S,16S,17R)-17-acetyl-10,13,16-trimethyl-3-oxo-2,8,9,11,12,14,15,16-octahydro-1H-cyclopenta[a]phenanthren-17-yl] propanoate	0.761905	-6.6
**10432624**	Canrenone	ligand_244	[(8R,9S,10R,13S,14S,16S,17R)-17-acetyl-10,13,16-trimethyl-3-oxo-2,8,9,11,12,14,15,16-octahydro-1H-cyclopenta[a]phenanthren-17-yl] pentanoate	0.761905	-6.3
**11729569**	Canrenone	ligand_245	[(8R,9S,10R,13S,14S,16S,17R)-17-acetyl-10,13,16-trimethyl-3-oxo-9,11,12,14,15,16-hexahydro-8H-cyclopenta[a]phenanthren-17-yl] pentanoate	0.761905	-6.3
**9823810**	Canrenone	ligand_246	[(1S,10R,13S,17R)-17-acetyl-1,6,10,13-tetramethyl-3-oxo-2,8,9,11,12,14,15,16-octahydro-1H-cyclopenta[a]phenanthren-17-yl] butanoate	0.761905	-2.7
**9887922**	Canrenone	ligand_247	[(1S,10R,13S,17R)-17-acetyl-1,6,10,13-tetramethyl-3-oxo-2,8,9,11,12,14,15,16-octahydro-1H-cyclopenta[a]phenanthren-17-yl] propanoate	0.761905	-2.9
**69871345**	Canrenone	ligand_248	[(1S,8R,9S,10R,13S,14S,17S)-17-acetyl-6,10,13-trimethyl-3-oxo-1-prop-2-enyl-2,8,9,11,12,14,15,16-octahydro-1H-cyclopenta[a]phenanthren-17-yl] acetate	0.761905	-3.3
**70184310**	Canrenone	ligand_249	[(8R,9S,10R,13S,14S,17S)-17-acetyl-6-but-3-enyl-10,13-dimethyl-3-oxo-2,8,9,11,12,14,15,16-octahydro-1H-cyclopenta[a]phenanthren-17-yl] acetate	0.761905	-5.9
**70625902**	Canrenone	ligand_250	3-[(8R,9S,10R,13S,14S,17R)-17-acetyloxy-13-methyl-3-oxo-1,2,8,9,10,11,12,14,15,16-decahydrocyclopenta[a]phenanthren-17-yl]propyl acetate	0.761905	-4.1
**70626441**	Canrenone	ligand_251	3-[(8R,9S,10R,13S,14S,17R)-17-acetyloxy-10,13-dimethyl-3-oxo-2,8,9,11,12,14,15,16-octahydro-1H-cyclopenta[a]phenanthren-17-yl]propyl acetate	0.761905	-4.6

**70636320**	Canrenone	ligand_252	[(8R,9S,10R,13S,14S,17R)-17-ethyl-6,10,13-trimethyl-2,3-dioxo-1,8,9,11,12,14,15,16-octahydrocyclopenta[a]phenanthren-17-yl] acetate	0.761905	-3.5
**140396738**	Canrenone	ligand_253	[(8R,9S,10R,13S,14S,17R)-10,13-dimethyl-3-oxo-17-propanoyl-2,8,9,11,12,14,15,16-octahydro-1H-cyclopenta[a]phenanthren-17-yl] acetate	0.761905	-4.6
**10596905**	Canrenone	ligand_254	[(8R,9S,10R,13S,14S,16S,17R)-17-acetyl-10,13,16-trimethyl-3-oxo-9,11,12,14,15,16-hexahydro-8H-cyclopenta[a]phenanthren-17-yl] propanoate	0.761905	-6.6
**101434477**	Canrenone	ligand_255	[(8R,9S,10R,13S,14S,17R)-17-acetyl-6,10,13-trimethyl-3-oxo-2,8,9,11,12,14,15,16-octahydro-1H-cyclopenta[a]phenanthren-17-yl] propanoate	0.761905	-3.6
**101434478**	Canrenone	ligand_256	[(8R,9S,10R,13S,14S,17R)-17-acetyl-6,10,13-trimethyl-3-oxo-2,8,9,11,12,14,15,16-octahydro-1H-cyclopenta[a]phenanthren-17-yl] butanoate	0.761905	-3.3
**101434479**	Canrenone	ligand_257	[(8R,9S,10R,13S,14S,17R)-17-acetyl-6,10,13-trimethyl-3-oxo-2,8,9,11,12,14,15,16-octahydro-1H-cyclopenta[a]phenanthren-17-yl] pentanoate	0.761905	-3.0
**656615**	Canrenone	ligand_258	Canrenoic acid	0.744186	-7.5
**24776140**	Canrenone	ligand_259	Canrenoate	0.744186	-7.5
**57188245**	Canrenone	ligand_260	[(8R,9S,10R,13S,14S,17R)-17-acetyl-10,13-dimethyl-1,2-dimethylidene-3-oxo-9,11,12,14,15,16-hexahydro-8H-cyclopenta[a]phenanthren-17-yl] butanoate	0.744186	-2.6
**88654282**	Canrenone	ligand_261	[(8R,9S,10R,13S,14S,17R)-17-acetyl-10,13-dimethyl-1,2-dimethylidene-3-oxo-9,11,12,14,15,16-hexahydro-8H-cyclopenta[a]phenanthren-17-yl] propanoate	0.744186	-2.6
**88654302**	Canrenone	ligand_262	[(8R,9S,10R,13S,14S,17R)-17-acetyl-10,13-dimethyl-1,2-dimethylidene-3-oxo-9,11,12,14,15,16-hexahydro-8H-cyclopenta[a]phenanthren-17-yl] hexanoate	0.744186	-1.6
**70626772**	Canrenone	ligand_263	17-Acetoxy-11beta-fluoro-4,6-pregnadiene-3,20-dione	0.727273	-4.6
**24843102**	Canrenone	ligand_264	17-alpha-Acetoxy-6-dehydro-21-fluoro-6-methylprogesterone	0.727273	-4.7
**57926059**	Canrenone	ligand_265	3-[(8R,9S,10R,13S,14S,17R)-17-hydroxy-10,13,14-trimethyl-3-oxo-1,2,8,9,11,12,15,16-octahydrocyclopenta[a]phenanthren-17-yl]propanoate	0.727273	-7.5

**142825532**	Canrenone	ligand_266	1-[(10R,13S,17R)-17-acetyloxy-10,13-dimethyl-3-oxo-2,8,9,11,12,14,15,16-octahydro-1H-cyclopenta[a]phenanthren-17-yl]ethylideneoxidanium	0.727273	-5.2
**144699060**	Canrenone	ligand_267	3-[(8S,9S,10R,13S,14S,17R)-17-hydroxy-10,13-dimethyl-3-oxo-2,8,9,11,12,14,15,16-octahydro-1H-cyclopenta[a]phenanthren-17-yl]propanoyloxidanium	0.727273	-6.6

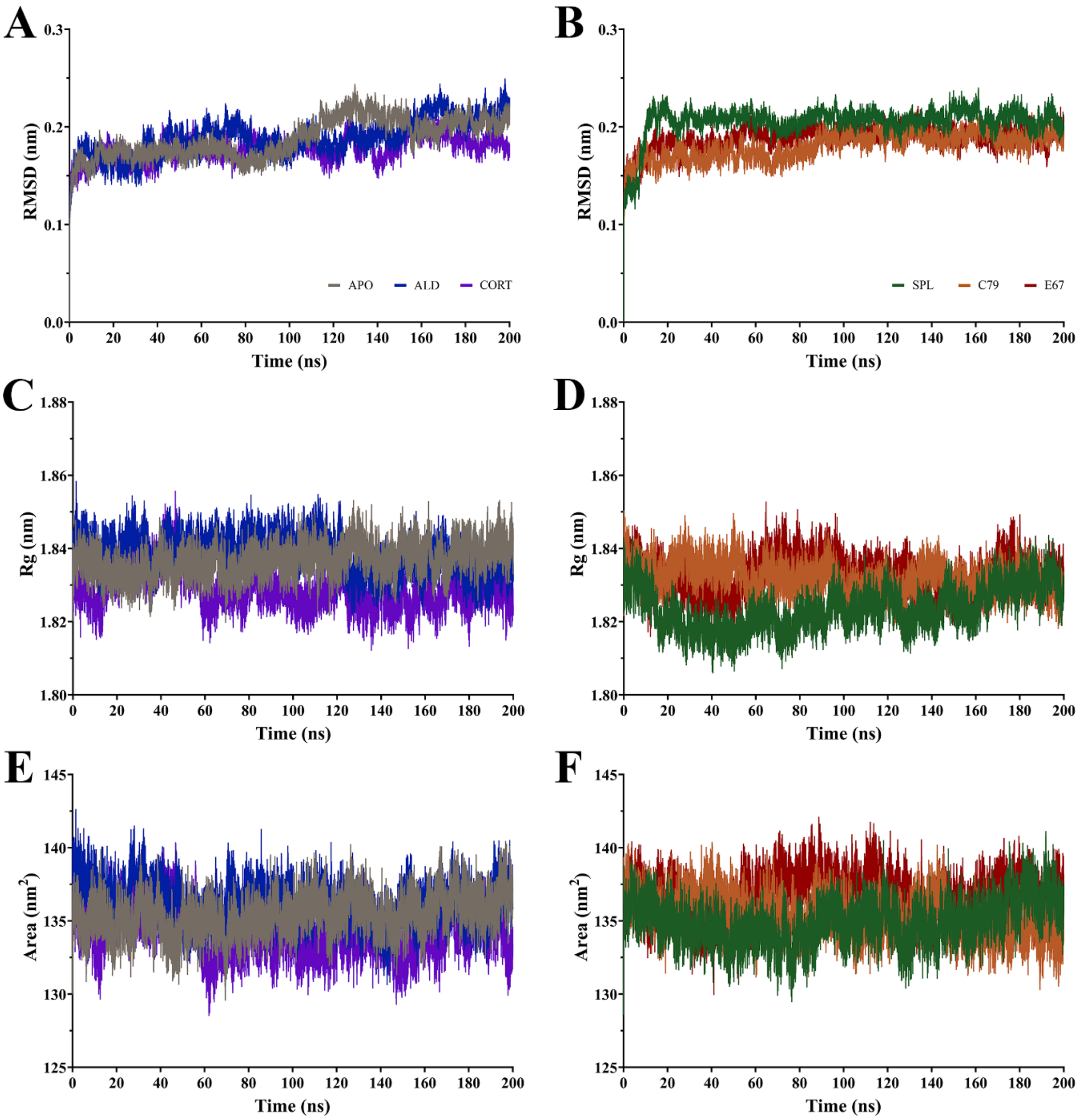

Figure S1. Molecular dynamics (MD) simulations of MR LBD bound with ligands. A-B) Root meant square deviation (RMSD) of protein backbone. C-D) Radius of gyration (Rg) of protein backbone. E-F) Solvent accessible surface area (SASA) of protein surface. Data is shown as an average of three runs, with the ligand free protein (APO) shown in grey, and the MR LBD bound with aldosterone (ALD) in blue, cortisol (CORT) in purple), spironolactone (SPL) in green, C79 in orange, and E67 in red.

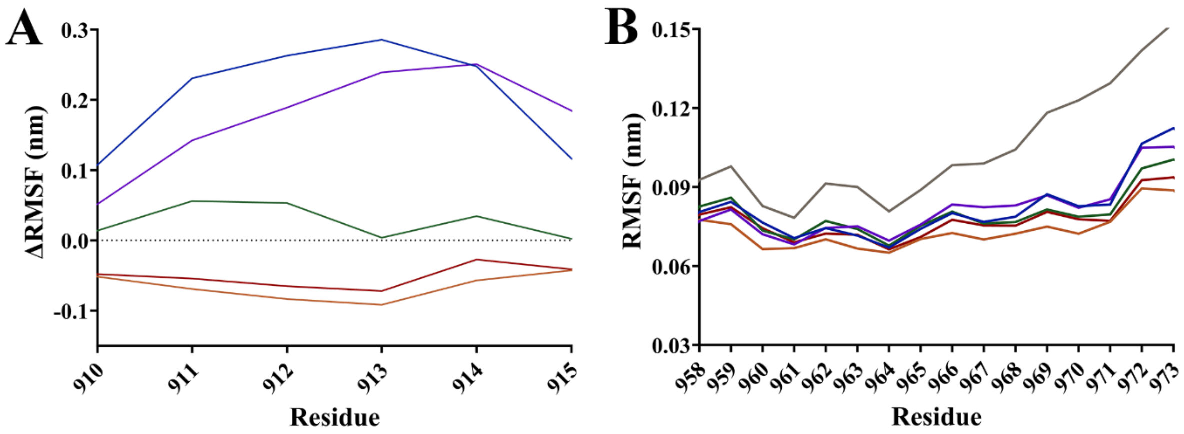

Figure S2: RMSF of protein backbone for regions of interest for MR bound to ligands. A) Difference in RMSF of protein backbone for residues 910 to 915 with APO values subtracted from ligand-bound MR LBD. B) RMSF for helix 12 residues 958 to 973. Data is shown as an average of three independent runs following equilibration of the trajectory. Data for the ligand free protein (APO) shown in grey, and the MR LBD bound with aldosterone (ALD) in blue, cortisol (CORT) in purple), spironolactone (SPL) in green, C79 in orange, and E67 in red.

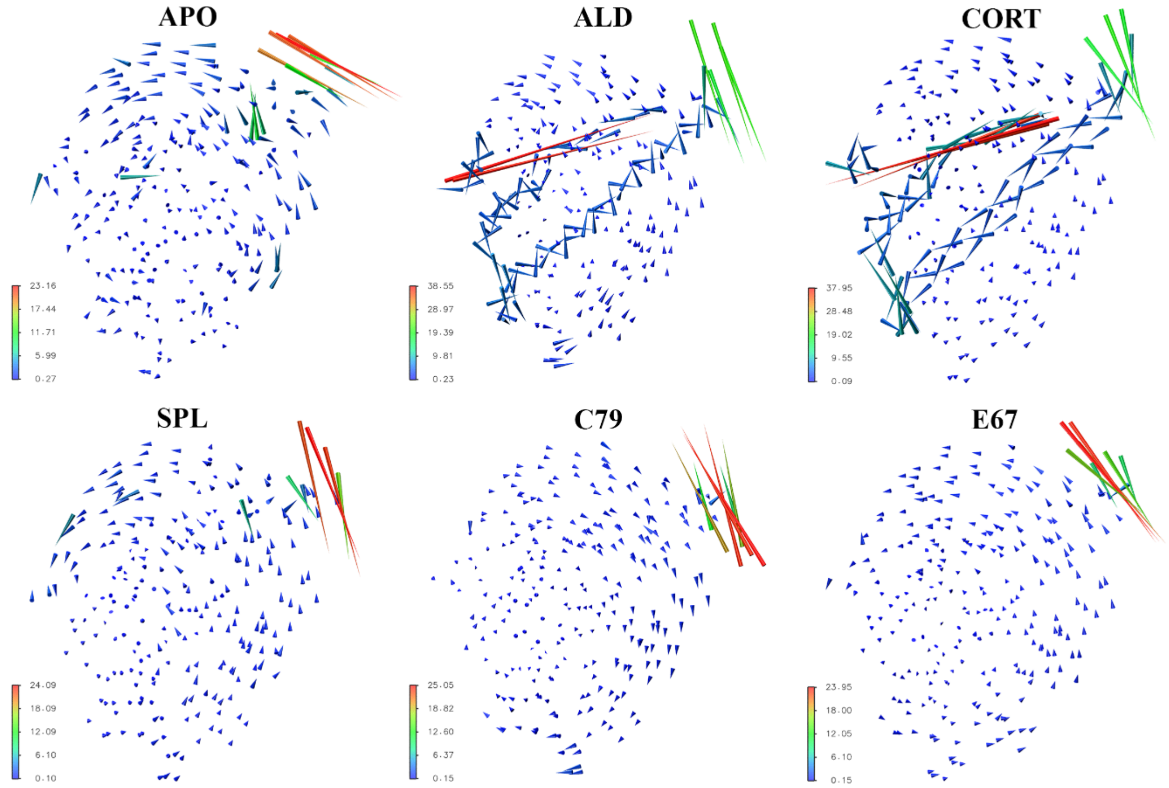

Figure S3: Porcupine plots showing movement along the first principal component (PC1) of MR LBD in response to ligand binding.

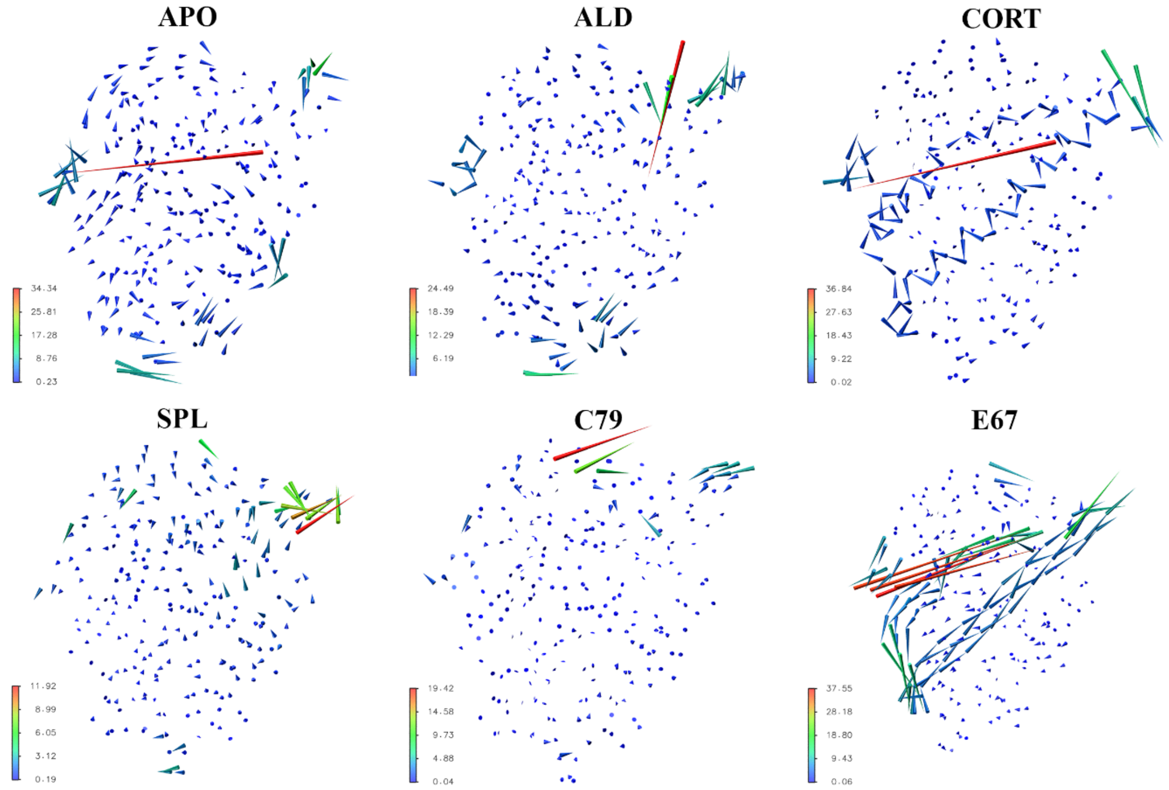

Figure S4: Porcupine plots showing movement along the second principal component (PC2) of MR LBD in response to ligand binding.

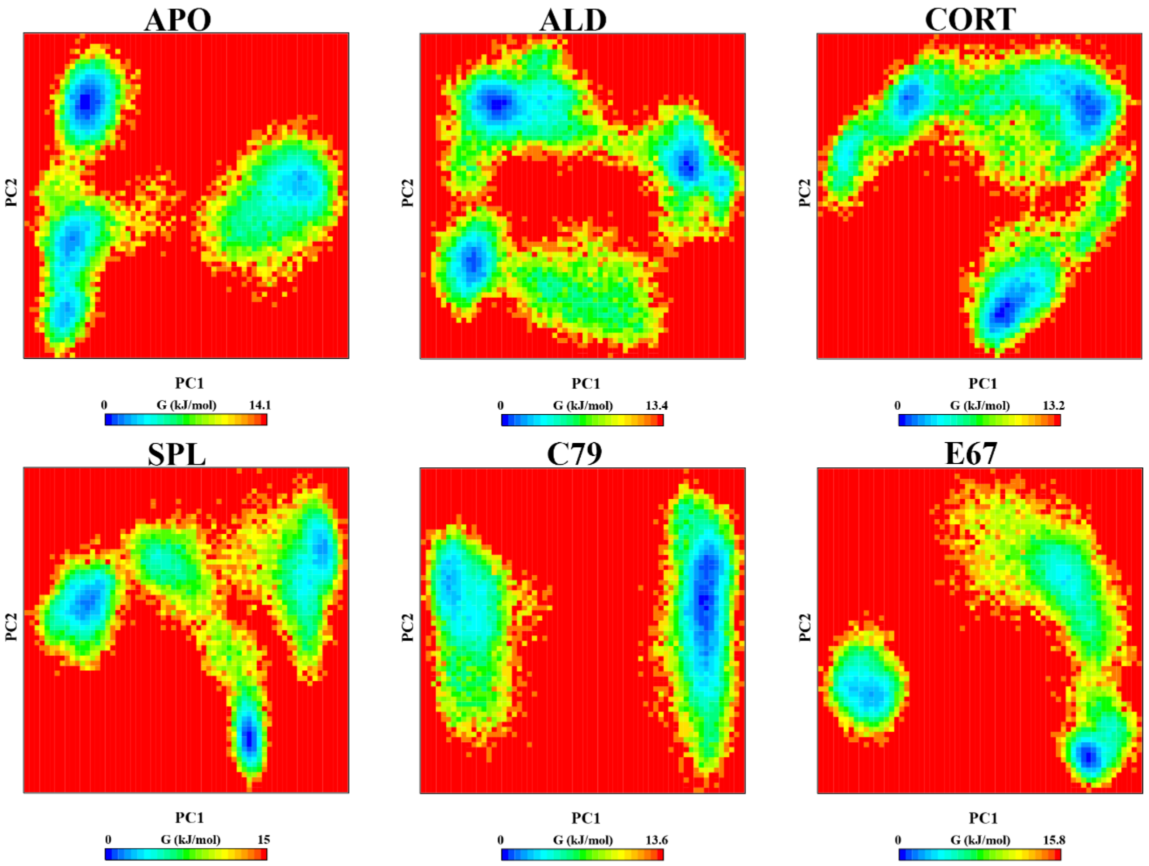

Figure S5: Free energy landscape (FEL) plots calculated from the first two principal components (PC1 and PC2) of the MR LBD backbone in response to ligand binding.
